# Detection of *Fusarium* infected seeds of cereal plants by the fluorescence method

**DOI:** 10.1371/journal.pone.0267912

**Published:** 2022-07-01

**Authors:** Alexey Dorokhov, Maksim Moskovskiy, Mikhail Belyakov, Alexander Lavrov, Victor Khamuev

**Affiliations:** Federal Scientific Agroengineering Center VIM, Moscow, Russia; Murdoch University, AUSTRALIA

## Abstract

Infection of seeds of cereal plants with fusarium affects their optical luminescent properties. The spectral characteristics of excitation (absorption) in the range of 180–700 nm of healthy and infected seeds of wheat, barley and oats were measured. The greatest difference in the excitation spectra of healthy and infected seeds was observed in the short-wave range of 220–450 nm. At the same time, the excitation characteristics of infected seeds were higher than those of healthy ones, and the integral parameter Η in the entire range was 10–56% higher. A new maximum appeared at the wavelength of 232 nm and the maximum value increased by 362 nm. The spectral characteristics were measured when excited by radiation at wavelengths of 232, 362, 424, 485, 528 nm and the luminescence fluxes were calculated. It is established that the photoluminescence fluxes Φ in the short-wave ranges of 290–380 nm increase by 1.58–3.14 times and 390–550 nm-by 1.44–2.54 times. The fluxes in longer wavelength ranges do not change systematically and less significantly: for wheat, they decrease by 12% and increase by 19%, for barley, they decrease by 10% and increase by 33%. The flux decreases by 43–71% for oats. Based on the results obtained for cereal seeds, it is possible to further develop a method for detecting fusarium infection with absolute measurements of photoluminescence fluxes in the range of 290–380 nm, or when measuring photoluminescence ratios: for wheat seeds when excited with wavelengths of 424 nm and 232 nm (Φ_424_/Φ_232_); for barley seeds–when excited with wavelengths of 485 nm and 232 nm (Φ_485_/Φ_232_) and for oat seeds–when excited with wavelengths of 424 nm and 362 nm (Φ_424_/Φ_362_).

## 1. Introduction

*Fusarium* of the spike is the main disease of cereal plants worldwide. The disease can lead to a significant reduction in yield (up to 30%) and quality in the form of atrophy, weight loss and discoloration. *Fusarium* also produces mycotoxins, which can adversely affect livestock and human health [1]. Early and rapid detection, monitoring of the development of the disease are the bases for the control of *Fusarium* and the mycotoxins it produces in seeds. Currently, assessments are mainly carried out by human experts. Being subjective, such assessments may not always be consistent or completely reliable. Subjective methods for determining *Fusarium*, such as visual analysis, allow quickly obtain a qualitative result (yes / no), while objective methods provide quantitative data necessary to assess the phytotoxicity and variability of a pathogenic infection. Also, objective methods make it possible to determine virulence, aggressiveness, resistance to fungicides.

Methods have been investigated hyperspectral imaging as a basis for more reliable detection strategies [1, 2]. Previously, the possibilities of changes in the spectral reflectivity of wheat plant leaves during powdery mildew infection were investigated as a means of identifying and quantifying the severity of the disease and distinguishing between various diseases. Detection and recognition of diseases using reflection coefficient measurements can be implemented using certain wavelength ranges covering the visible and near infrared spectra (380–1300 nm) [3]. The difference in spectral reflection between healthy and diseased wheat plants infected with *Puccinia striiformis* (yellow rust) was studied [4]. The potential of continuous wavelet-analysis for detecting powdery mildew, banded rust and nitrogen-water stress in wheat was studied using hyperspectral data [5]. A convolutional neural network of differential amplification was proposed and used to identify images of wheat leaf diseases [6].

Hyperspectral images in the near-infrared region and their analysis were evaluated for their ability to track changes in fungal contamination and fungal activity directly under the surface of whole corn grains [7]. The difference in fluorescence emission between maize grains inoculated with toxigenic and atoxigenic inoculates of *A*. *Flavus* maize was evaluated using a fluorescent hyperspectral imaging system [8]_._

To date, photoluminescent diagnostic methods in ultraviolet and visible ranges have not been studied. They, like other optical methods, are highly accurate, selective, express, as well as distant and non-destructive, moreover, they have lower economic costs compared to previous methods. Their other advantages are the simplicity and safety of operation of devices for their implementation, a minimum of subjective factors and the possibility of integration into existing modern agricultural machines and units.

T-2 toxins are produced by various fungi of the *g*. *Fusarium* [9]. T-2 is the most toxic of the trichothecenes and it poses as a serious hazard to humans and animals if contaminated seeds and grain products are ingested. Unlike most biological toxins, T-2 mycotoxin can be absorbed through intact skin. Toxin-producing mold grows on a wide variety of cereals. Certain toxins produced by fungi of the *g*. *Fusarium* are characteristic of certain types of cereals, for example, the T-2 toxin often appears in oats [10]. Trichothecenes can cause severe skin problems or irritation of the intestinal mucosa and diarrhea. Suppression of the animal immune system has also been noted as a chronic effect of T-2 toxin infection.

The production of T-2 mycotoxin occurs when plants are infected with a fungus of the *g*. *Fusarium*, followed by deep penetration of the toxin into the seeds [11]. Importance in the identification of T-2 mycotoxin is the widespread production of foodstuffs from grains that are potentially susceptible to *Fusarium spp*. infection such as barley, wheat and oats [12]. Nowdays, it is used such method as immunochemical and chromatographic (gas chromatography, thin layer chromatography, high performance liquid chromatography) methods [13] and various methods of nuclear magnetic resonance [11] and X-ray diffraction spectroscopy for the identification and study of the structure of the T-2 toxin [12].

The purpose of this study is to identify the possibilities of diagnosing fusariosis in cereal seeds by the photoluminescence method in the near ultraviolet and visible range.

## 2. Materials and methods

We studied seeds infected with one of the most common and dangerous diseases for plants–fusariosis, the causative agents of which are fungi of the genus Fusarium. Seeds of winter wheat, barley and oats were used as experimental materials.

The selection of infected seeds was made from a model experiment on the second day after harvesting the seeds. The degree of infection of a batch of seeds from the harvest of 2021 with *Fusarium* was carried out according to the method "Method for determining the content of Fusarium grains" (Interstate standard GOST 31646–2012). Clearly *Fusarium infected* seeds are isolated from the sample by manual disassembly, determined by a complex of external features: the shape of the grain (most grains are wrinkled, feeble. They have pointed barrels and a strongly depressed groove, with late damage. The seeds can be swollen with a peeling, crumbling shell); the appearance and characteristics of the surface of the grain (the grain is whitish, chalky, spots and pink bloom may be present on the surface, complete loss of gloss, crumbling, peeling shells); structure of the endosperm (significant or complete loss of vitreousness, the endosperm is friable, crumbling, with a mealy consistency), on the germinal part and in the groove there is a light felt coating of the fungus, which has a light gray or light pink hue, the embryo on the cut is dark in color (gray, brown, fulvous).

Additionally, the method of thin-layer chromatography was used to detect the T-2 toxin by excitation of fluorescence with 365 nm radiation after treatment with an alcoholic solution of sulfuric acid, followed by heating at 100–105°C.

The luminescence was studied using the “Fluorat-02-Panorama” spectrofluorimeter with the “Panorama Pro” software installed. The excitation and photoluminescence spectra were measured in the same way as previously performed measurements [14]_._

The excitation spectra η_e_(λ) were measured during synchronous scanning and the photoluminescence spectra φ_*l*_(λ) were based on them. According to the measurement results, statistical processing was carried out, where averaging was carried out over 250 spectra. The integral parameters of the spectra were calculated in the "Panorama Pro" program: H–integral absorption capacity and Ф–photoluminescence flux expressed in relative units.

$$H=\int_{\lambda_{1}}^{\lambda_{2}}\eta_{e}(\lambda )d\lambda ,$$ (1)

η_e_(λ)–spectral characteristics of the excitation λ_1_…λ_2_ –limits of the operating spectral range of excitation.

$$Ф=\int_{\lambda_{1}}^{\lambda_{2}}\varphi_{l}(\lambda )d\lambda ,$$ (2)

φ_*l*_(λ)–spectral characteristics of photoluminescence, λ_1_…λ_2_ –limits of the operating spectral range of photoluminescence

## 3. Results and discussion

**Fig 1:**
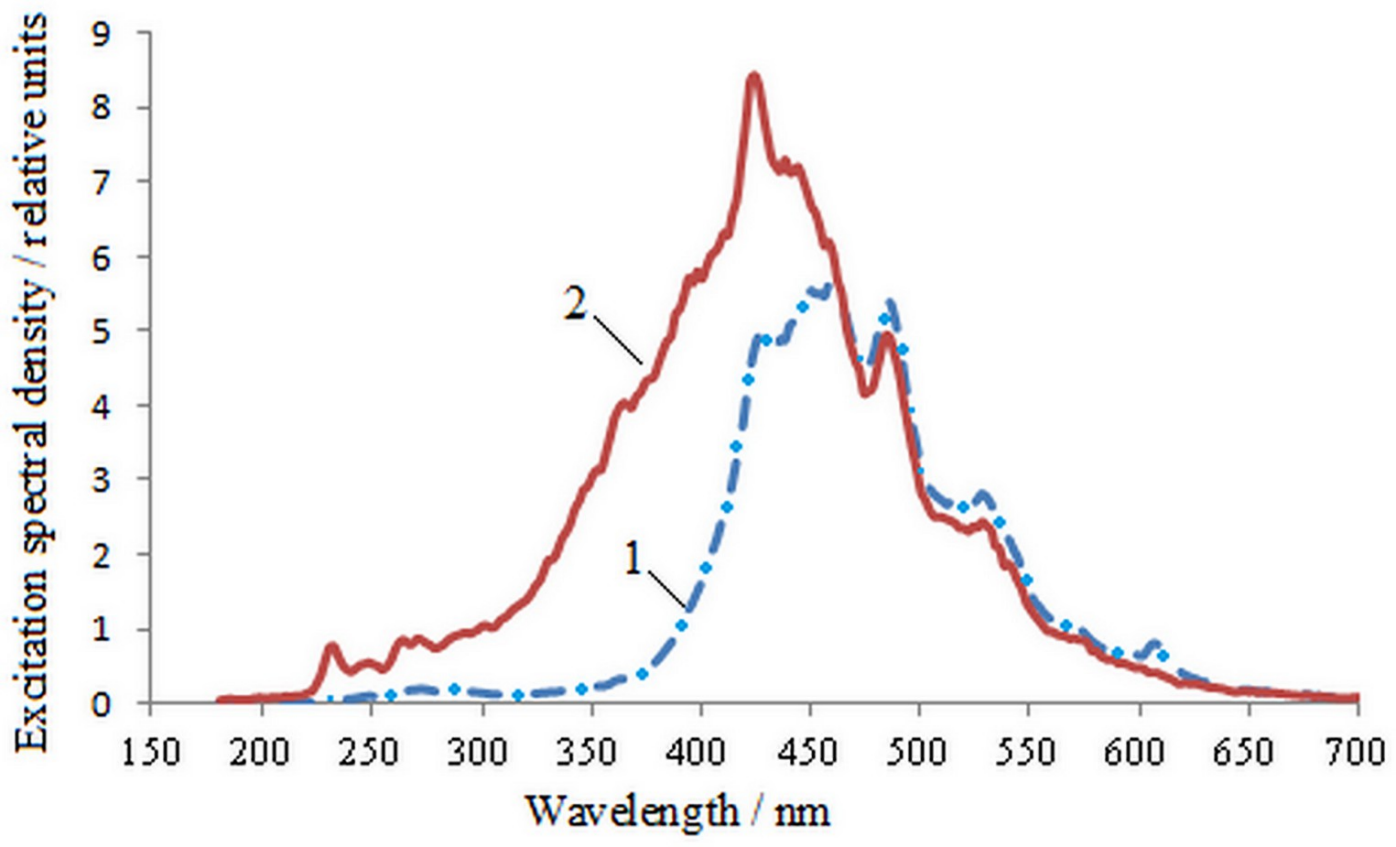
Excitation spectra of barley seeds during synchronous scanning. 1—uninfected, 2—infected on 98%.

250 measurements were carried out with simultaneous scanning of infected and uninfected seeds. The average results of measurements of barley seeds are shown in Fig 1.

It follows from Fig 1 that during infection, seeds appear at a maximum at a wavelength of 232 nm, and there is also a quantitative increase in peaks at wavelengths: 362 nm and 424 nm. The spectrum shifts to the left. The quantitative values of η_e_ in the maxima at 485nm and 528 nm in healthy seeds slightly exceed the similar values for infected seeds. The previously obtained changes in the dependence of the spectral characteristics of seeds during maturation [14] allow us to conclude that the excitation spectrum of infected seeds is close to the excitation spectrum of immature seeds in the wavelength range shorter than 400 nm. It can be assumed that *Fusarium* infection slows down maturation.

The integral parameters of the excitation spectra of seeds of various degrees of fusarium infection in different spectral ranges corresponding to the maxima are presented in Table 1.

**Table 1:** Integral parameters of excitation spectra of plant seeds of various degrees of fusarium infection.

Degree of infection with fusarium, %	H, r.u.	H, r. u. (for spectral range, nm)				
		220–240	340–400	400–460	460–500	500–590
Wheat						
0	765	2	199	291	116	83
98	938	6	250	294	138	119
Barley						
0	748	1	36	276	205	179
98	1164	9	256	434	187	151
Oats						
0	1507	2	197	647	346	238
98	1657	5	439	717	223	161

Thus, in infected seeds, the integral absorption capacity Н in the range of 220–240 nm (peak 232 nm) exceeds the same indicator for healthy seeds by 2.5–9.0 times, in the range of 340–400 nm (peak 362 nm) by 1.26–7.11 times. In the range of 400–460 nm (peak 424 nm), this ratio is lower-1.01–1.57 times. In the longer wavelength ranges of 460–500 nm (peak 485 nm) and 510–550 nm (peak 528 nm), the excess of the integral absorption capacity of infected seeds over healthy ones is observed only for wheat seeds–by 1.19 and 1.43 times, respectively. The opposite is true for barley and oat seeds: the absorption capacity of healthy seeds is 1.09 and 1.19 times higher for barley and 1.55 and 1.48 times higher for oats.

**Fig 2:**
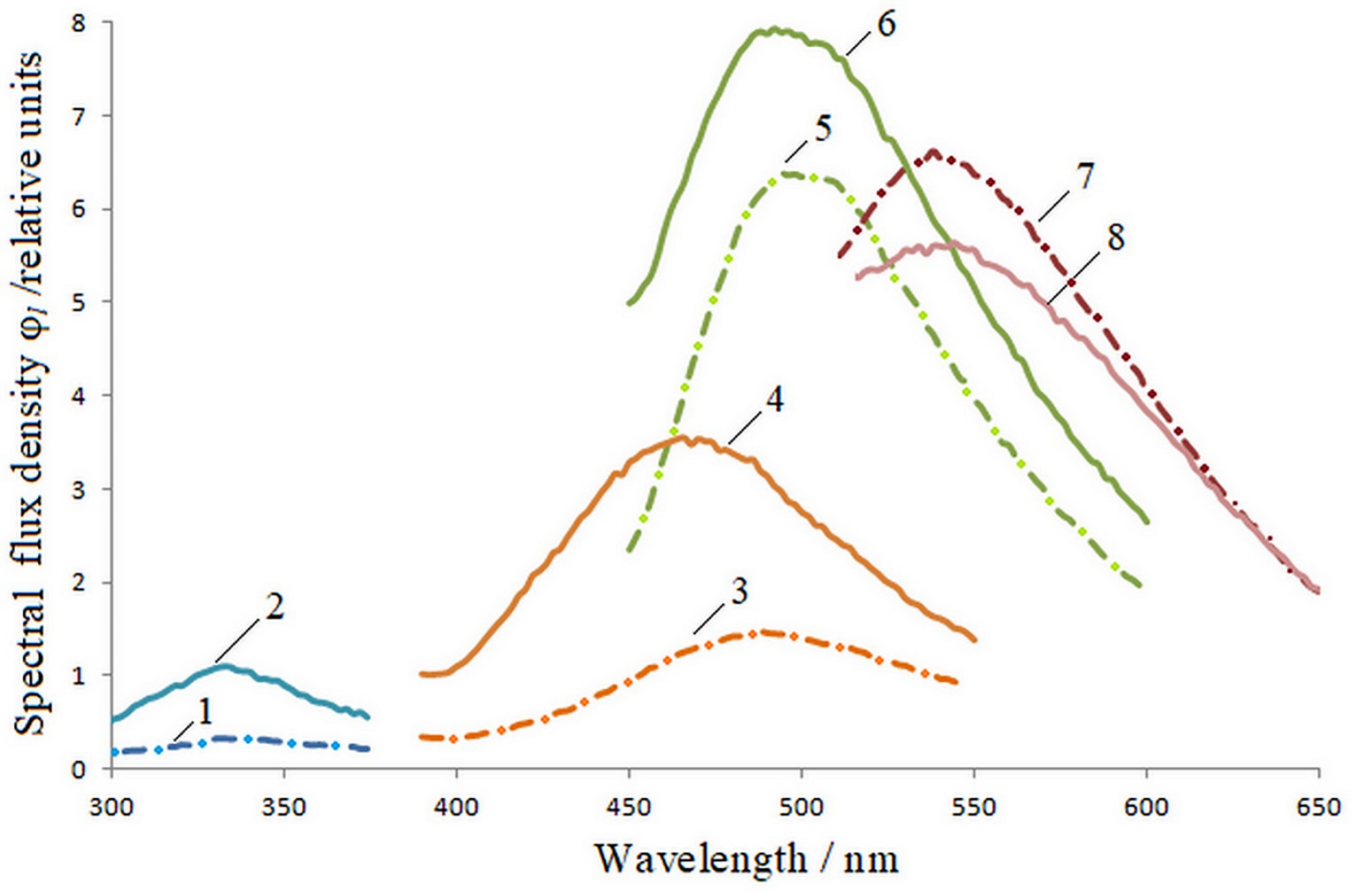
Luminescence spectra of barley seeds when excited by radiation. λ_e_ = 232 nm: 1 –uninfected, 2 –infected; λ_e_ = 362 nm: 3– uninfected, 4 –infected; λ_e_ = 424 nm: 5 –uninfected, 6 –infected; λ_e_ = 485nm: 7 –uninfected, 8 –infected.

Knowing the wavelengths of the excitation maxima λ_e_, we measure the luminescence spectra φ_*l*_(λ). Fig 2 shows the spectral characteristics of the luminescence of barley seeds at different λ_e_.

The radiation fluxes calculated by the formula (2) are presented in Table 2.

**Table 2:** Integral parameters of luminescence spectra of plant seeds of various degrees of fusarium infection.

Degree of infection with fusarium, %	Ф_232_, r.u.	Ф_362_, r.u.	Ф_424_, r.u.	Ф_485_, r.u.	Ф_528_, r.u.
Wheat					
0	36	387	833	501	222
98	95	556	746	545	264
Barley					
0	21	152	653	694	-
98	66	386	870	633	257
Oats					
0	31	349	1383	1201	528
98	49	677	809	809	369

The ratio of fluxes for infected wheat seeds exceeds similar indicators for healthy seeds by 1.09–2.63 times, except for the excitation of λ_e_ = 424 nm, where it is 12% less. For barley seeds, the luminescence fluxes of infected seeds are higher at the excitation wavelengths of 232 nm–by 3.14 times, 362 nm-by 2.54 times and 424 nm-by 1.33 times, but at the wavelength of 485nm–decreases by 1.10 times. Photoluminescence fluxes of infected oat seeds exceed the fluxes of healthy seeds when excited with a wavelength of 232 nm-by 1.58 times and 362 nm-by 1.94 times. For other excitation wavelengths, on the contrary, the photoluminescence fluxes of healthy seeds are larger: at λ_e_ = 424 nm-by 1.71 times, λ_e_ = 485 nm-by 1.48 times and λ_e_ = 528 nm-by 1.43 times.

Thus, the detection of fusarium-infected seeds is possible with absolute photoluminescence measurements in the range of 290–380 nm, or with the measurement of photoluminescence ratios: Ф_424_/Ф_232_ for wheat seeds; Ф_485_/Ф_232_ for barley seeds and Ф_424_/Ф_362_ for oats seeds.

A significant difference in fluxes Φ_232_ suggests the use of seed fluorescence in the spectral range 290–380 nm (with excitation λ_e_ = 232 nm) to determine the proportion of infected seeds in the sample. The disadvantage of such absolute measurements is the relatively small value of the initial photosignal (6–23 times for wheat, 6–33 times for barley, and 11–45 times for oats) and its dependence on external factors associated with both the seeds themselves and with measuring device. Preliminary calibration of the device is necessary, which allows moving from the photosignal to the percentage of seed infection.

Another option for detecting infected seeds in a sample is the introduction of infection markers by the ratio of photoluminescence fluxes when excited by radiation of certain wavelengths. For wheat seeds, the most suitable ratio is Ф_424_/Ф_232_; for barley seeds—Ф _485_/ Ф _232_ and for oat seeds—Ф _424_/ Ф _362_.

Such relative measurements make it possible not to take into account the absolute values of flukes, as well as changes in individual external factors (temperature, surface condition). The disadvantage of the method is the need to use two radiation sources and receivers in the measuring device.

The studied method makes it possible to objectively determine the infection of seeds of cereal plants with *Fusarium* even in cases where visual differences are not noticeable, for example, in oat seeds.

The photoluminescent method makes it possible to establish the fact of *Fusarium* infection of an individual seed by the ratio of luminescence fluxes at certain excitation wavelengths. To diagnose infection with other pathogenic microorganisms, it is necessary to conduct similar studies to study their effect on the optical properties of seeds. To analyze the infection of a large number of seeds, it is necessary to measure the photoluminescence signal integrated over the surface of these seeds, by analyzing which the proportion of infected seeds is determined.

Sensor applications for automated, objective and reproducible assessment of plant diseases using optical sensors will prevail over the time-consuming visual assessment of technicians. In the future, additional areas of research need to be linked, such as plant pathology, sensor development, computer science, and machine learning. Only an interdisciplinary approach with close links to practical agriculture can lead to powerful solutions for diagnosing and detecting diseases with high accuracy and sensitivity [15].

## 4. Conclusion

The greatest difference in the excitation spectra of healthy and infected seeds was observed in the short-wave range of 220–450 nm. At the same time, the characteristics of η_e_(λ) of infected seeds were higher than those of healthy ones, and the integral parameter Н in the entire range was 10–56% more. A new maximum appears at the wavelength of 232 nm and increases the maximum by 362 nm. As a result, photoluminescence fluxes increase in the short-wave ranges of 290–380 nm and 390–550 nm. The flows in the longer wavelength ranges do not change systematically.

Based on the results obtained for cereal seeds, it is possible to further develop a method for determining the degree of fusarium infection with absolute photoluminescence measurements in the range of 290-380nm, or when measuring photoluminescence ratios: Ф_424_/Ф_232_ for wheat seeds; Ф_485_/Ф_232_ for barley seeds and Ф_424_/Ф_362_ for oats seeds.

## Supporting information

S1 Data (ZIP)
