# Supplementary figures and images for: Detection of Fusarium infected seeds of cereal plants by the fluorescence method (part 2 of 2)
Source: PLoS One. 2022 Jul 1;17(7):e0267912. doi: 10.1371/journal.pone.0267912 (PMC9261490; doi:10.1371/journal.pone.0267912)

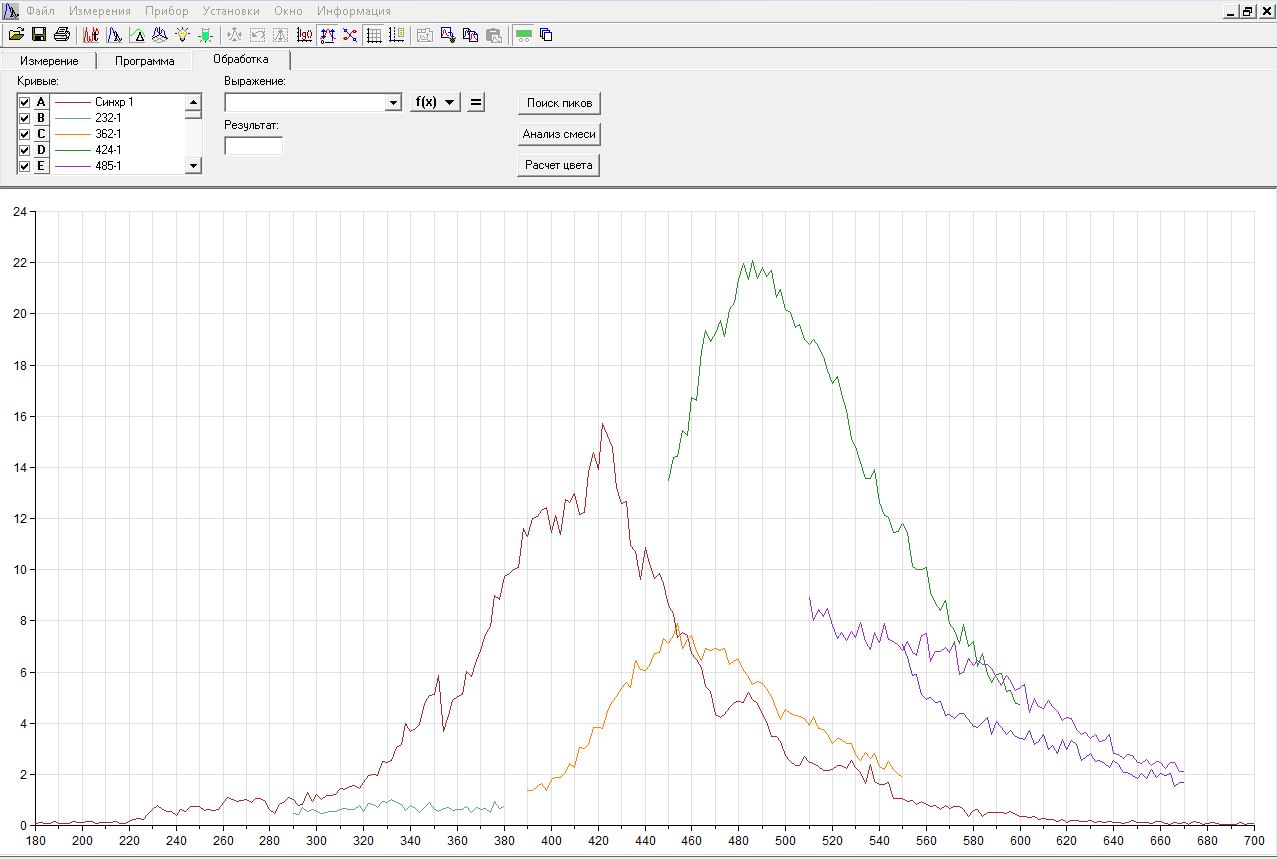

Supplement: S1 Data — (ZIP) [file pone.0267912.s001.zip › Primary Data/Oats/Infected/æ»Ñ¬Γαδ «óßá oáαáaÑ¡¡«ú« 1.JPG]

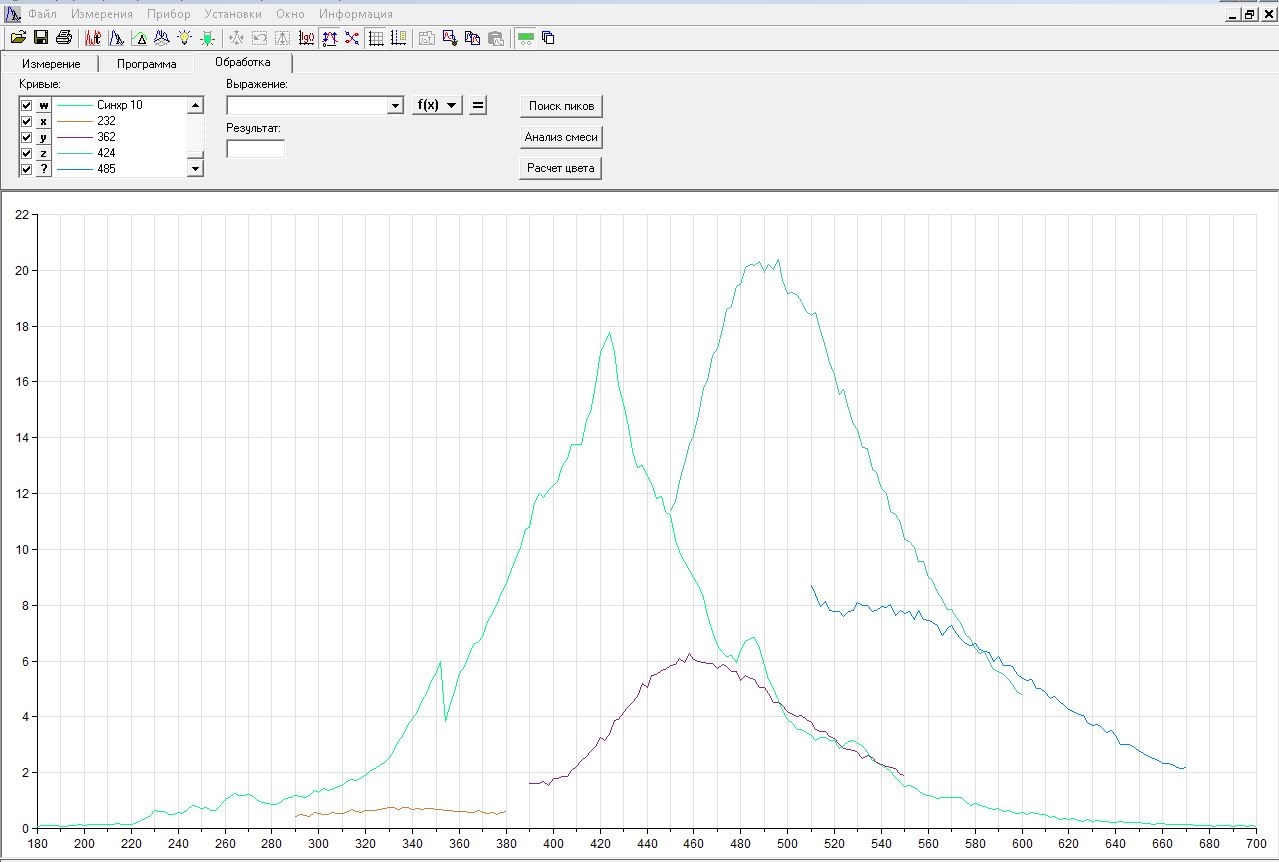

Supplement: S1 Data — (ZIP) [file pone.0267912.s001.zip › Primary Data/Oats/Infected/æ»Ñ¬Γαδ «óßá oáαáaÑ¡¡«ú« 10.JPG]

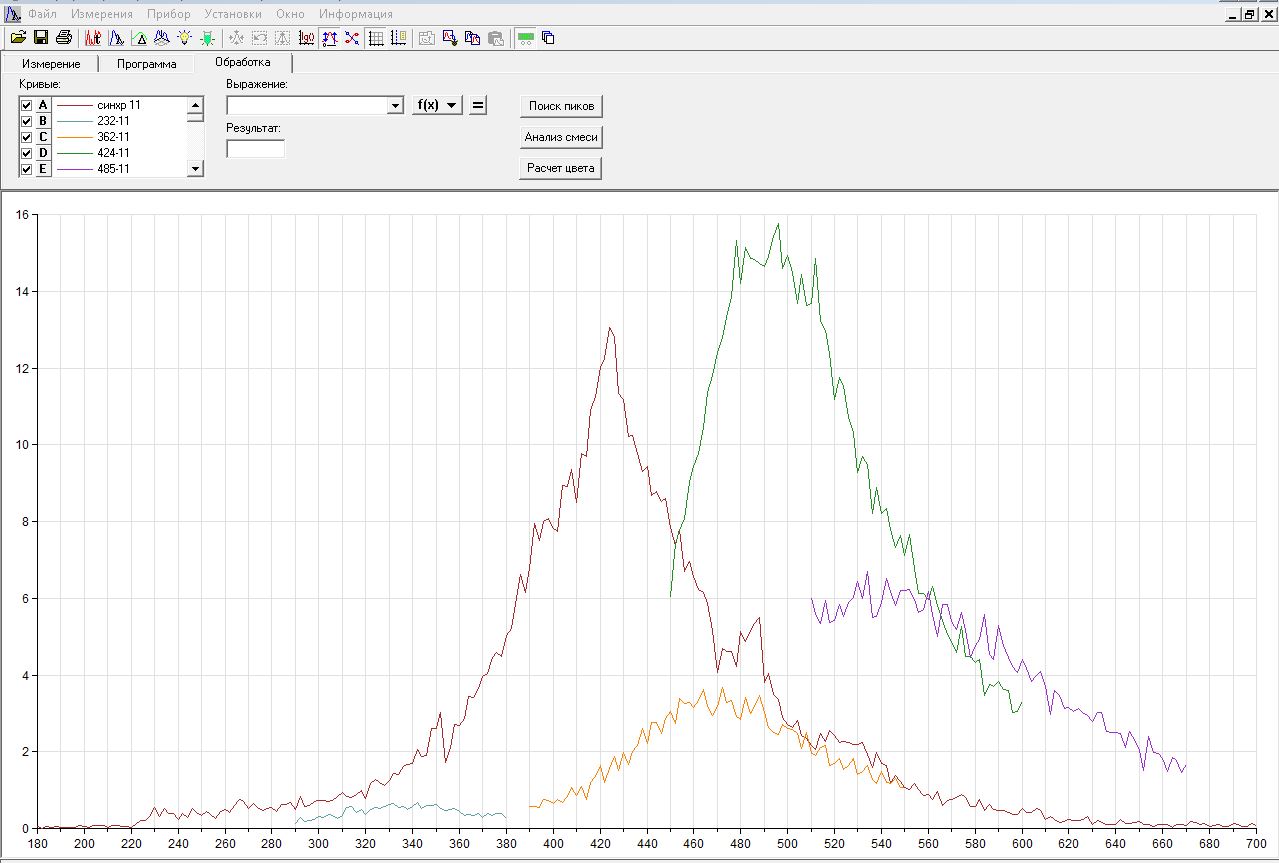

Supplement: S1 Data — (ZIP) [file pone.0267912.s001.zip › Primary Data/Oats/Infected/æ»Ñ¬Γαδ «óßá oáαáaÑ¡¡«ú« 11.JPG]

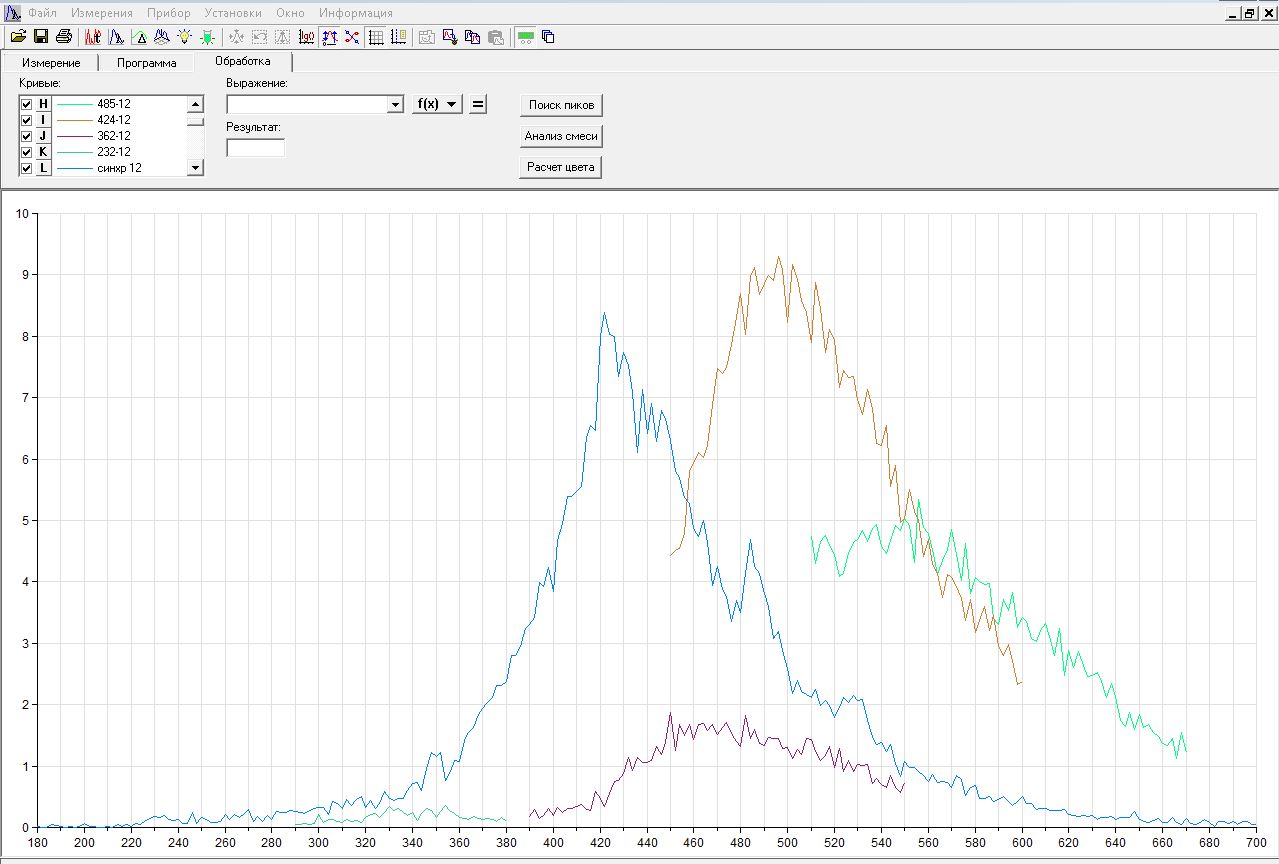

Supplement: S1 Data — (ZIP) [file pone.0267912.s001.zip › Primary Data/Oats/Infected/æ»Ñ¬Γαδ «óßá oáαáaÑ¡¡«ú« 12.JPG]

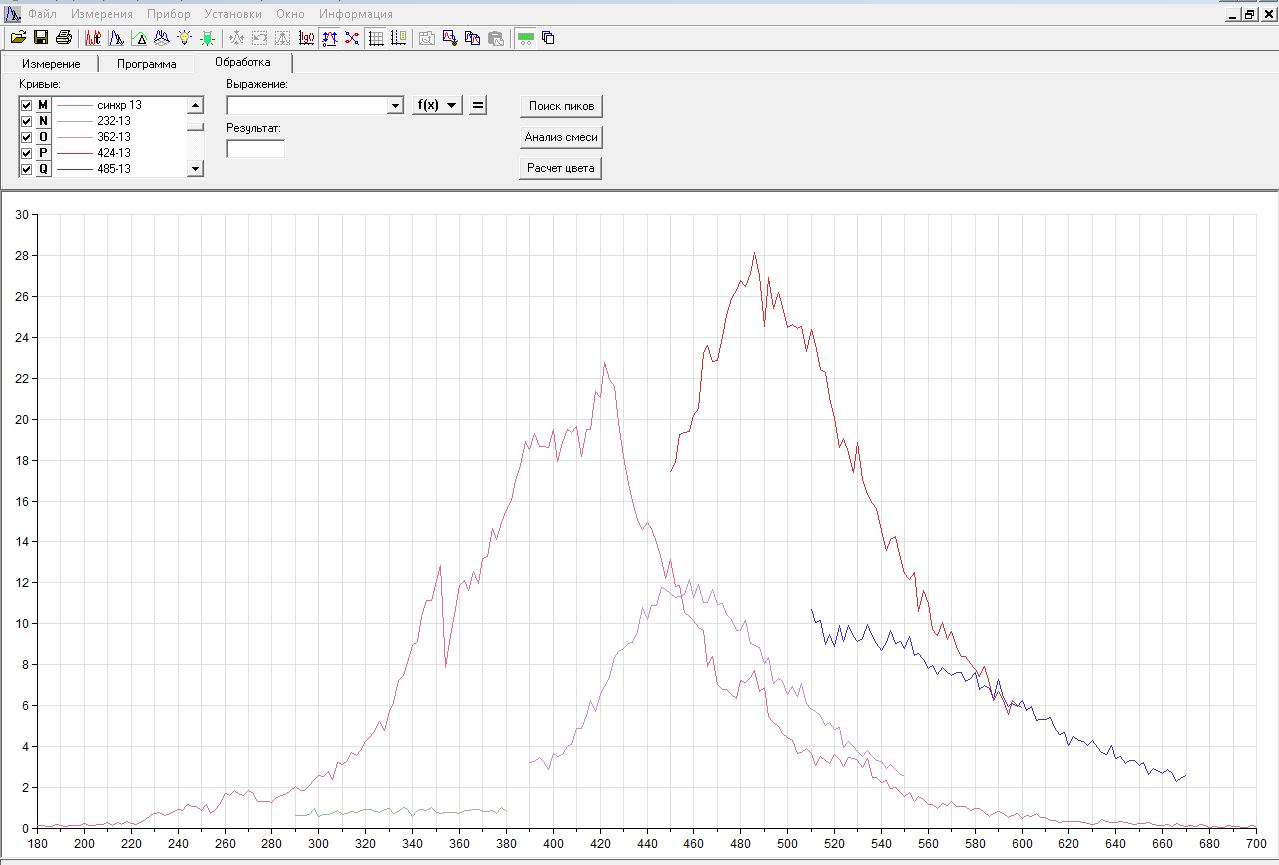

Supplement: S1 Data — (ZIP) [file pone.0267912.s001.zip › Primary Data/Oats/Infected/æ»Ñ¬Γαδ «óßá oáαáaÑ¡¡«ú« 13.JPG]

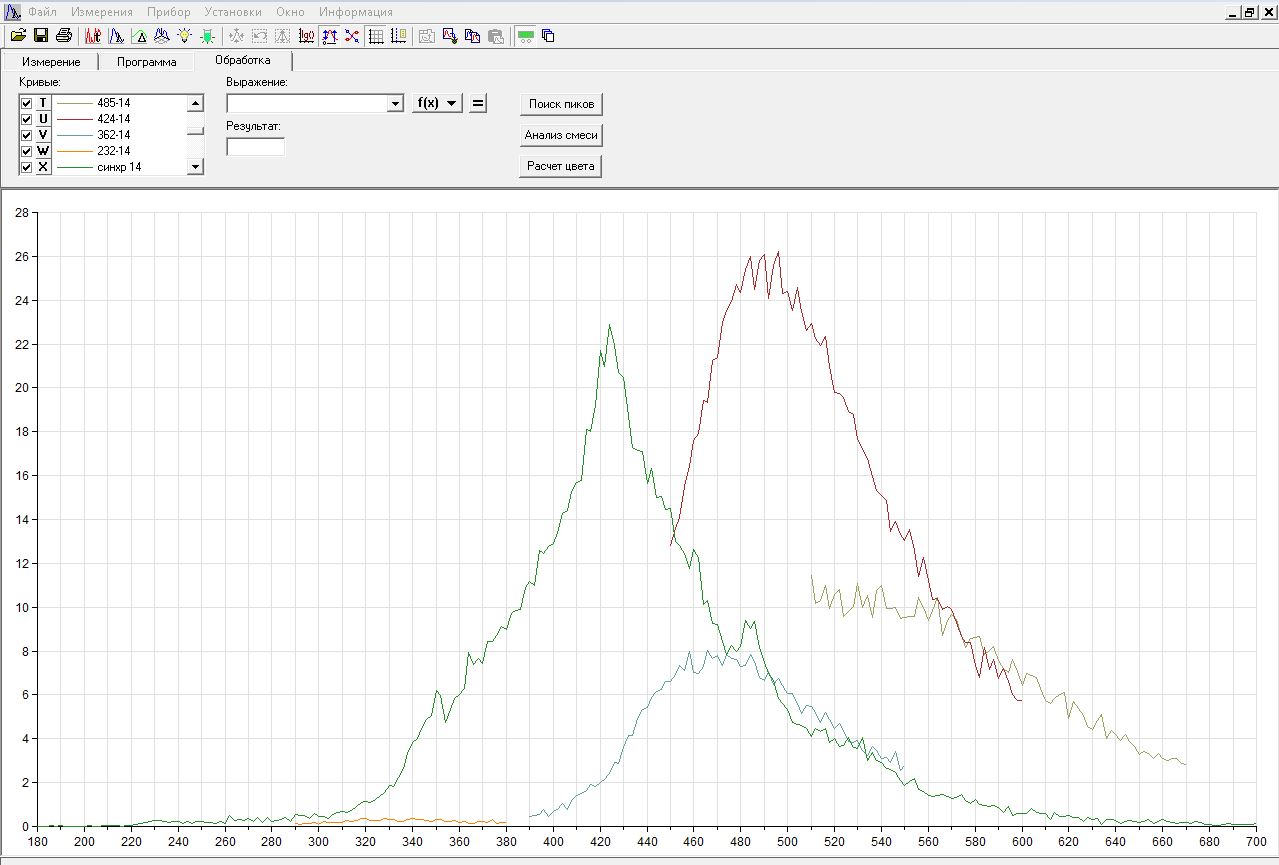

Supplement: S1 Data — (ZIP) [file pone.0267912.s001.zip › Primary Data/Oats/Infected/æ»Ñ¬Γαδ «óßá oáαáaÑ¡¡«ú« 14.JPG]

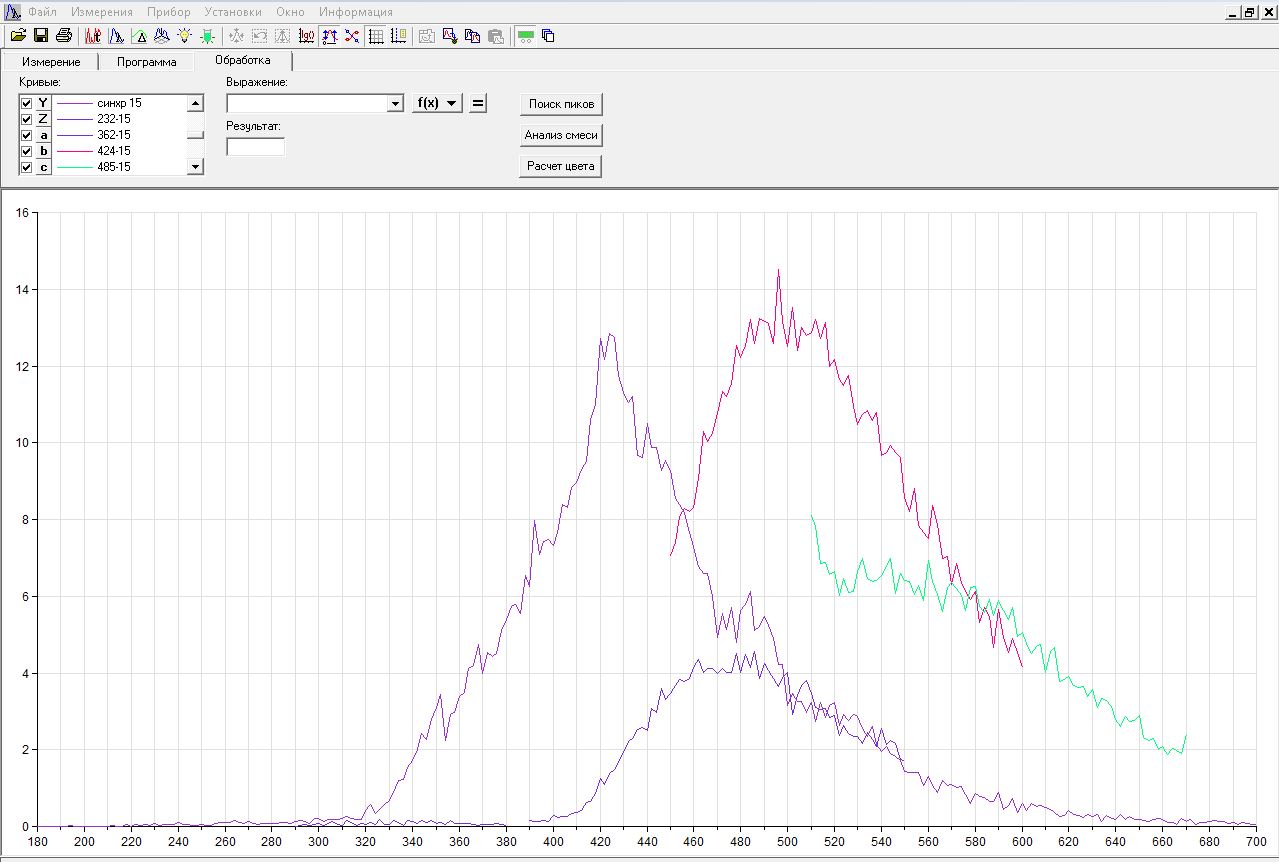

Supplement: S1 Data — (ZIP) [file pone.0267912.s001.zip › Primary Data/Oats/Infected/æ»Ñ¬Γαδ «óßá oáαáaÑ¡¡«ú« 15.JPG]

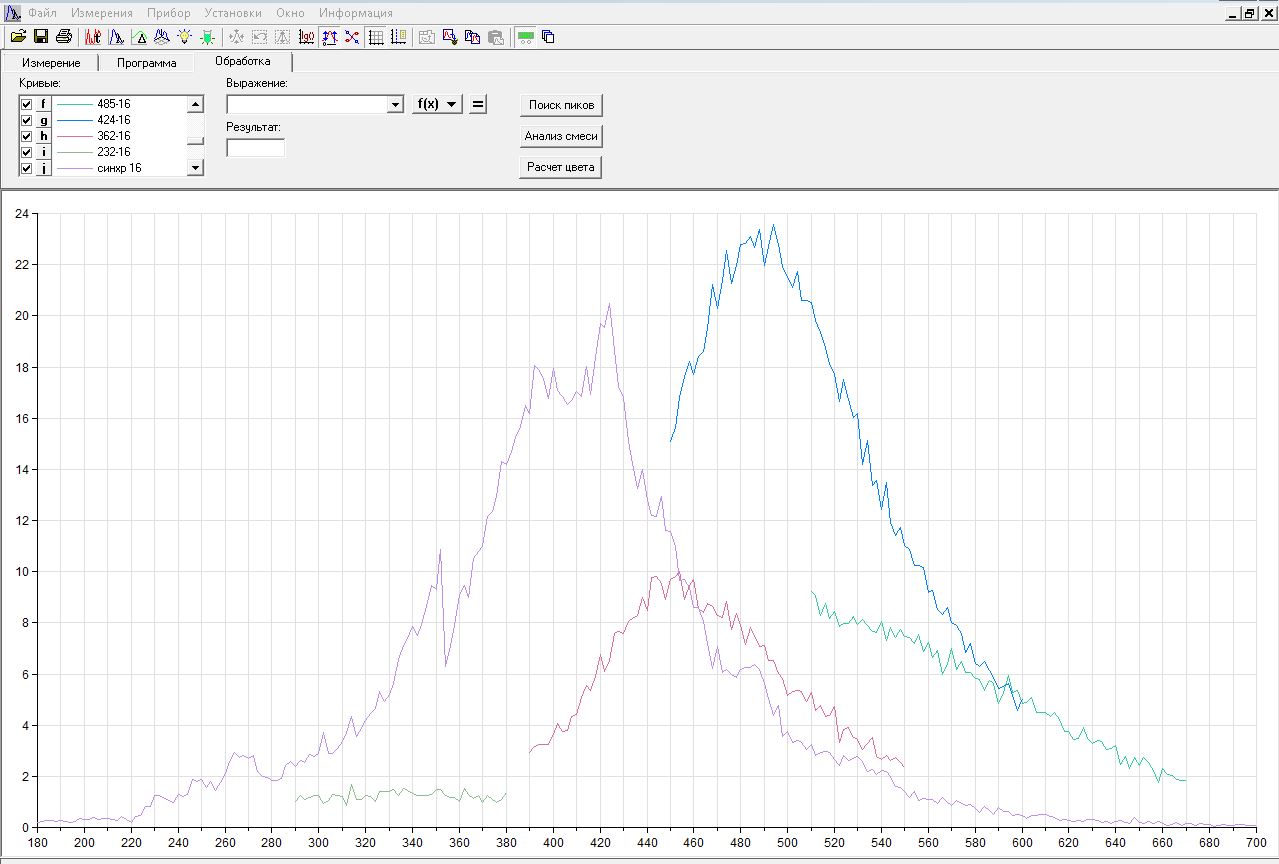

Supplement: S1 Data — (ZIP) [file pone.0267912.s001.zip › Primary Data/Oats/Infected/æ»Ñ¬Γαδ «óßá oáαáaÑ¡¡«ú« 16.JPG]

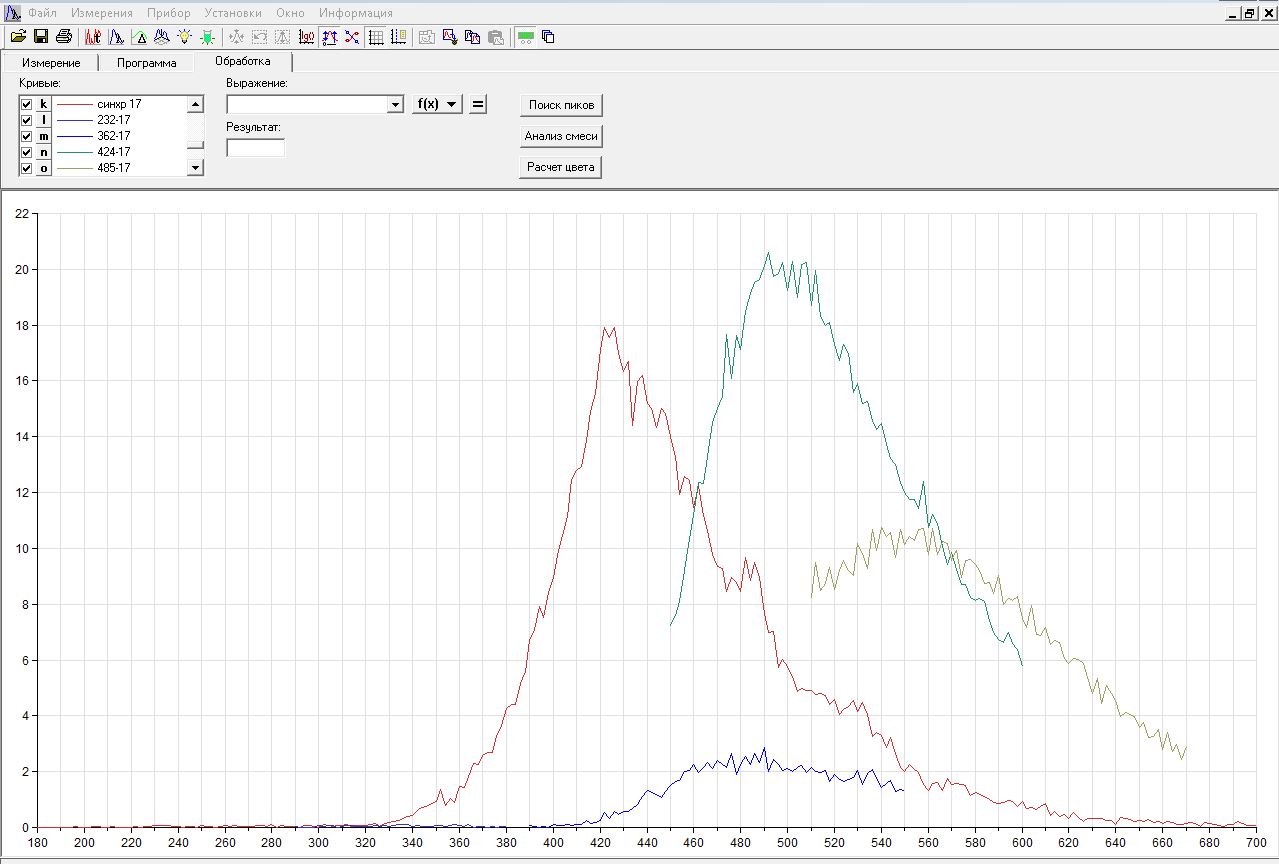

Supplement: S1 Data — (ZIP) [file pone.0267912.s001.zip › Primary Data/Oats/Infected/æ»Ñ¬Γαδ «óßá oáαáaÑ¡¡«ú« 17.JPG]

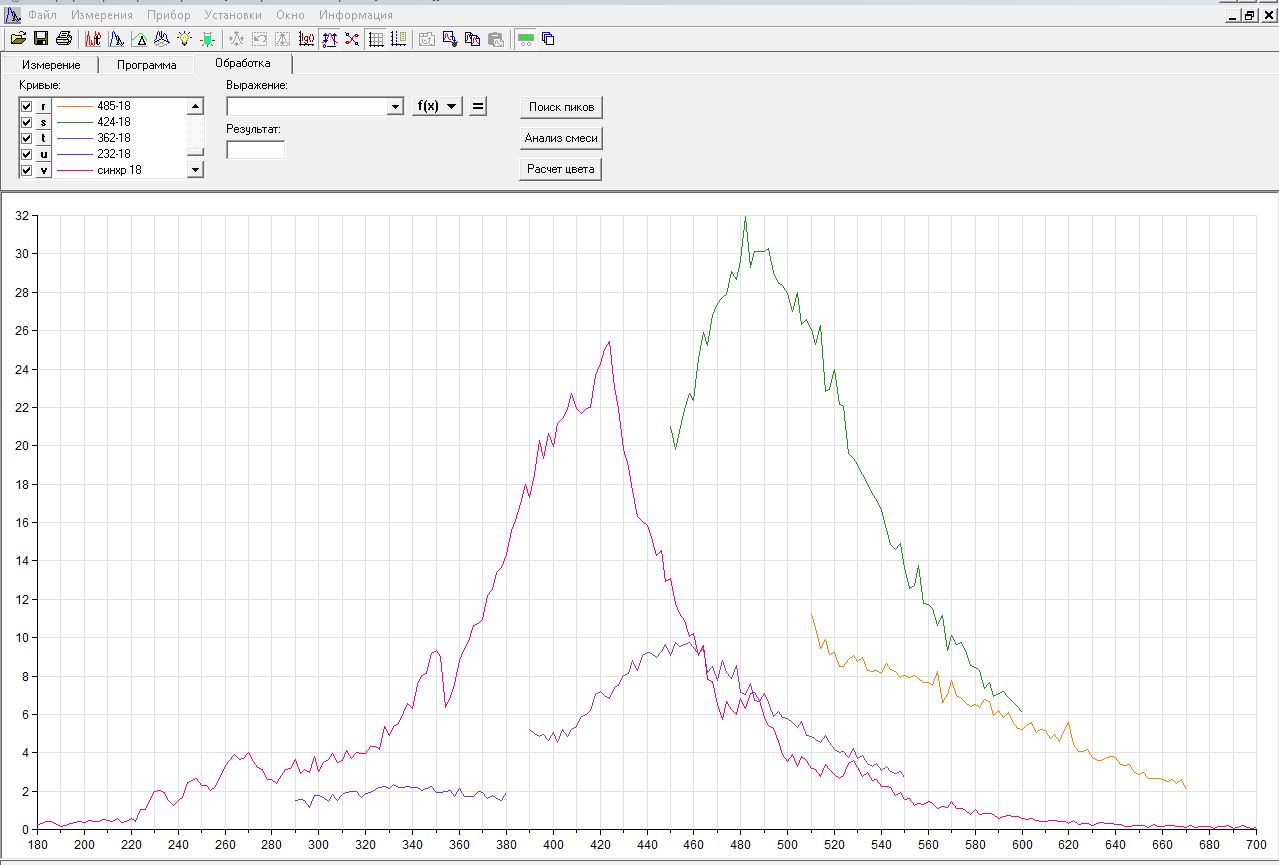

Supplement: S1 Data — (ZIP) [file pone.0267912.s001.zip › Primary Data/Oats/Infected/æ»Ñ¬Γαδ «óßá oáαáaÑ¡¡«ú« 18.JPG]

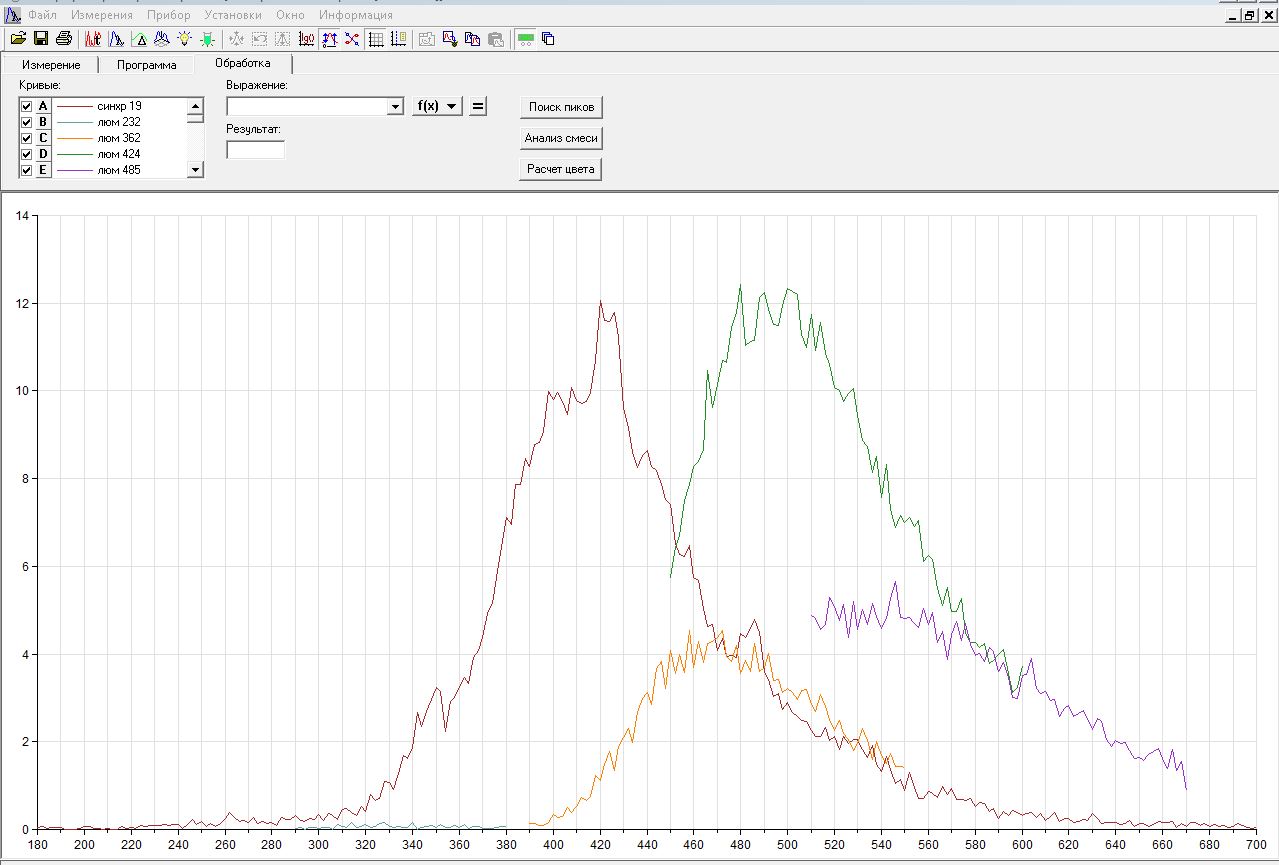

Supplement: S1 Data — (ZIP) [file pone.0267912.s001.zip › Primary Data/Oats/Infected/æ»Ñ¬Γαδ «óßá oáαáaÑ¡¡«ú« 19.JPG]

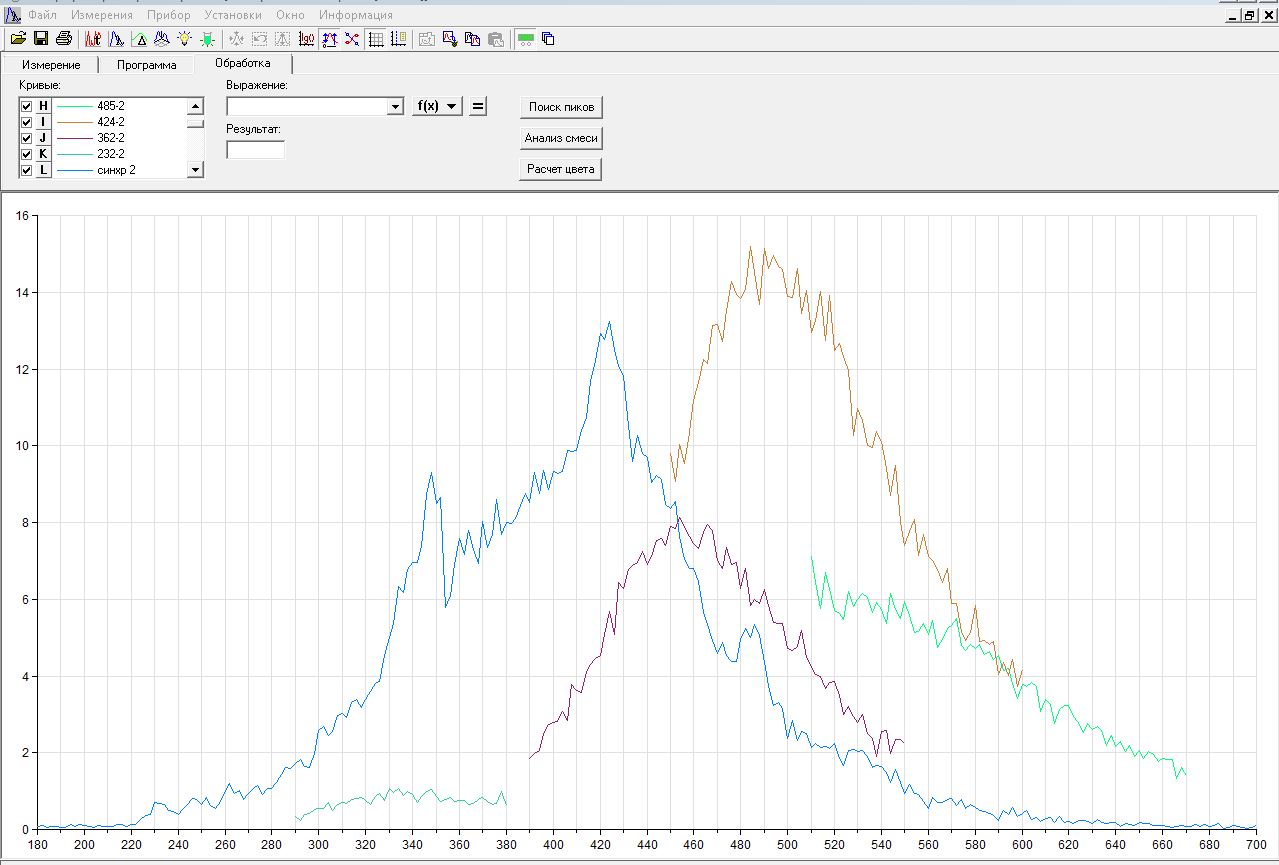

Supplement: S1 Data — (ZIP) [file pone.0267912.s001.zip › Primary Data/Oats/Infected/æ»Ñ¬Γαδ «óßá oáαáaÑ¡¡«ú« 2.JPG]

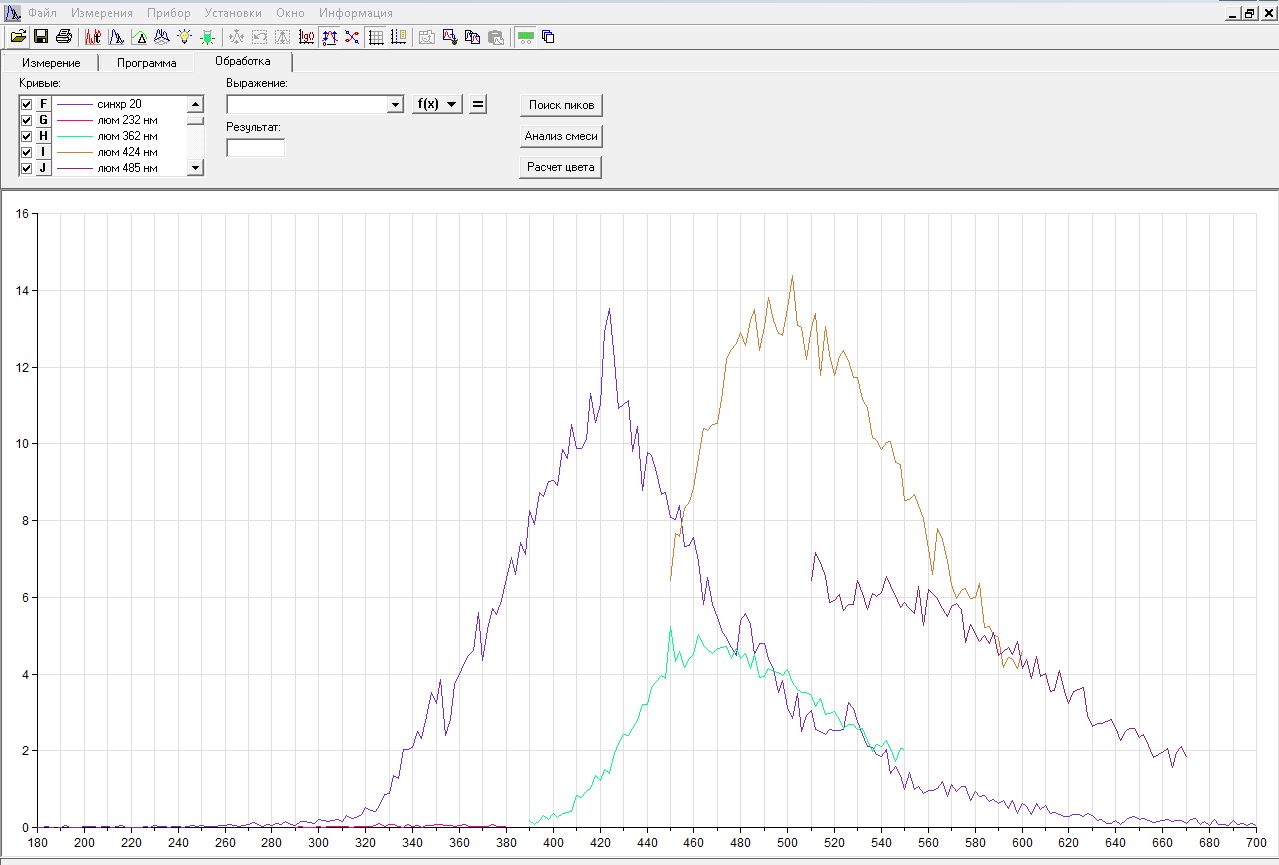

Supplement: S1 Data — (ZIP) [file pone.0267912.s001.zip › Primary Data/Oats/Infected/æ»Ñ¬Γαδ «óßá oáαáaÑ¡¡«ú« 20.JPG]

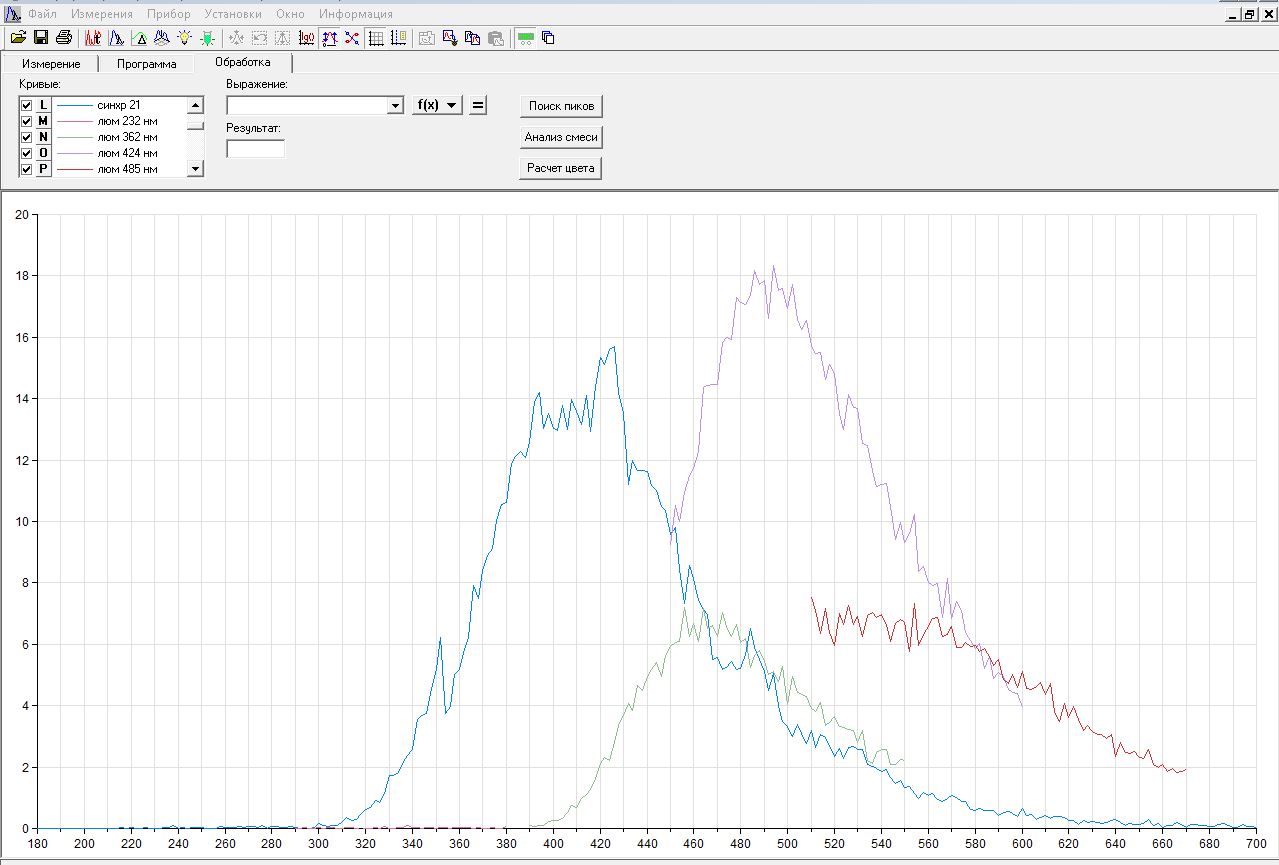

Supplement: S1 Data — (ZIP) [file pone.0267912.s001.zip › Primary Data/Oats/Infected/æ»Ñ¬Γαδ «óßá oáαáaÑ¡¡«ú« 21.JPG]

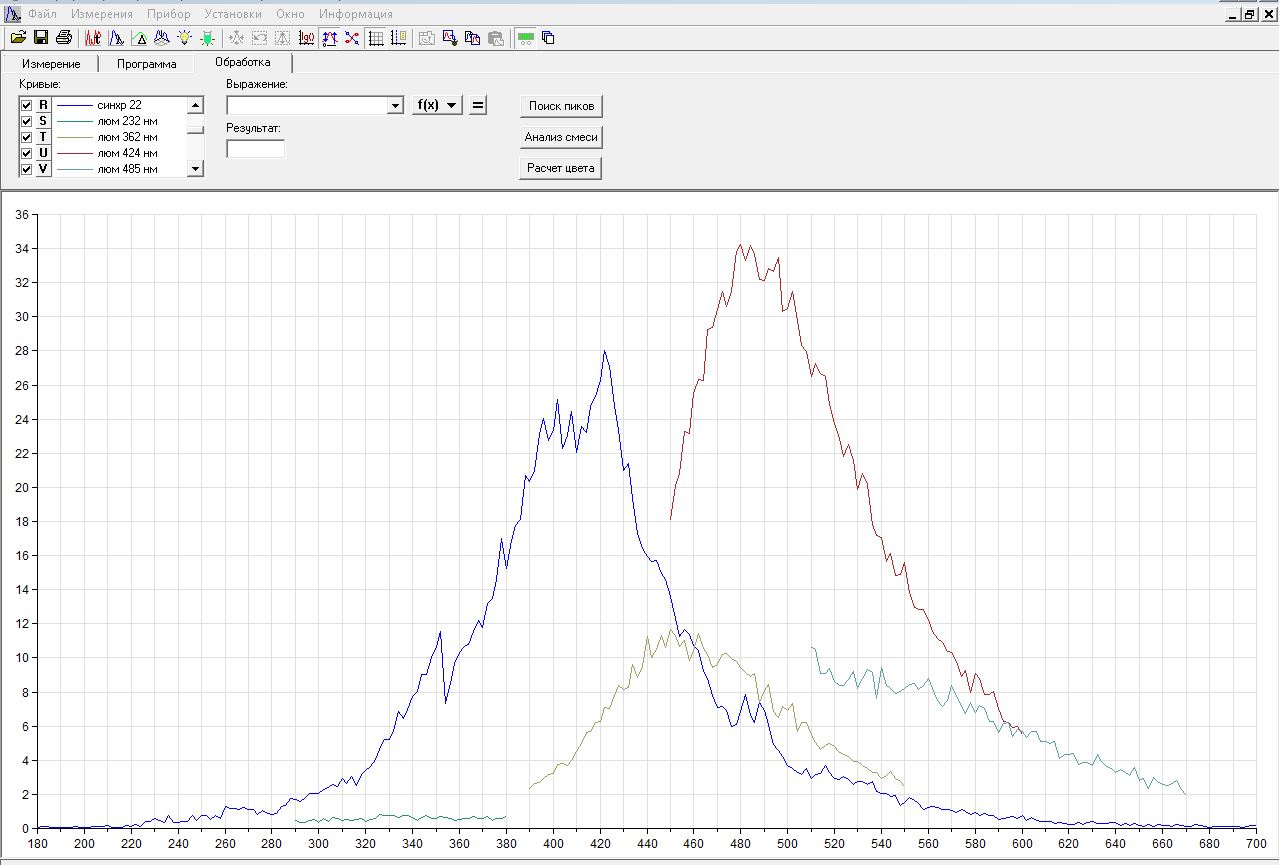

Supplement: S1 Data — (ZIP) [file pone.0267912.s001.zip › Primary Data/Oats/Infected/æ»Ñ¬Γαδ «óßá oáαáaÑ¡¡«ú« 22.JPG]

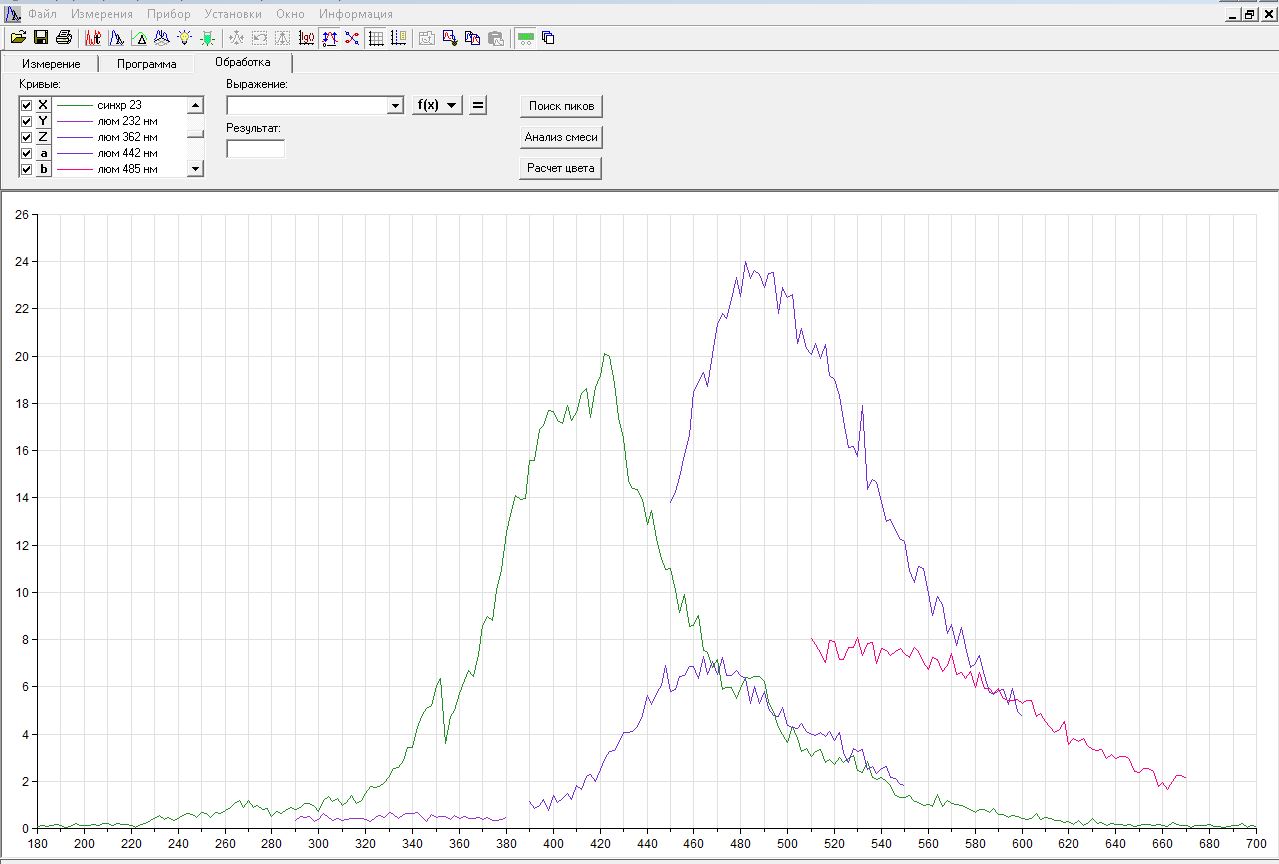

Supplement: S1 Data — (ZIP) [file pone.0267912.s001.zip › Primary Data/Oats/Infected/æ»Ñ¬Γαδ «óßá oáαáaÑ¡¡«ú« 23.JPG]

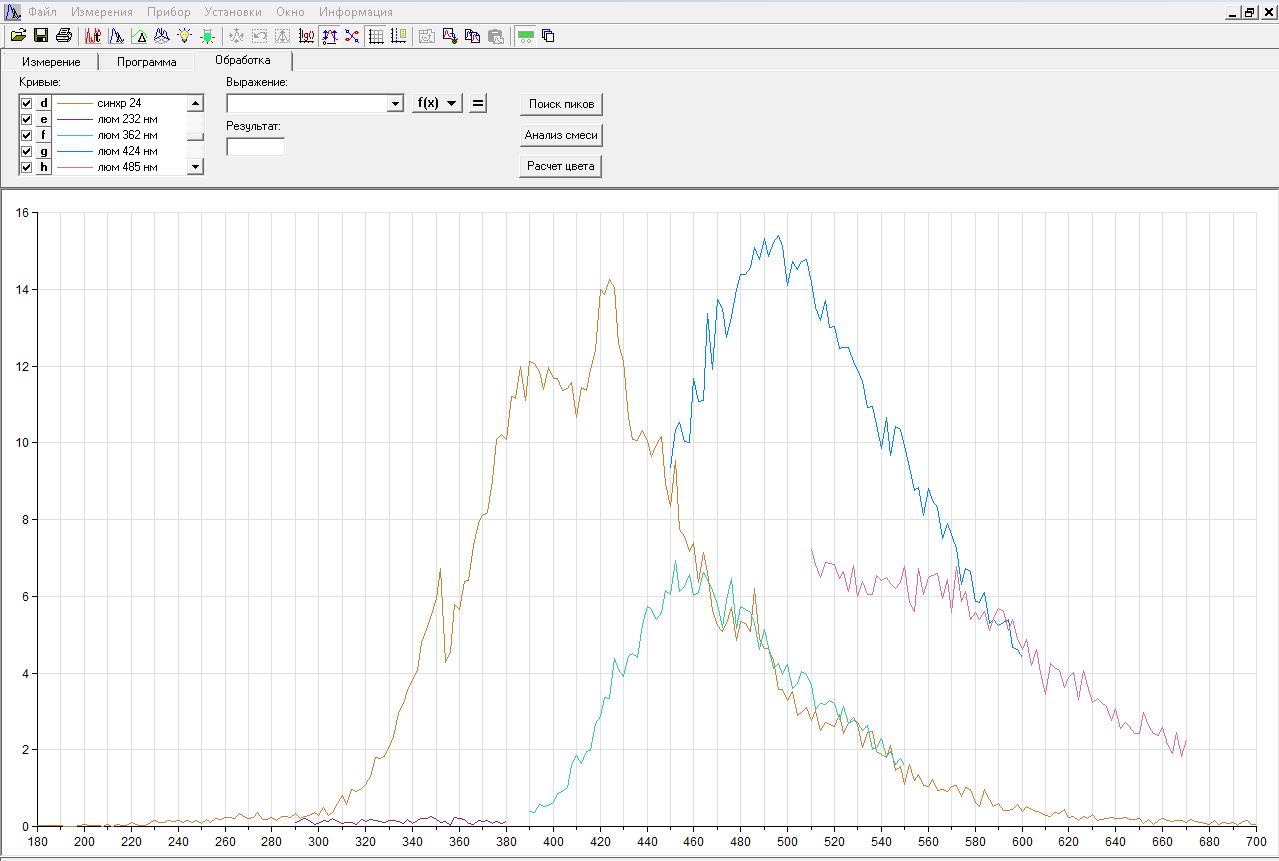

Supplement: S1 Data — (ZIP) [file pone.0267912.s001.zip › Primary Data/Oats/Infected/æ»Ñ¬Γαδ «óßá oáαáaÑ¡¡«ú« 24.JPG]

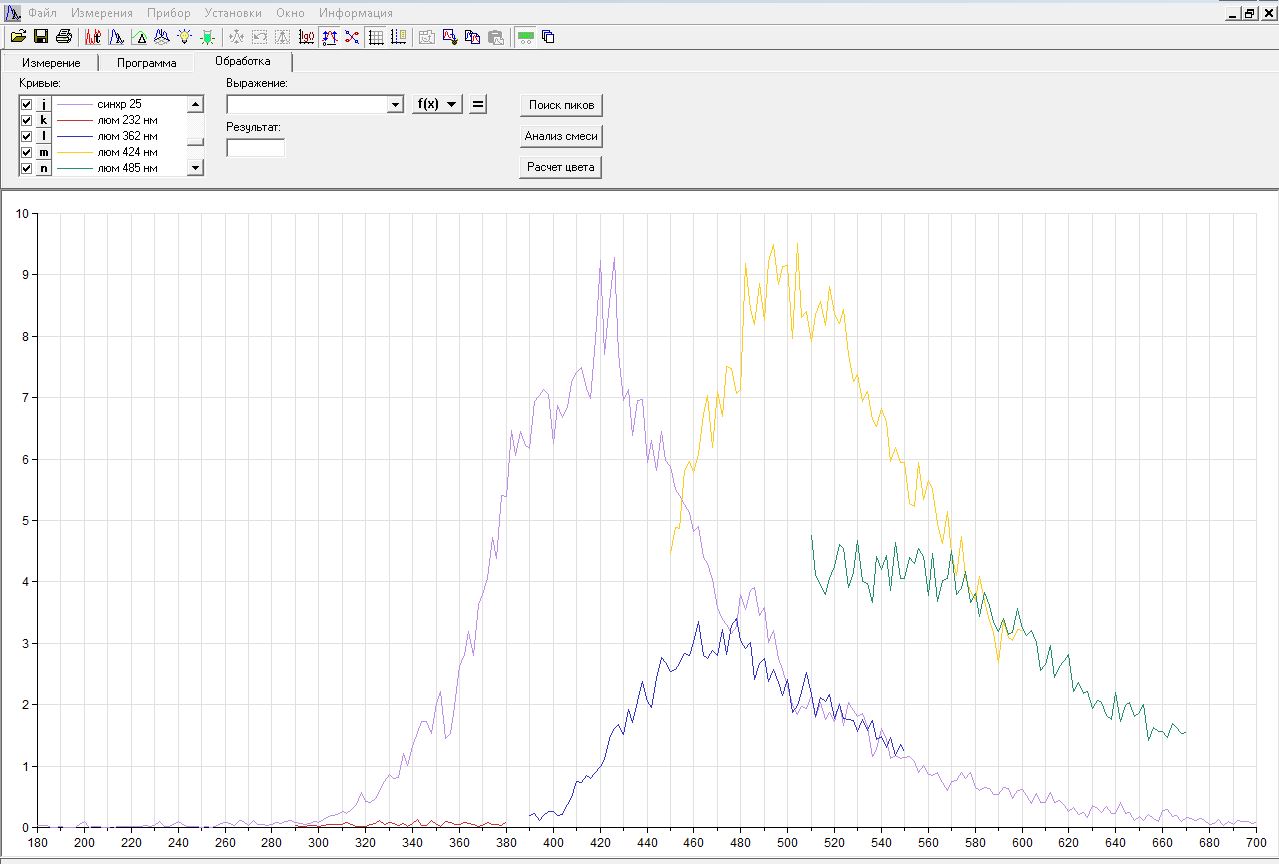

Supplement: S1 Data — (ZIP) [file pone.0267912.s001.zip › Primary Data/Oats/Infected/æ»Ñ¬Γαδ «óßá oáαáaÑ¡¡«ú« 25.JPG]

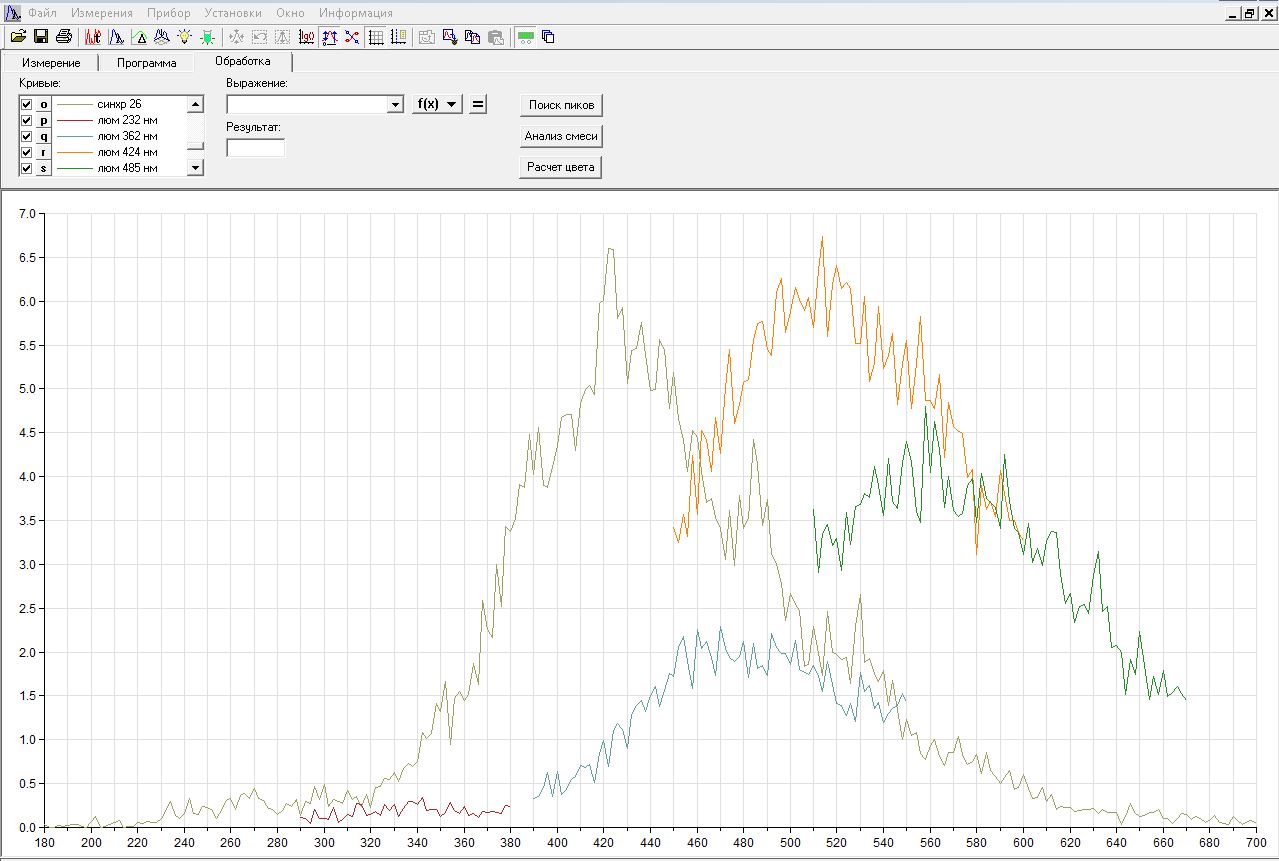

Supplement: S1 Data — (ZIP) [file pone.0267912.s001.zip › Primary Data/Oats/Infected/æ»Ñ¬Γαδ «óßá oáαáaÑ¡¡«ú« 26.JPG]

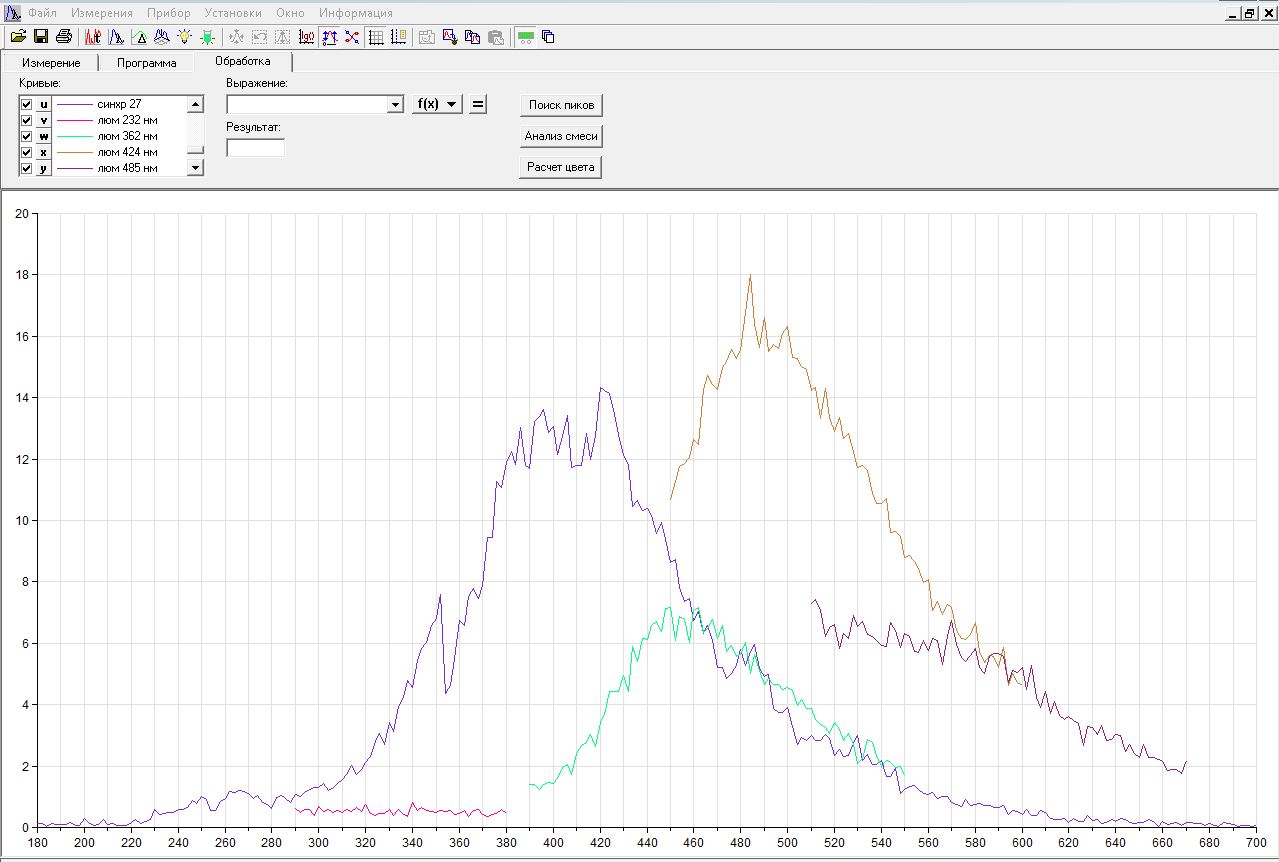

Supplement: S1 Data — (ZIP) [file pone.0267912.s001.zip › Primary Data/Oats/Infected/æ»Ñ¬Γαδ «óßá oáαáaÑ¡¡«ú« 27.JPG]

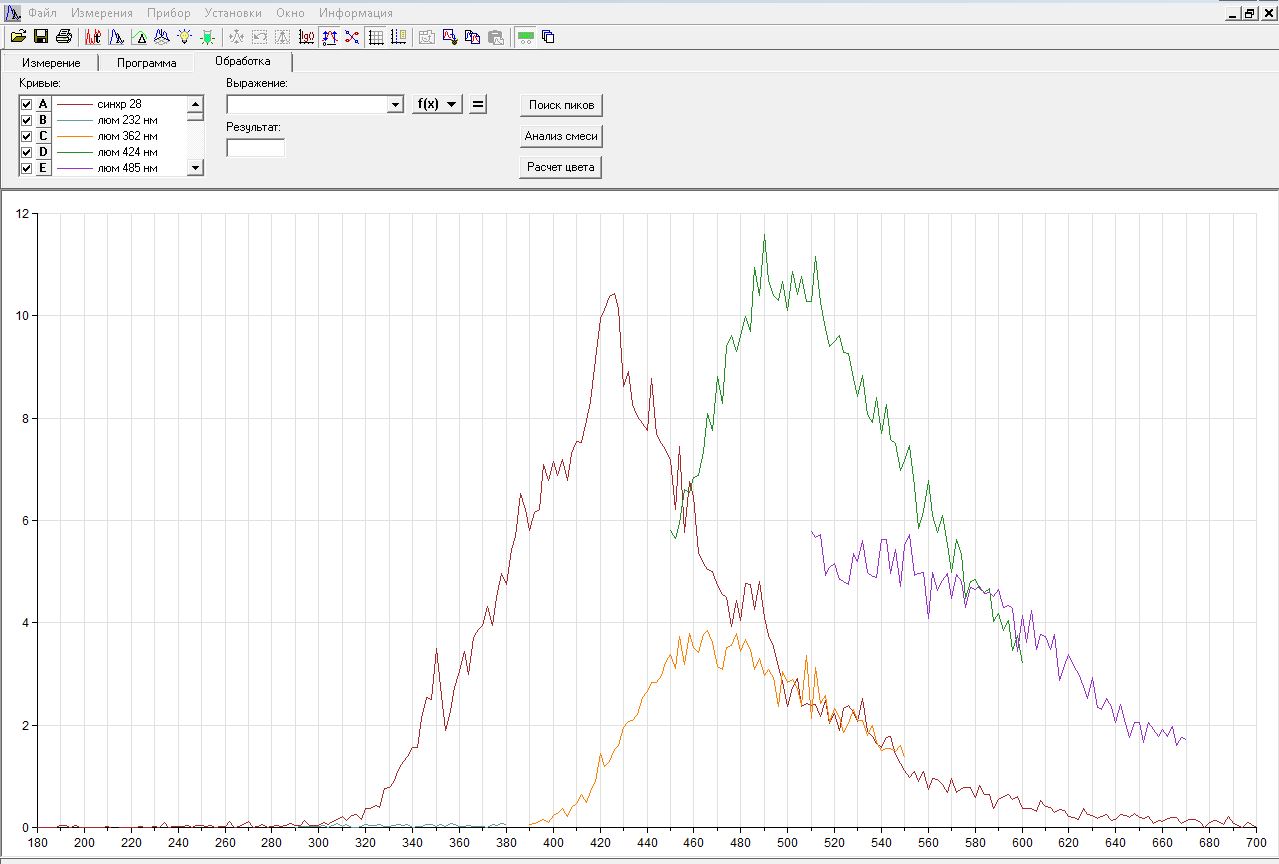

Supplement: S1 Data — (ZIP) [file pone.0267912.s001.zip › Primary Data/Oats/Infected/æ»Ñ¬Γαδ «óßá oáαáaÑ¡¡«ú« 28.JPG]

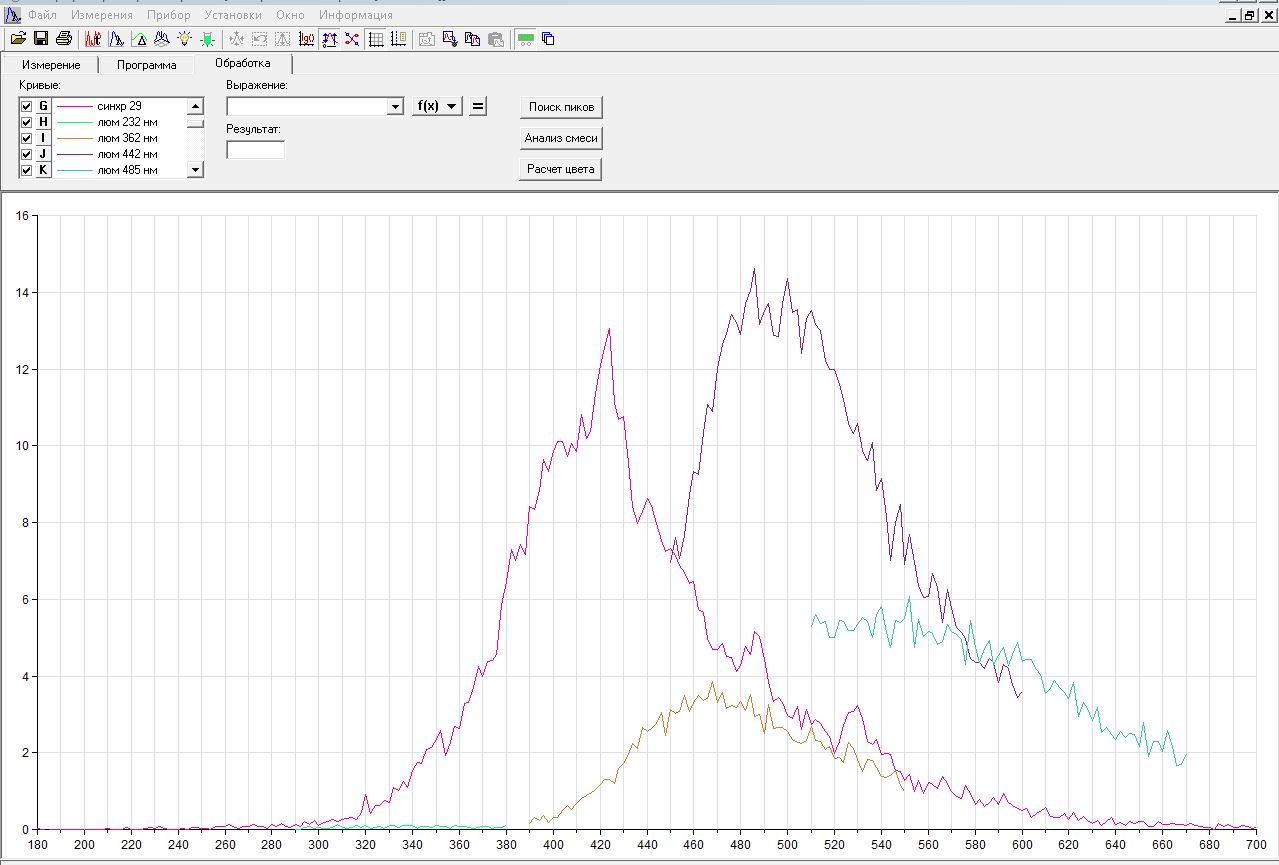

Supplement: S1 Data — (ZIP) [file pone.0267912.s001.zip › Primary Data/Oats/Infected/æ»Ñ¬Γαδ «óßá oáαáaÑ¡¡«ú« 29.JPG]

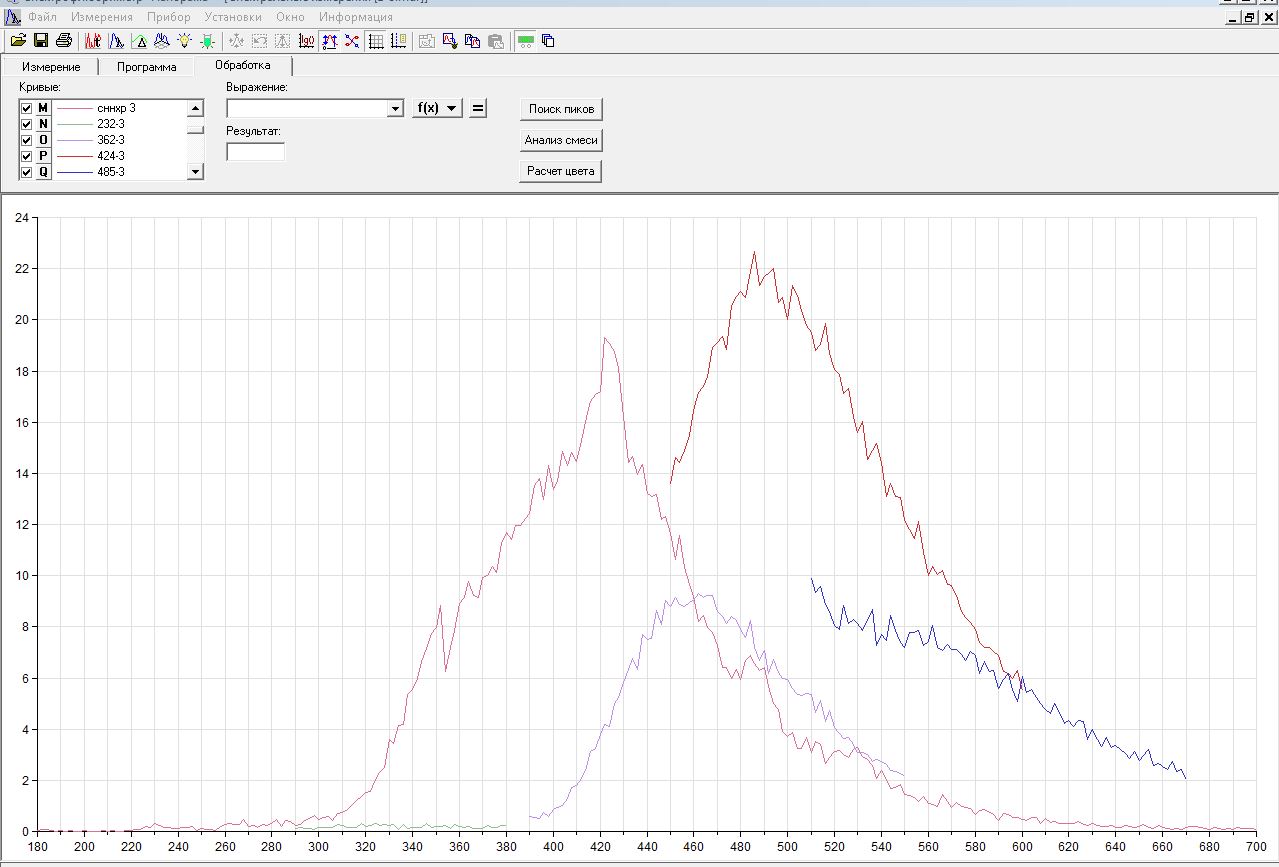

Supplement: S1 Data — (ZIP) [file pone.0267912.s001.zip › Primary Data/Oats/Infected/æ»Ñ¬Γαδ «óßá oáαáaÑ¡¡«ú« 3.JPG]

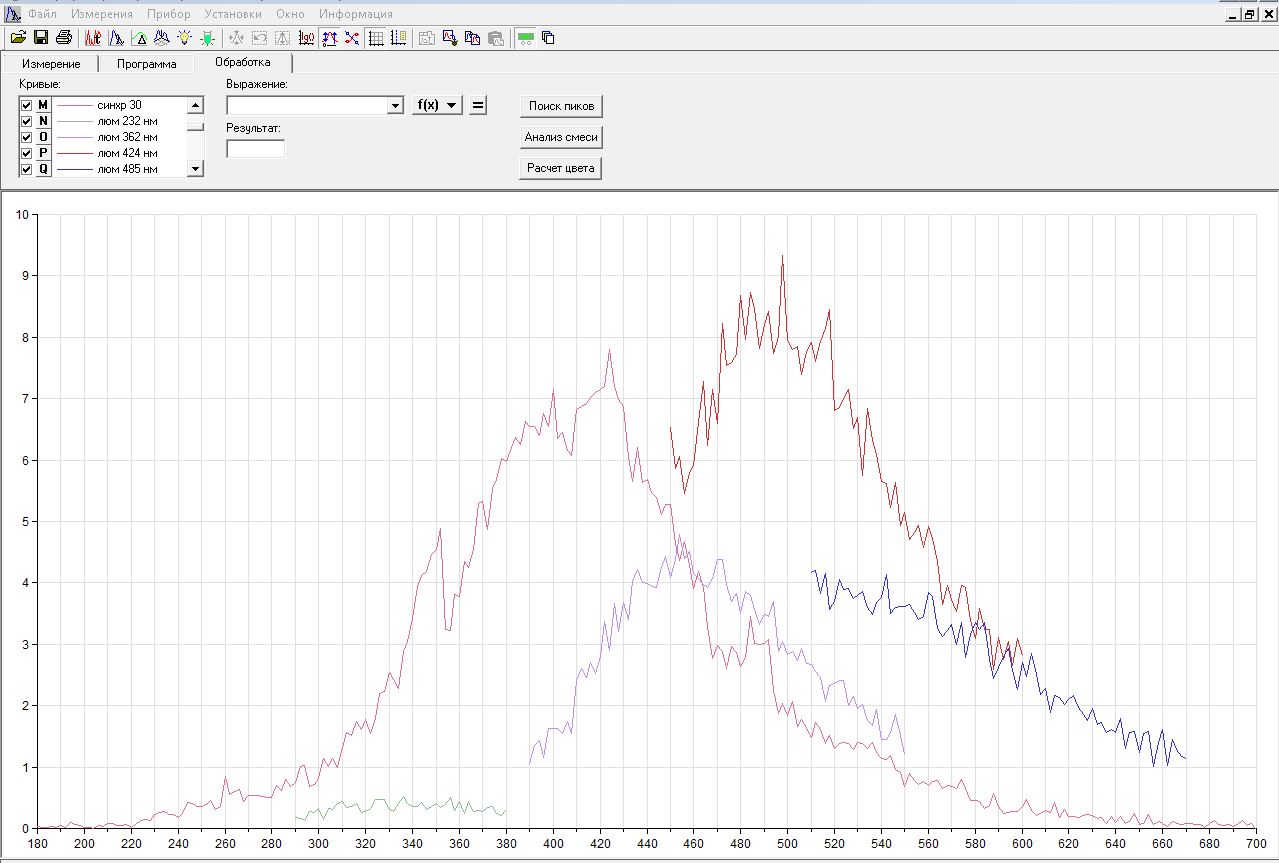

Supplement: S1 Data — (ZIP) [file pone.0267912.s001.zip › Primary Data/Oats/Infected/æ»Ñ¬Γαδ «óßá oáαáaÑ¡¡«ú« 30.JPG]

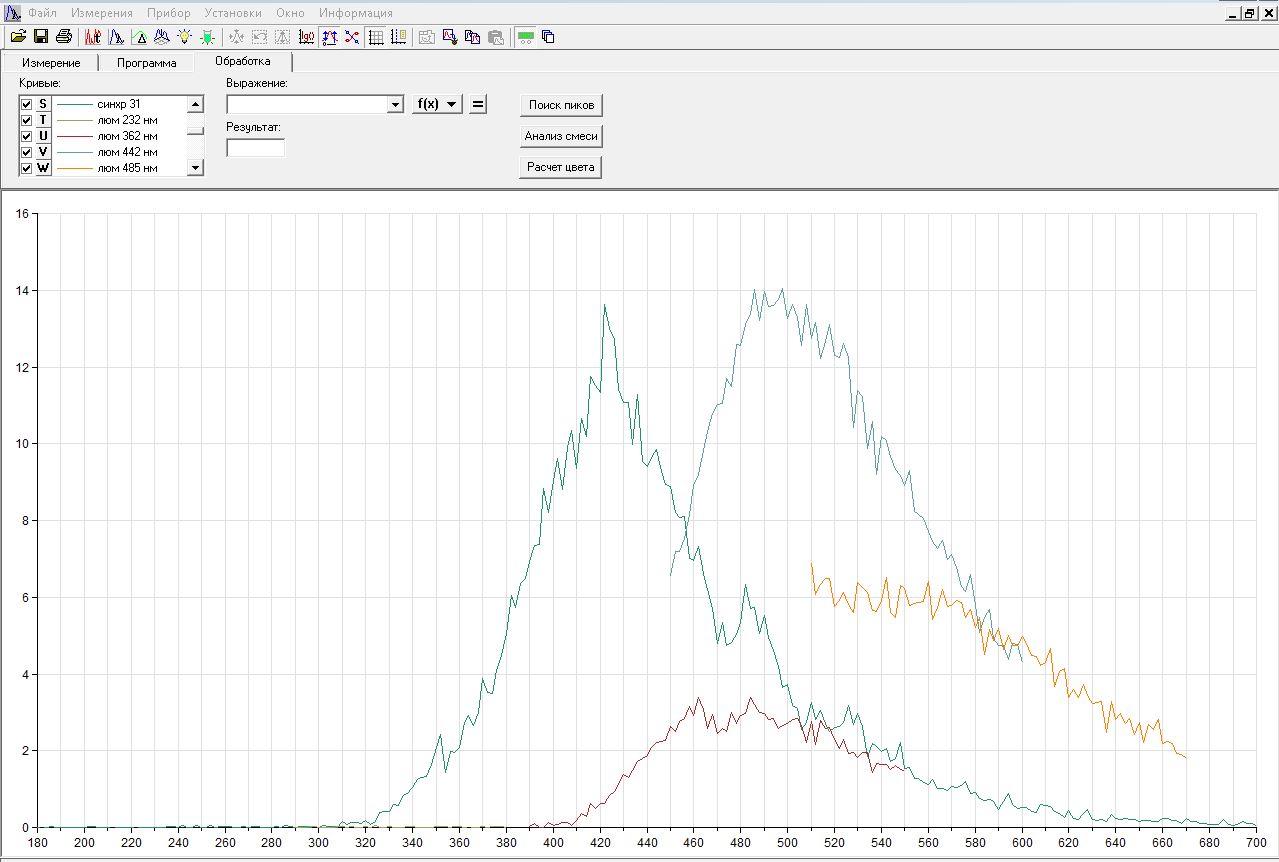

Supplement: S1 Data — (ZIP) [file pone.0267912.s001.zip › Primary Data/Oats/Infected/æ»Ñ¬Γαδ «óßá oáαáaÑ¡¡«ú« 31.JPG]

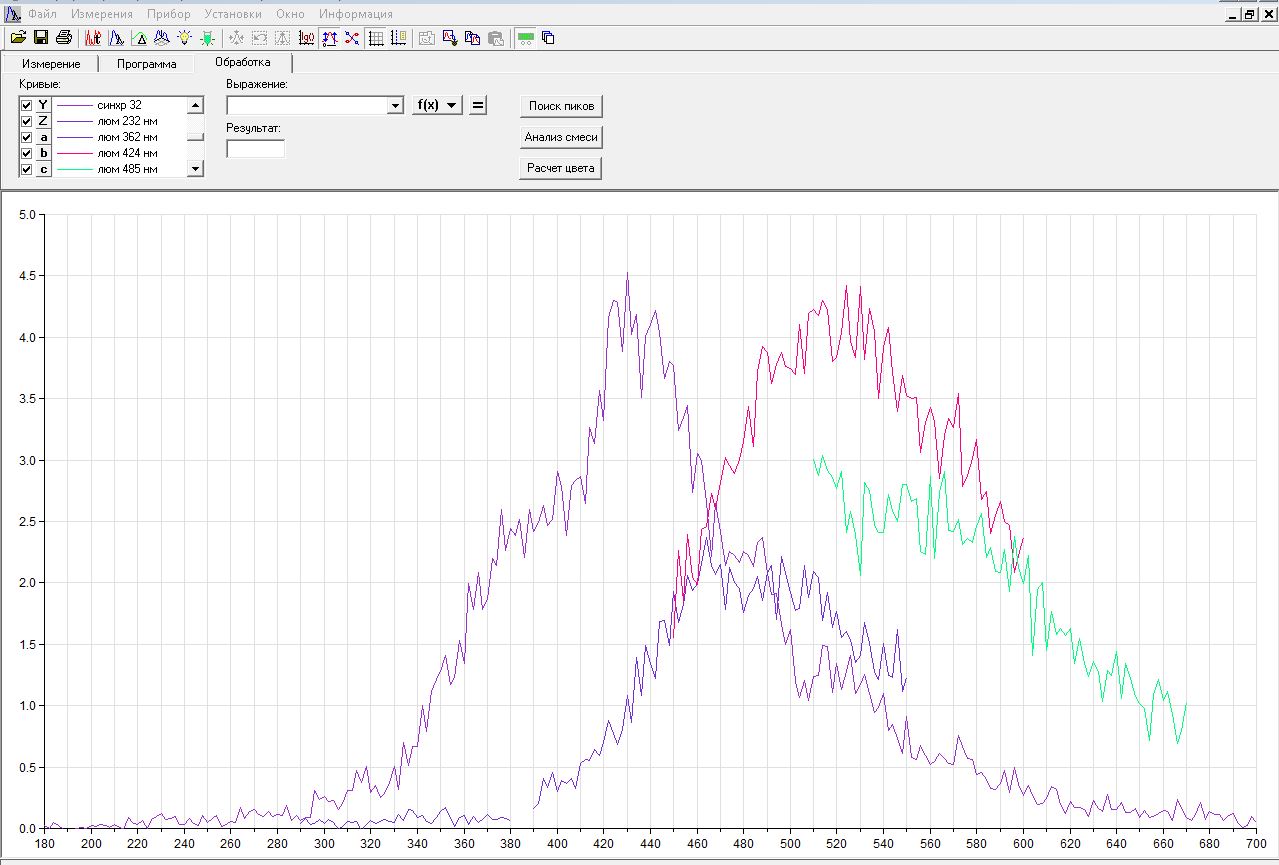

Supplement: S1 Data — (ZIP) [file pone.0267912.s001.zip › Primary Data/Oats/Infected/æ»Ñ¬Γαδ «óßá oáαáaÑ¡¡«ú« 32.JPG]

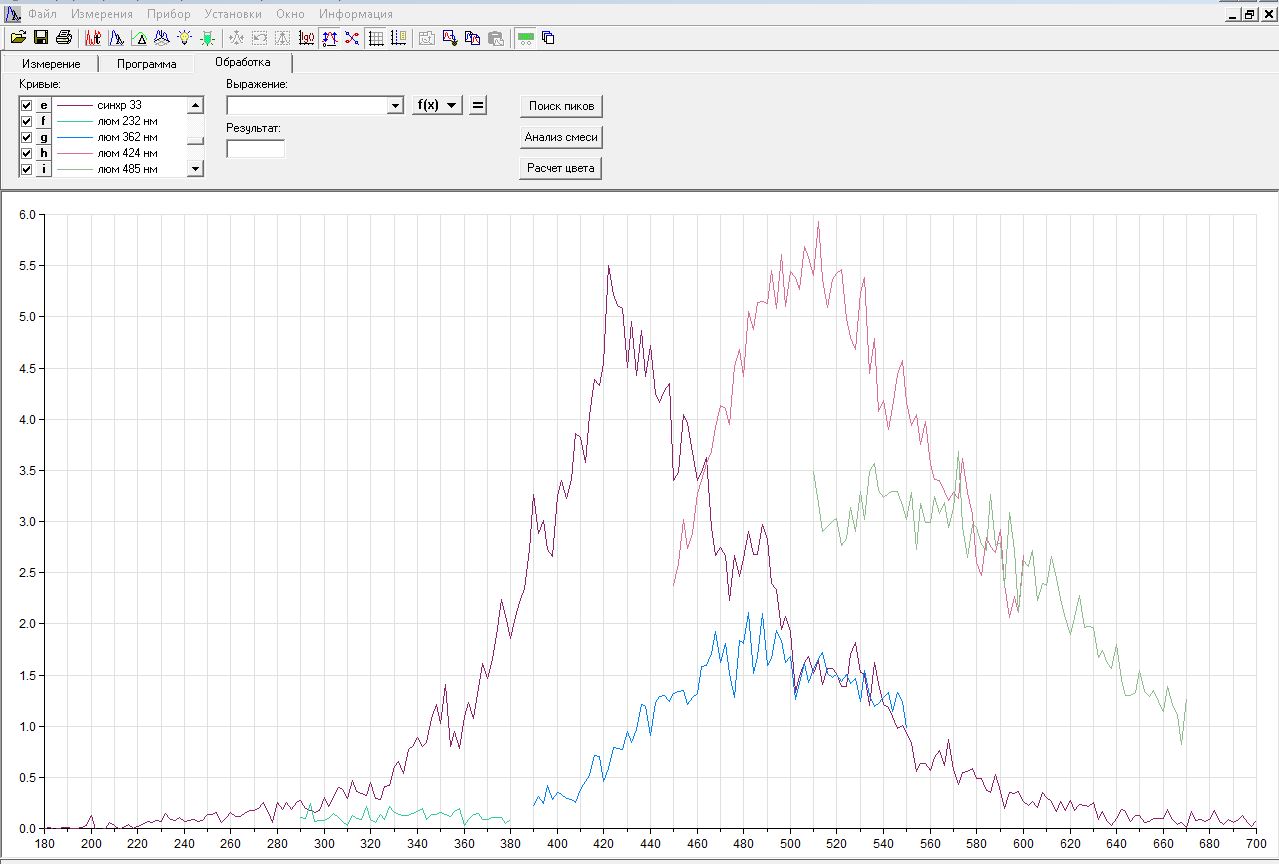

Supplement: S1 Data — (ZIP) [file pone.0267912.s001.zip › Primary Data/Oats/Infected/æ»Ñ¬Γαδ «óßá oáαáaÑ¡¡«ú« 33.JPG]

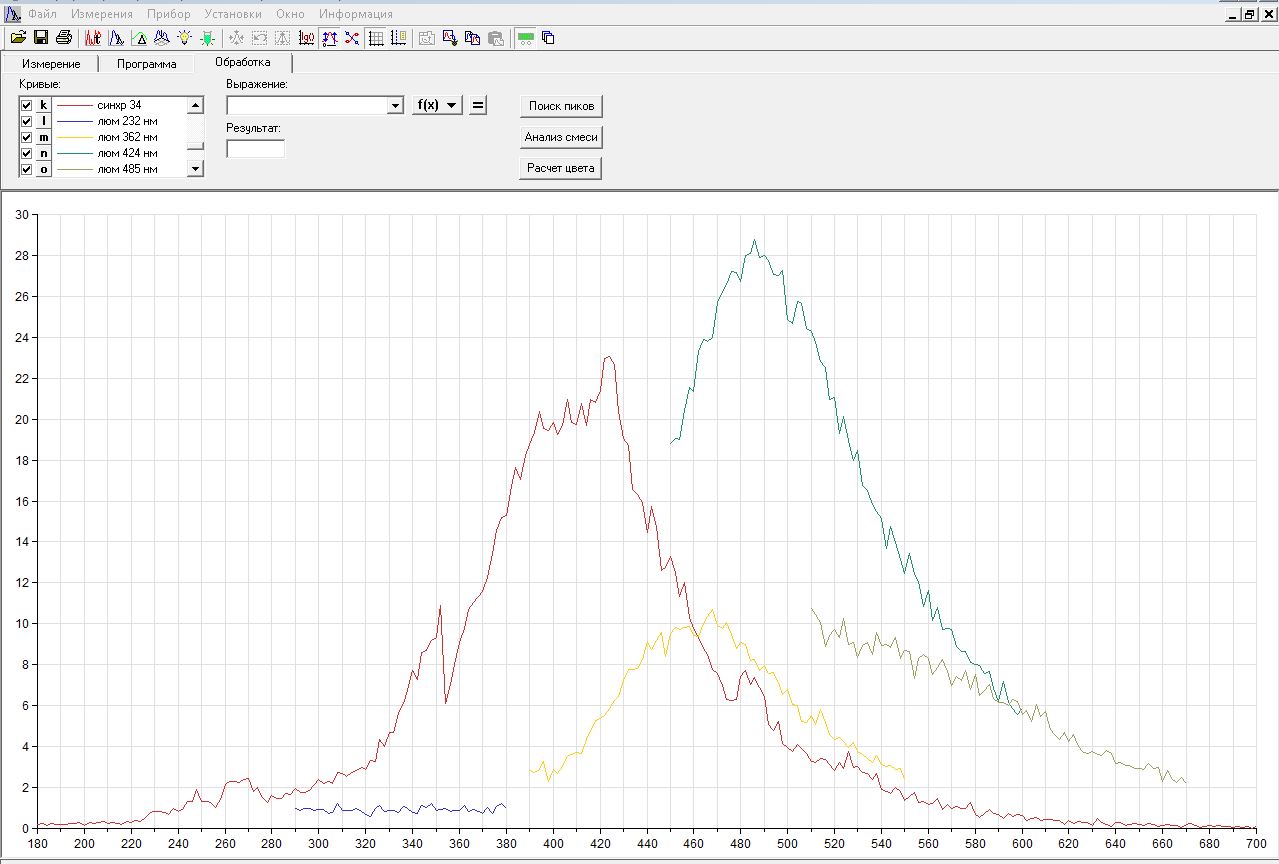

Supplement: S1 Data — (ZIP) [file pone.0267912.s001.zip › Primary Data/Oats/Infected/æ»Ñ¬Γαδ «óßá oáαáaÑ¡¡«ú« 34.JPG]

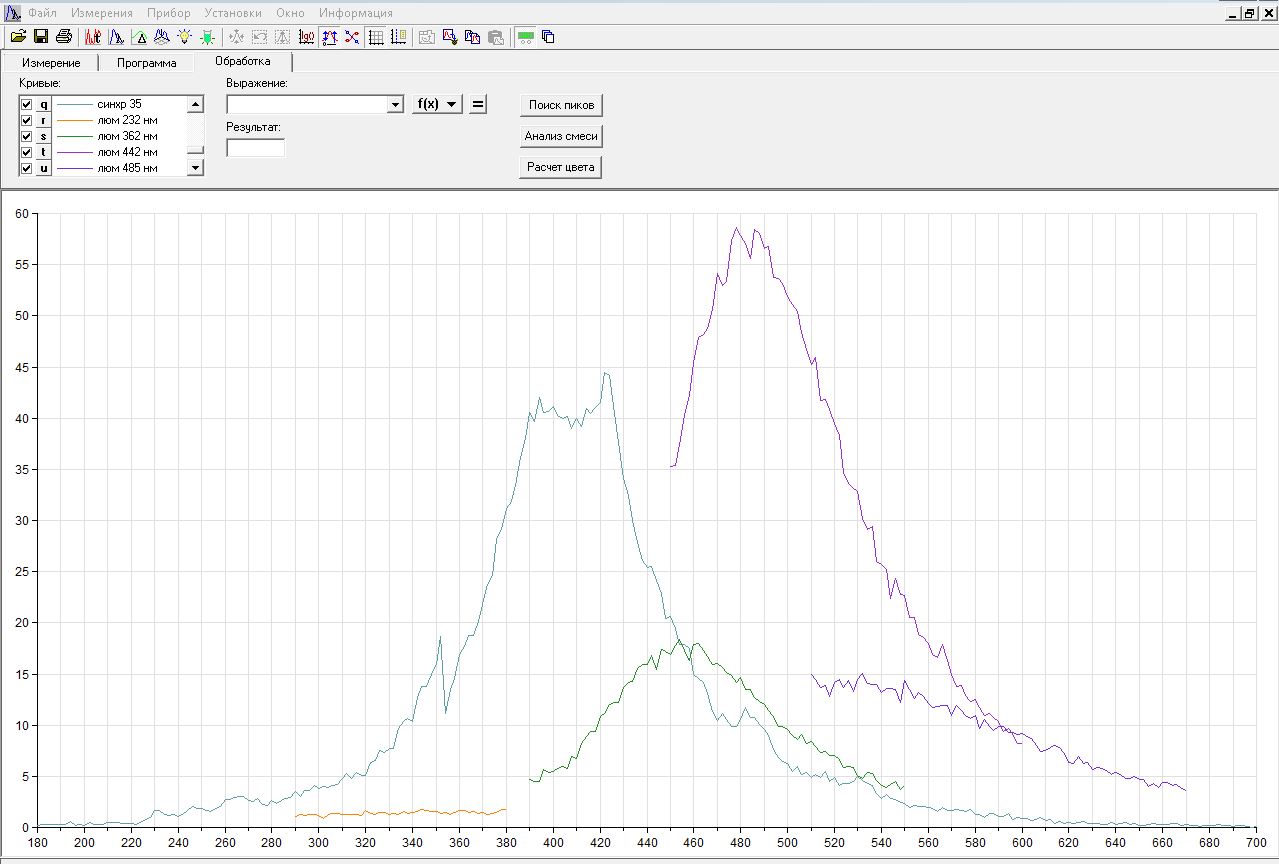

Supplement: S1 Data — (ZIP) [file pone.0267912.s001.zip › Primary Data/Oats/Infected/æ»Ñ¬Γαδ «óßá oáαáaÑ¡¡«ú« 35.JPG]

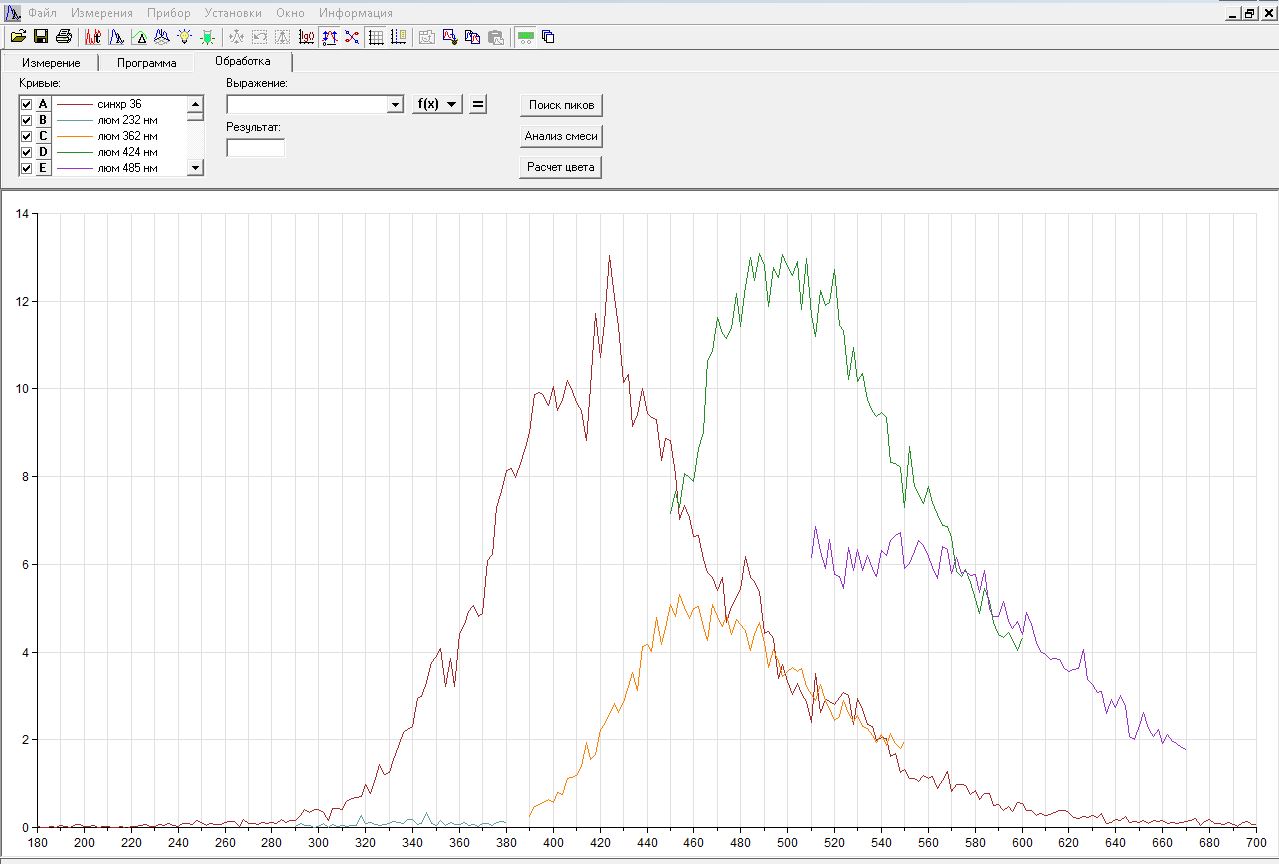

Supplement: S1 Data — (ZIP) [file pone.0267912.s001.zip › Primary Data/Oats/Infected/æ»Ñ¬Γαδ «óßá oáαáaÑ¡¡«ú« 36.JPG]

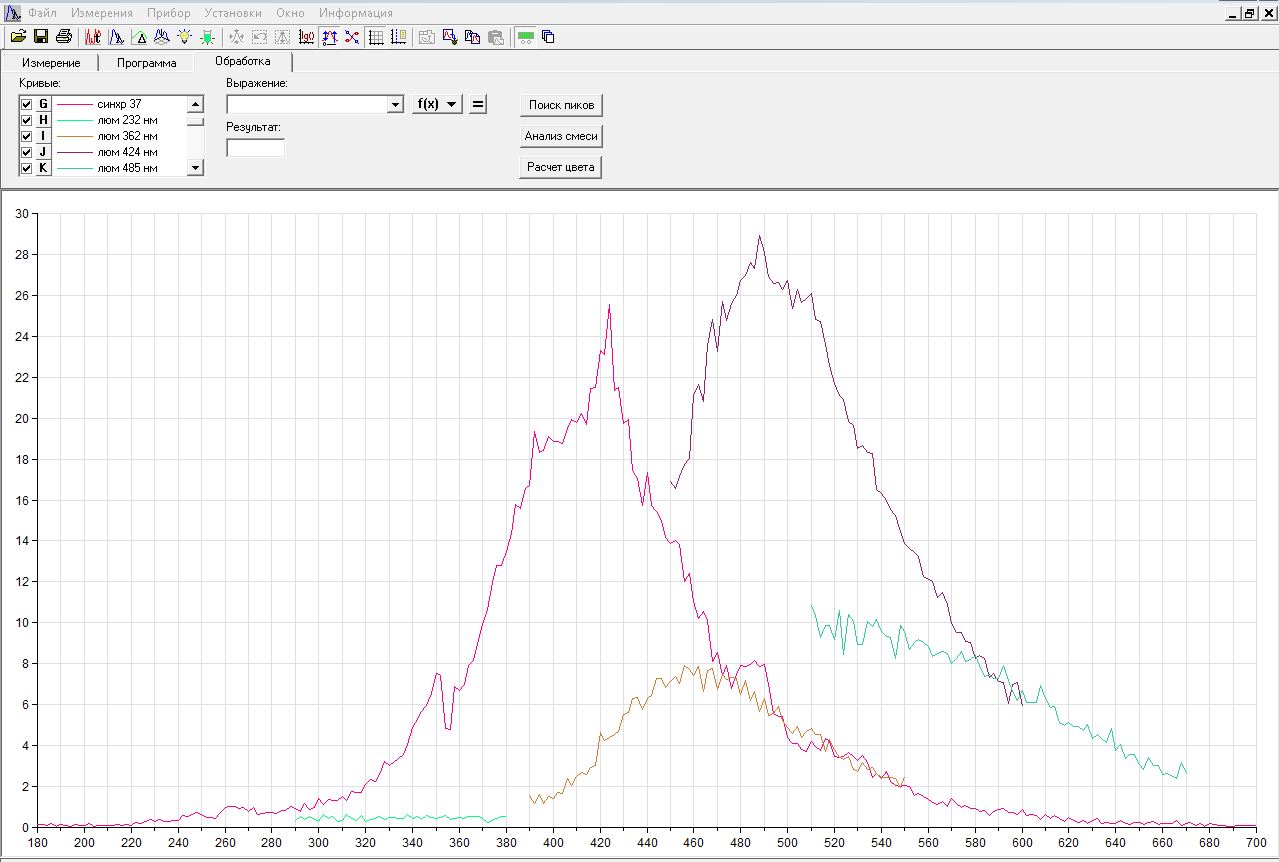

Supplement: S1 Data — (ZIP) [file pone.0267912.s001.zip › Primary Data/Oats/Infected/æ»Ñ¬Γαδ «óßá oáαáaÑ¡¡«ú« 37.JPG]

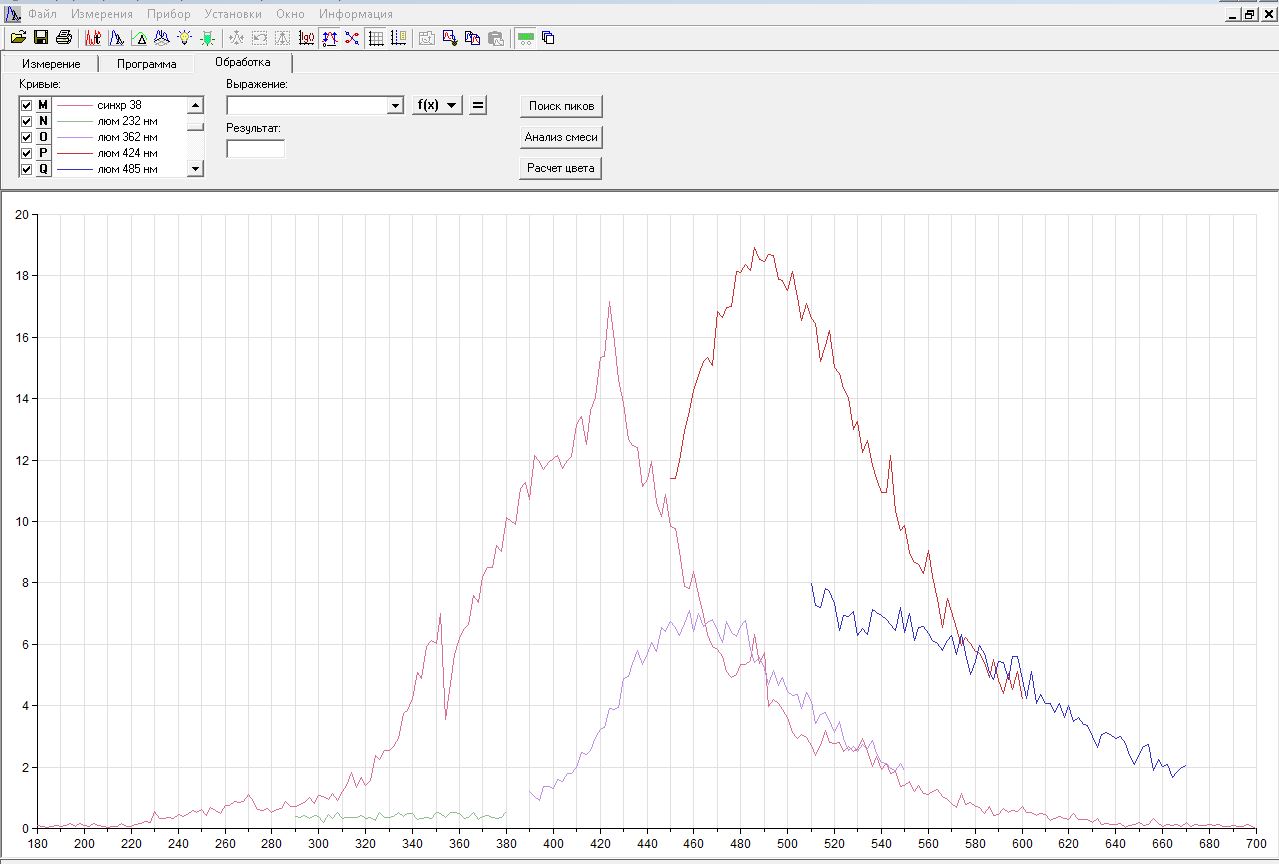

Supplement: S1 Data — (ZIP) [file pone.0267912.s001.zip › Primary Data/Oats/Infected/æ»Ñ¬Γαδ «óßá oáαáaÑ¡¡«ú« 38.JPG]

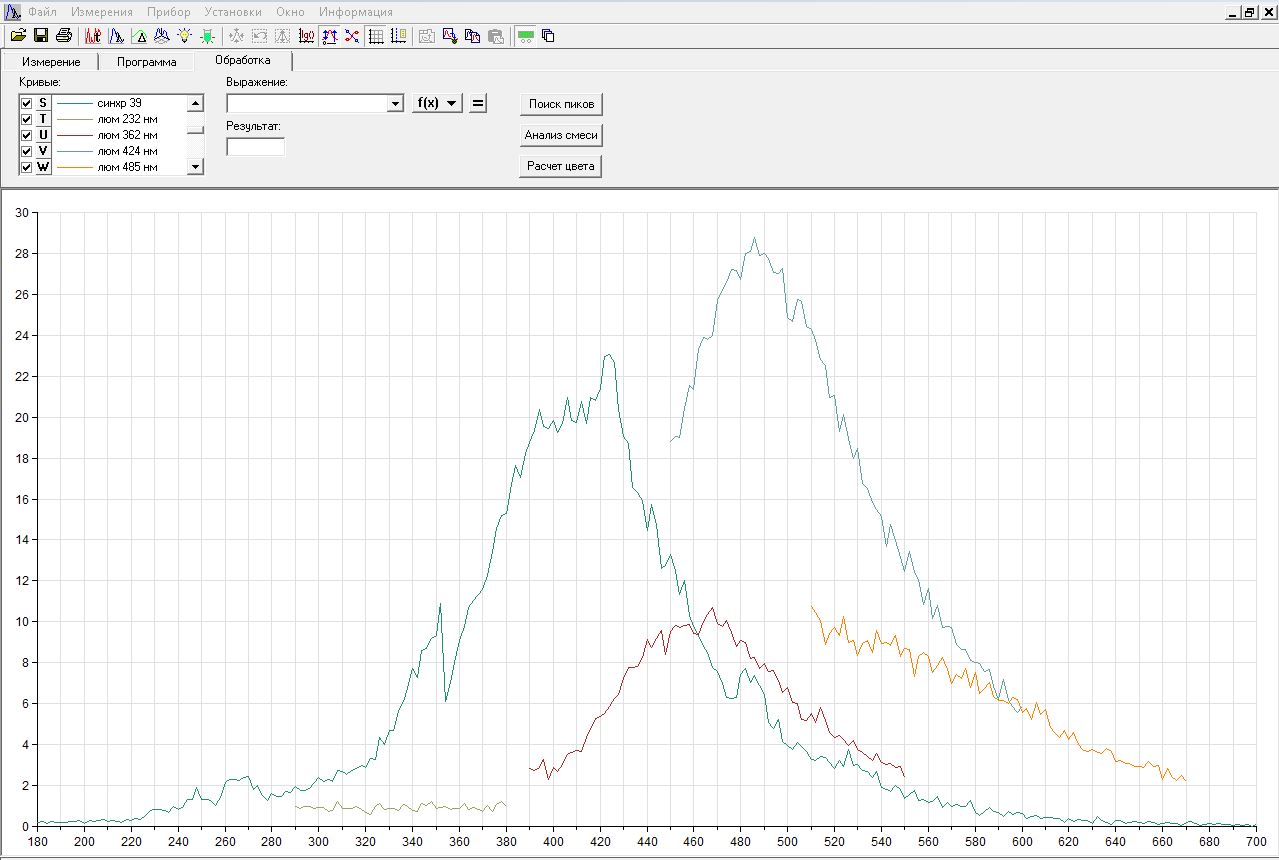

Supplement: S1 Data — (ZIP) [file pone.0267912.s001.zip › Primary Data/Oats/Infected/æ»Ñ¬Γαδ «óßá oáαáaÑ¡¡«ú« 39.JPG]

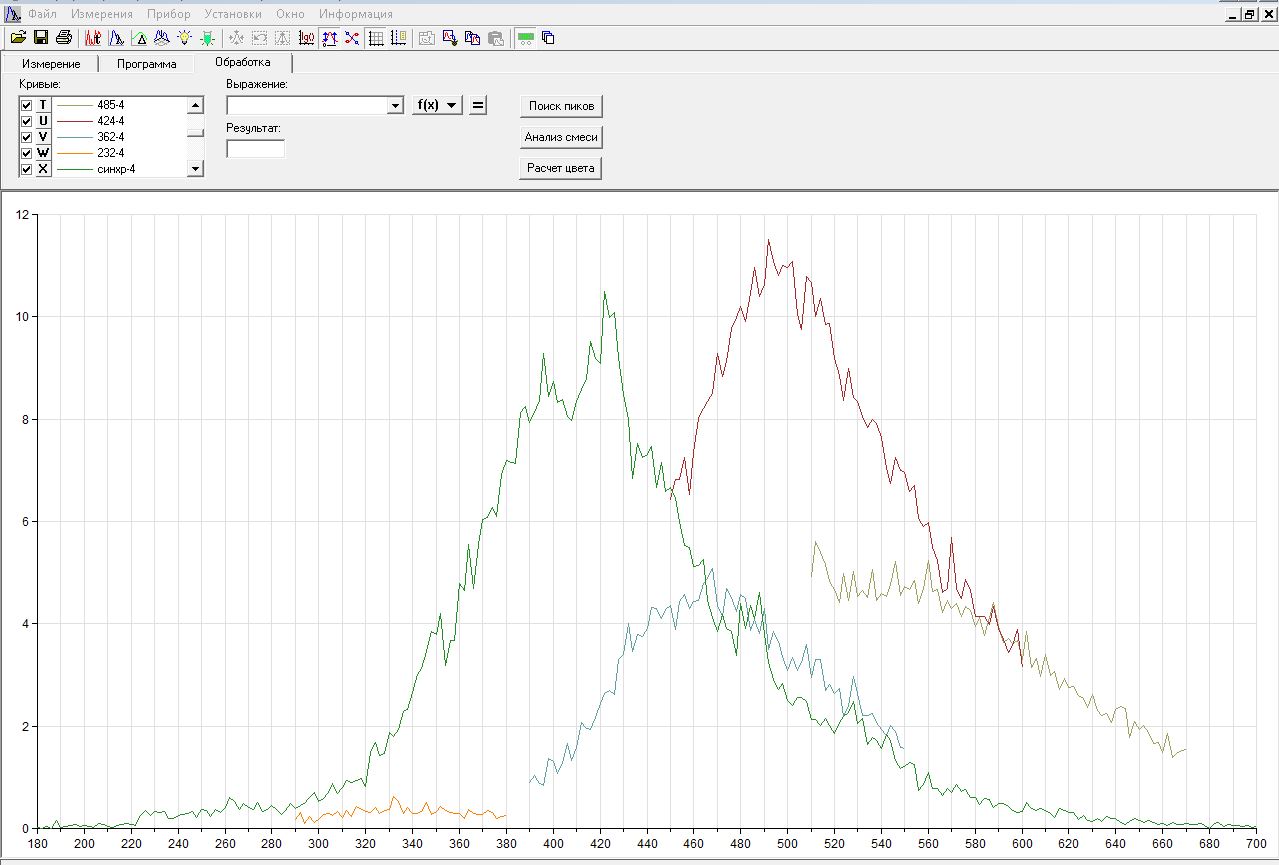

Supplement: S1 Data — (ZIP) [file pone.0267912.s001.zip › Primary Data/Oats/Infected/æ»Ñ¬Γαδ «óßá oáαáaÑ¡¡«ú« 4.JPG]

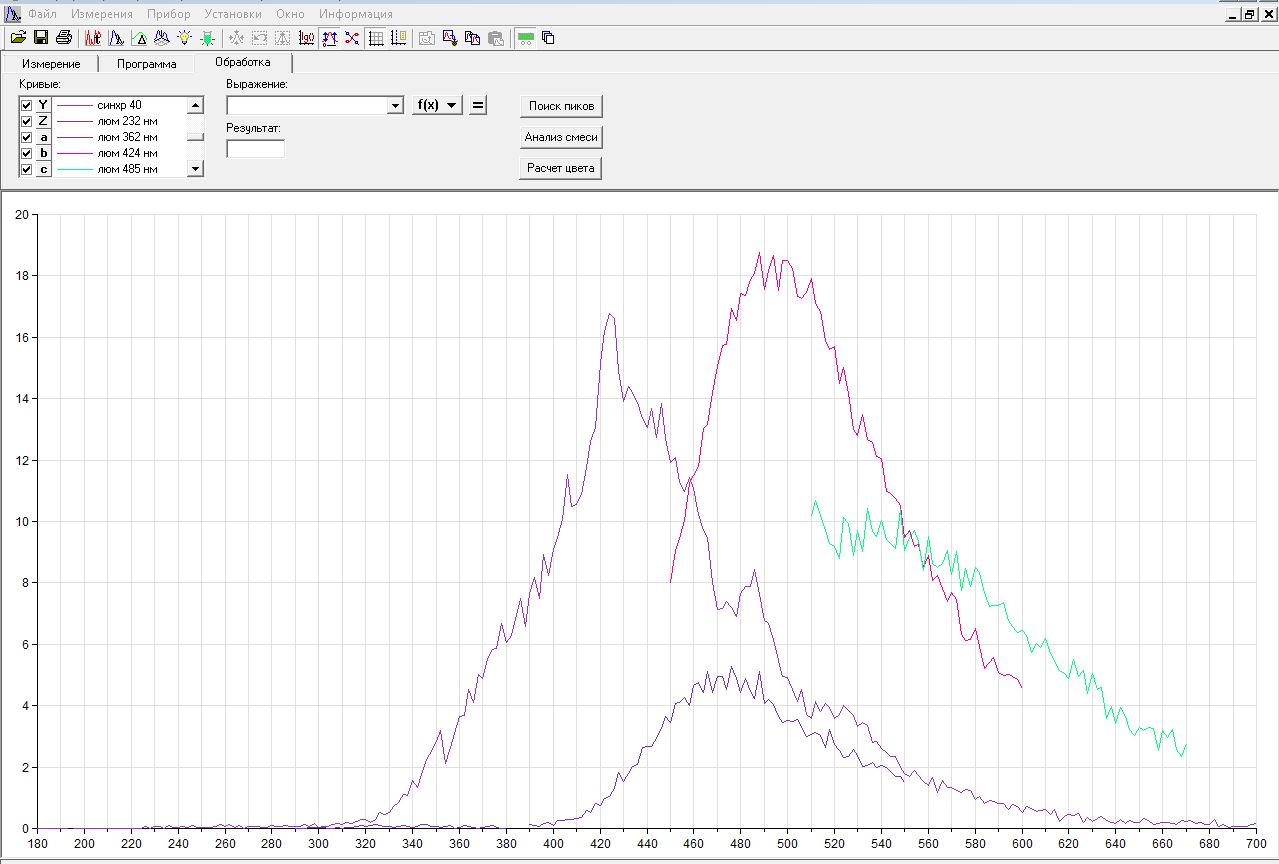

Supplement: S1 Data — (ZIP) [file pone.0267912.s001.zip › Primary Data/Oats/Infected/æ»Ñ¬Γαδ «óßá oáαáaÑ¡¡«ú« 40.JPG]

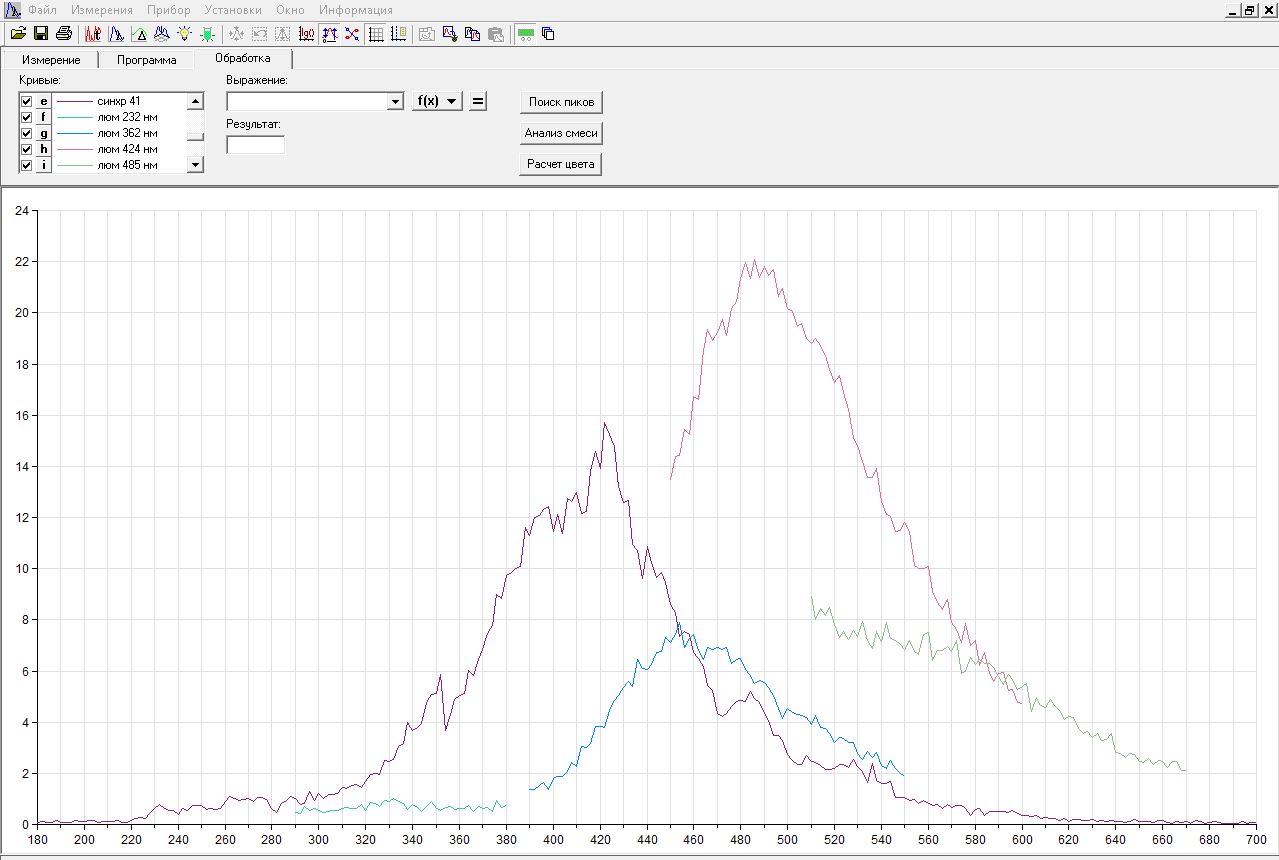

Supplement: S1 Data — (ZIP) [file pone.0267912.s001.zip › Primary Data/Oats/Infected/æ»Ñ¬Γαδ «óßá oáαáaÑ¡¡«ú« 41.JPG]

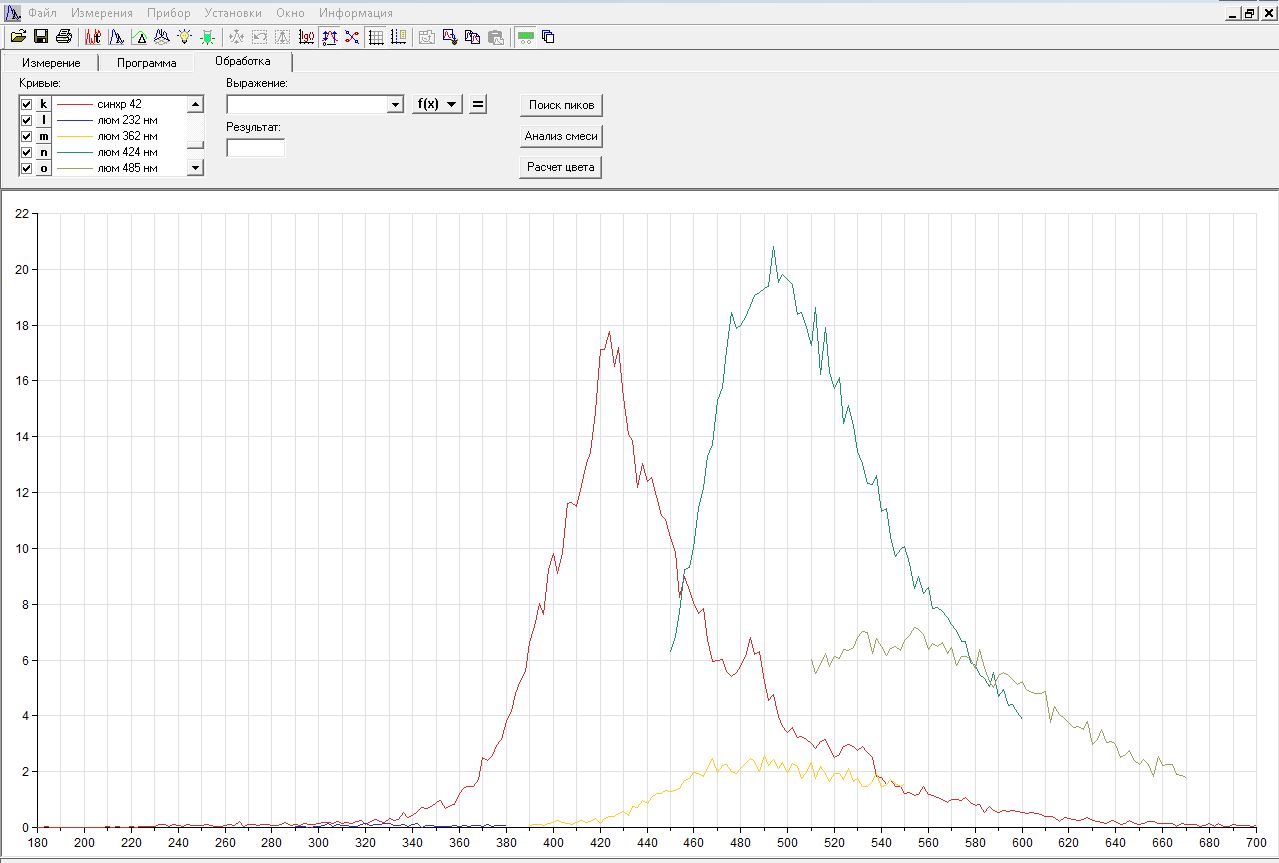

Supplement: S1 Data — (ZIP) [file pone.0267912.s001.zip › Primary Data/Oats/Infected/æ»Ñ¬Γαδ «óßá oáαáaÑ¡¡«ú« 42.JPG]

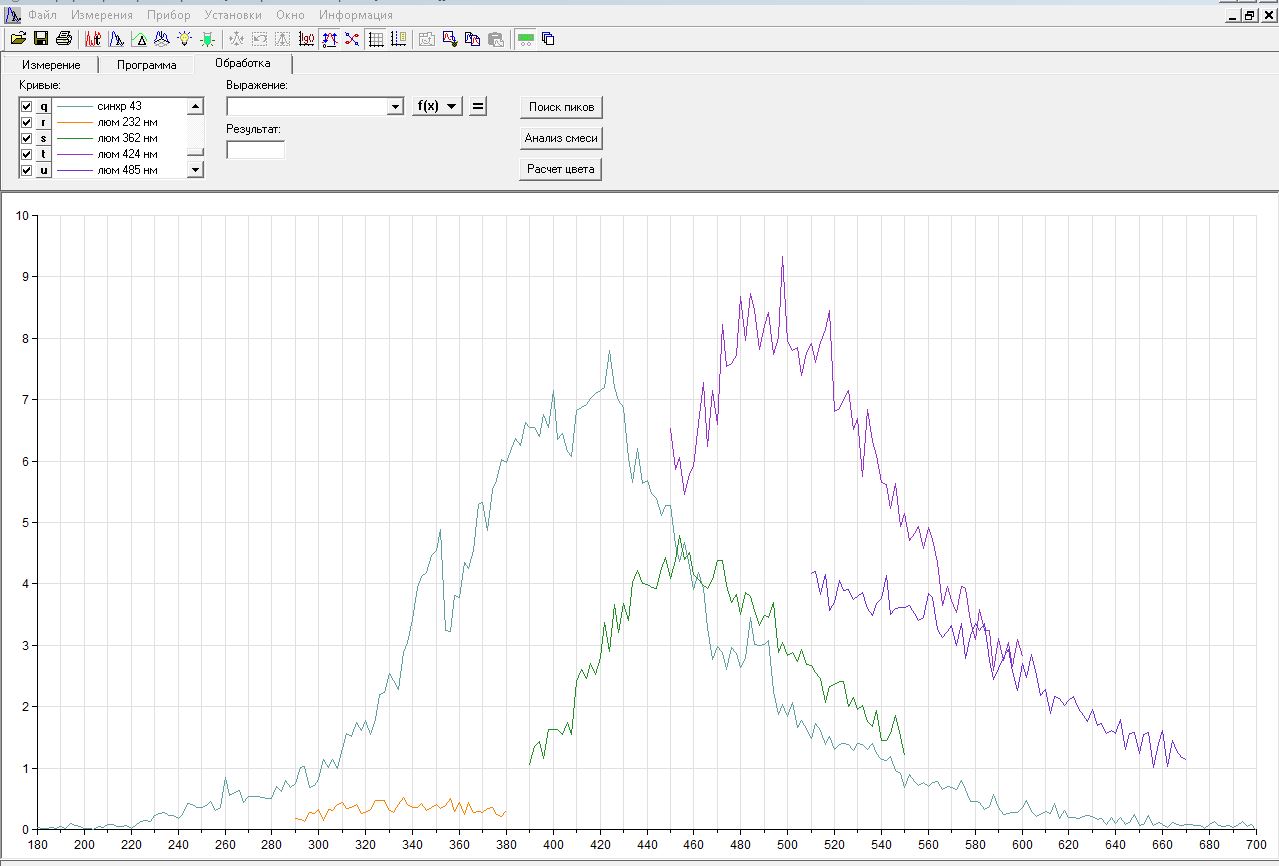

Supplement: S1 Data — (ZIP) [file pone.0267912.s001.zip › Primary Data/Oats/Infected/æ»Ñ¬Γαδ «óßá oáαáaÑ¡¡«ú« 43.JPG]

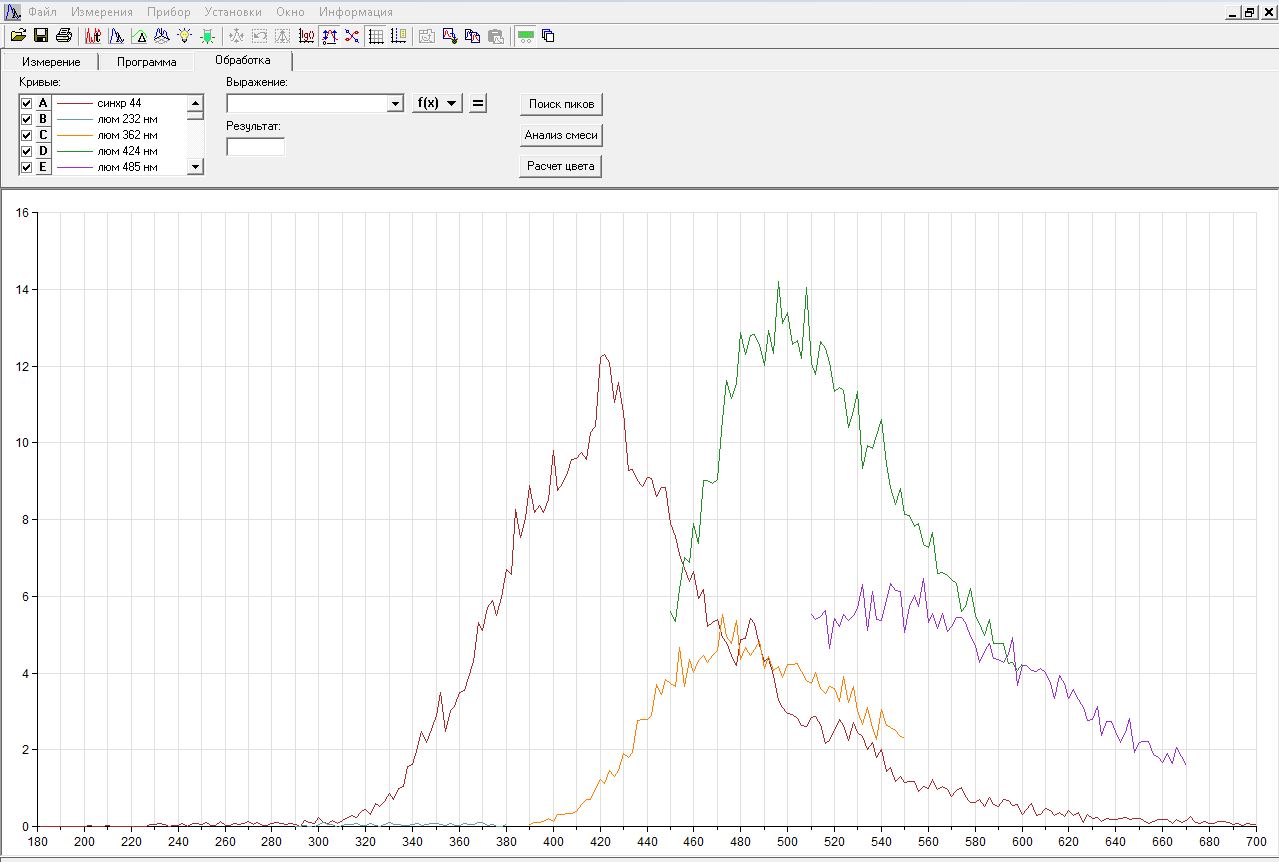

Supplement: S1 Data — (ZIP) [file pone.0267912.s001.zip › Primary Data/Oats/Infected/æ»Ñ¬Γαδ «óßá oáαáaÑ¡¡«ú« 44.JPG]

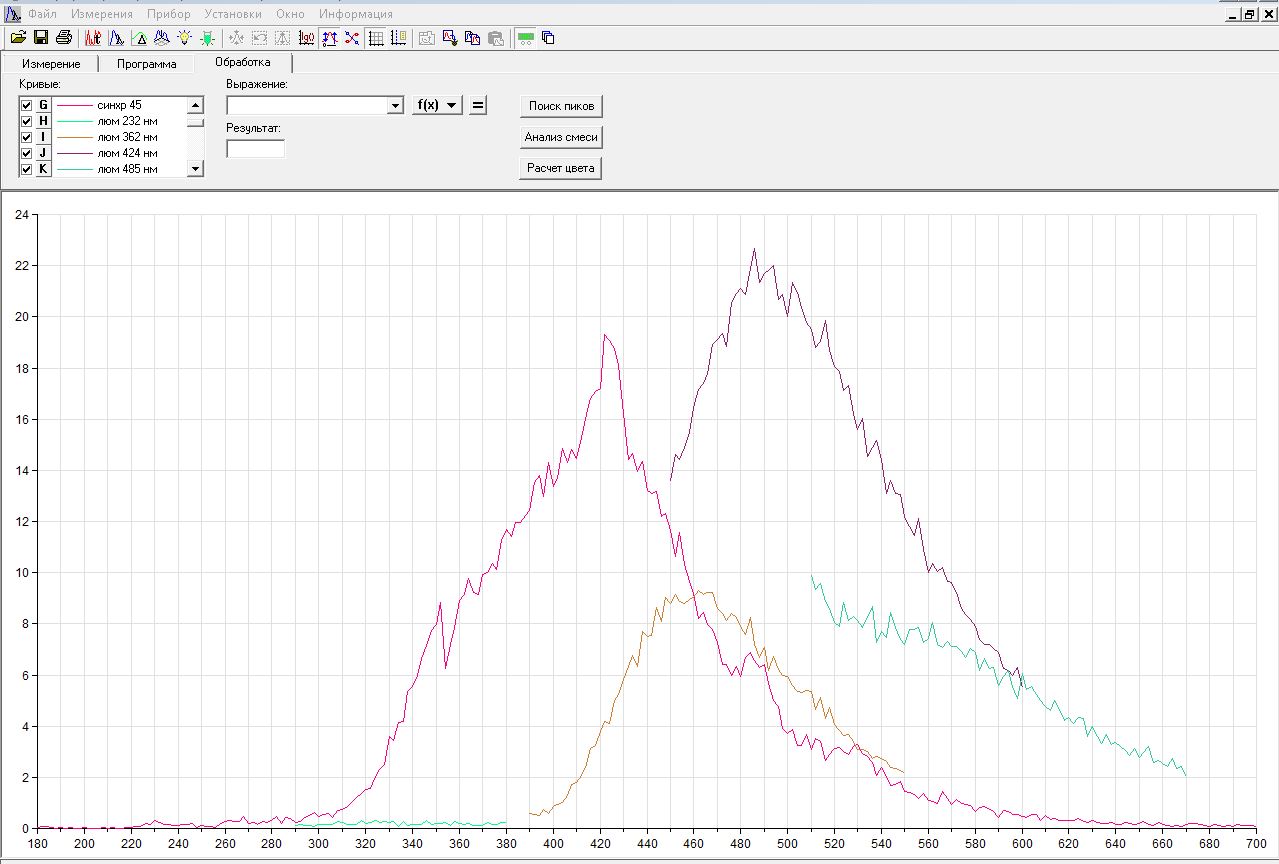

Supplement: S1 Data — (ZIP) [file pone.0267912.s001.zip › Primary Data/Oats/Infected/æ»Ñ¬Γαδ «óßá oáαáaÑ¡¡«ú« 45.JPG]

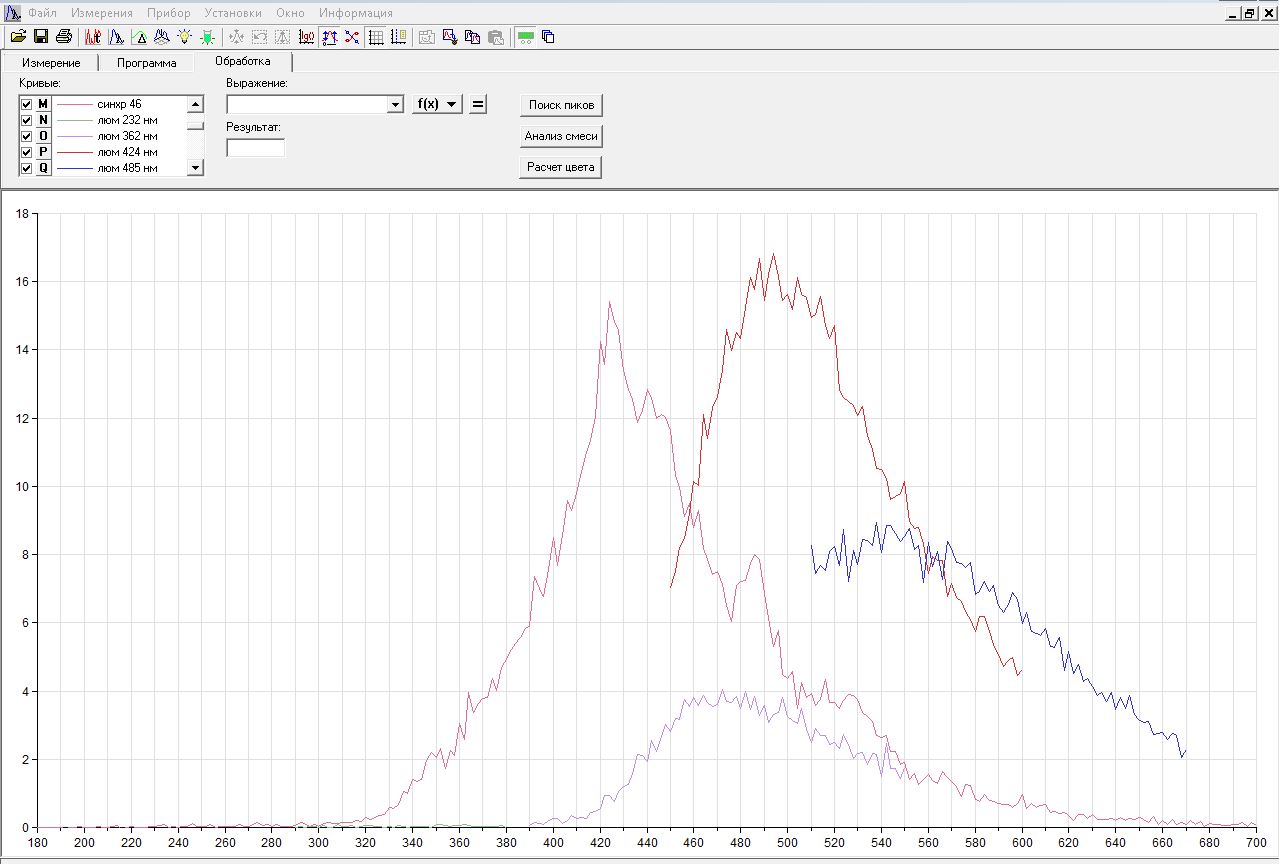

Supplement: S1 Data — (ZIP) [file pone.0267912.s001.zip › Primary Data/Oats/Infected/æ»Ñ¬Γαδ «óßá oáαáaÑ¡¡«ú« 46.JPG]

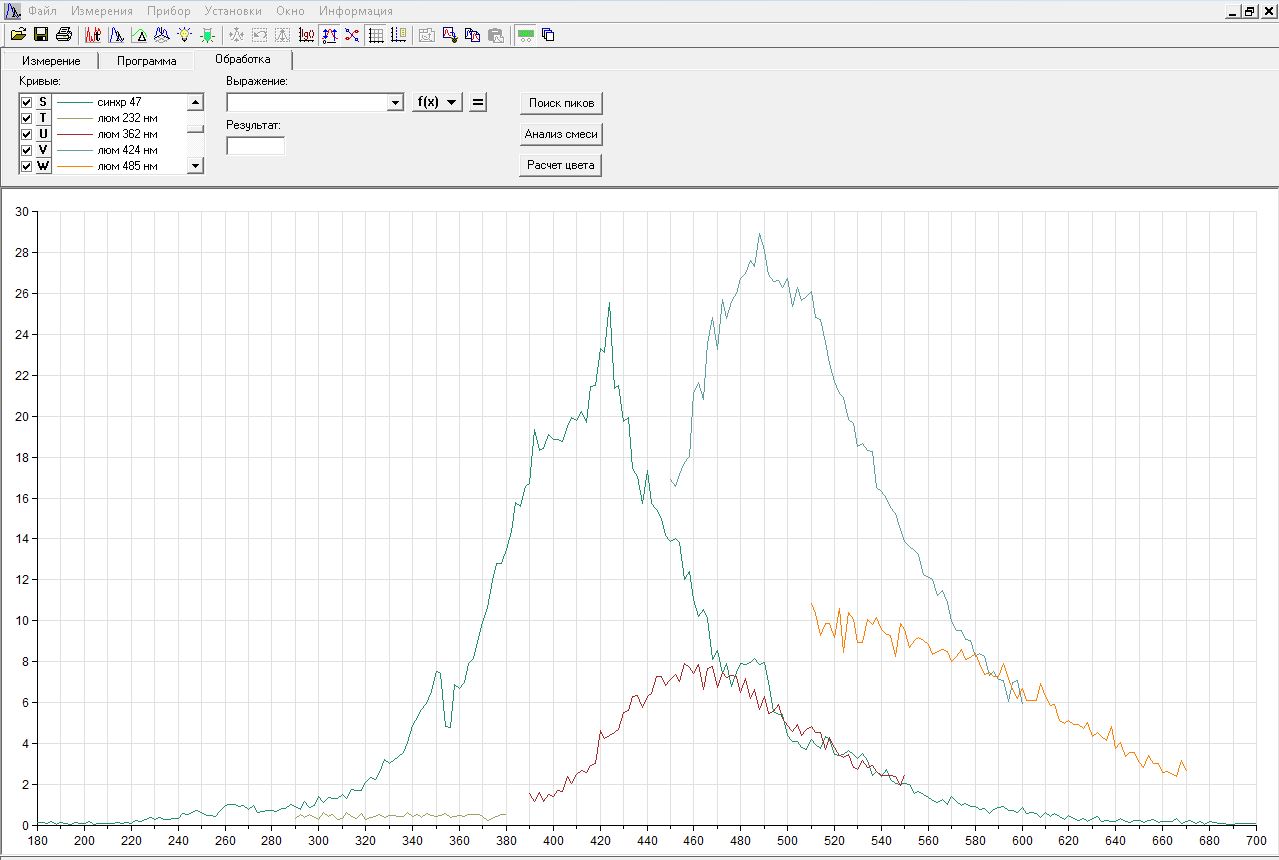

Supplement: S1 Data — (ZIP) [file pone.0267912.s001.zip › Primary Data/Oats/Infected/æ»Ñ¬Γαδ «óßá oáαáaÑ¡¡«ú« 47.JPG]

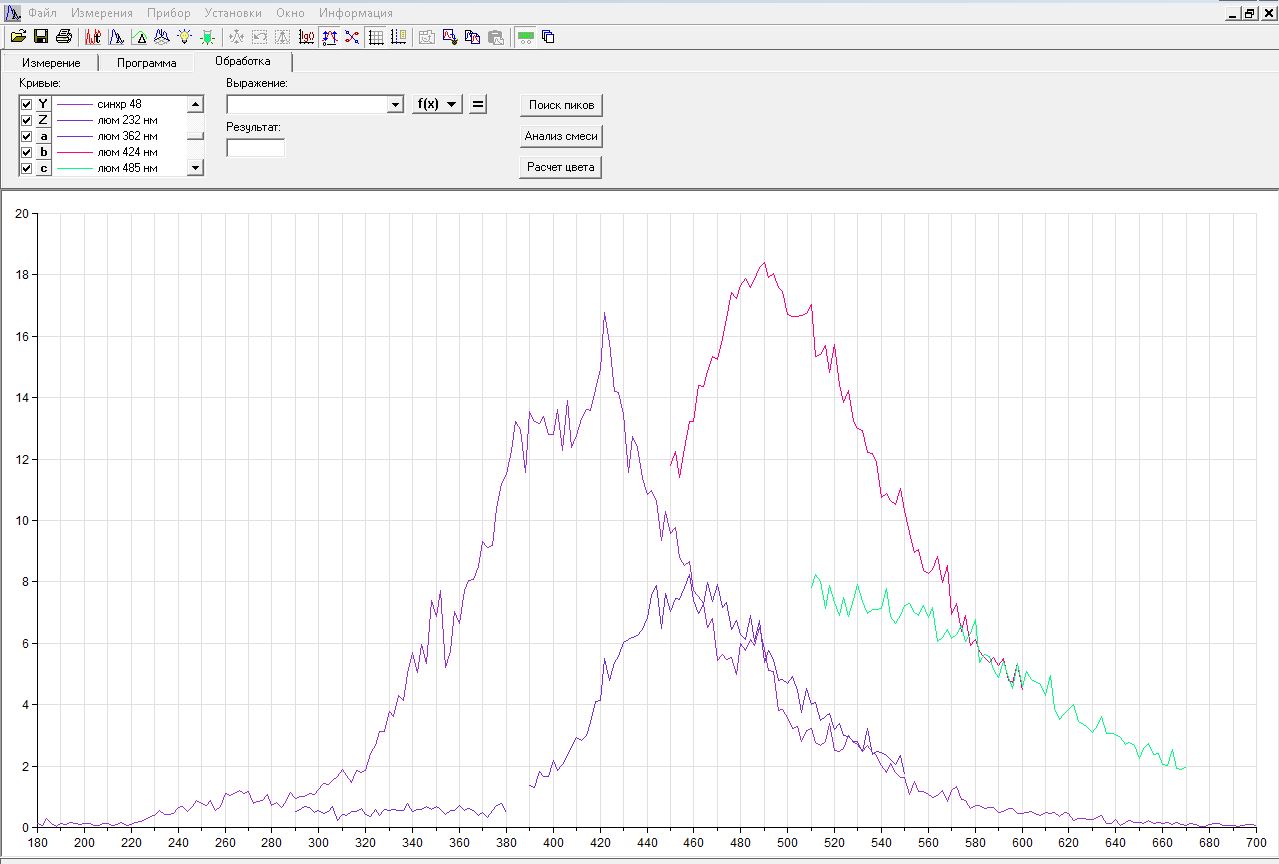

Supplement: S1 Data — (ZIP) [file pone.0267912.s001.zip › Primary Data/Oats/Infected/æ»Ñ¬Γαδ «óßá oáαáaÑ¡¡«ú« 48.JPG]

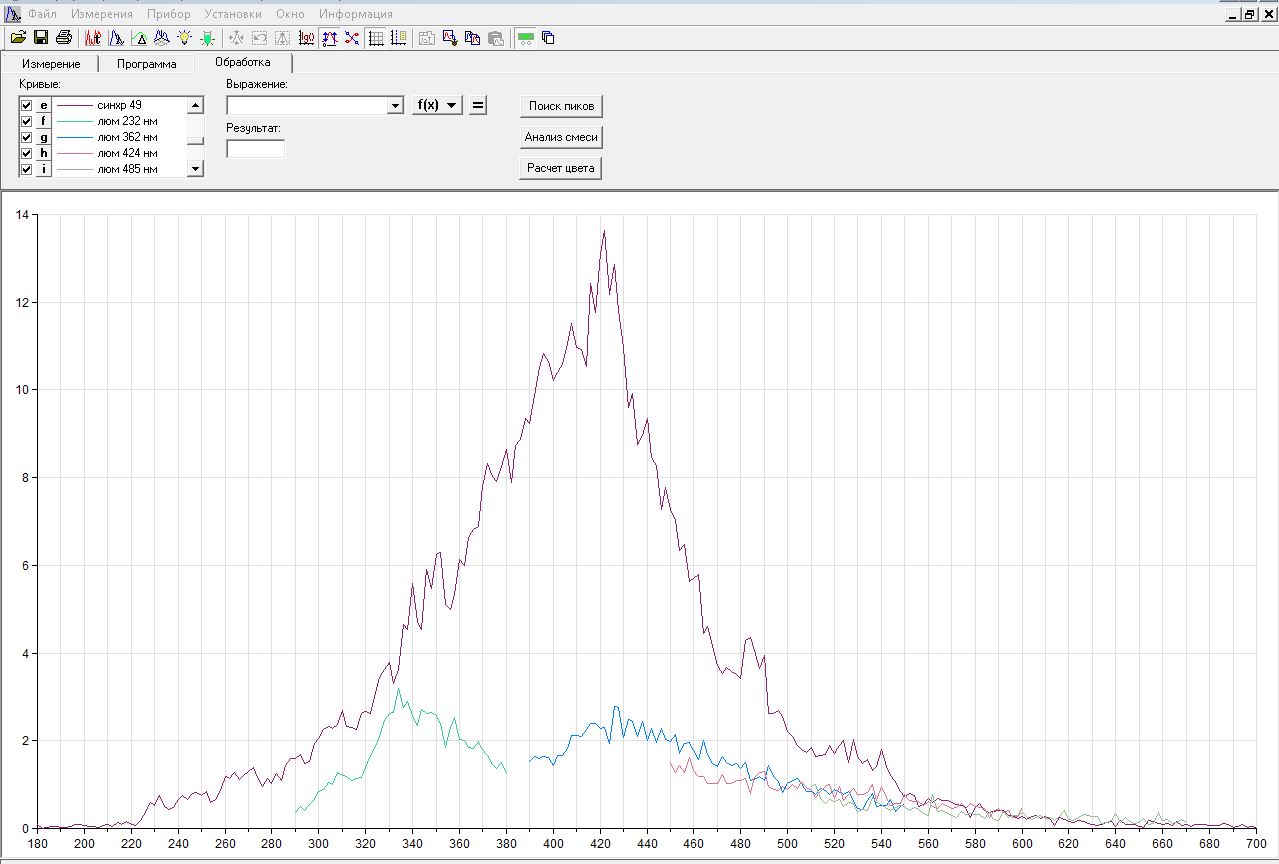

Supplement: S1 Data — (ZIP) [file pone.0267912.s001.zip › Primary Data/Oats/Infected/æ»Ñ¬Γαδ «óßá oáαáaÑ¡¡«ú« 49.JPG]

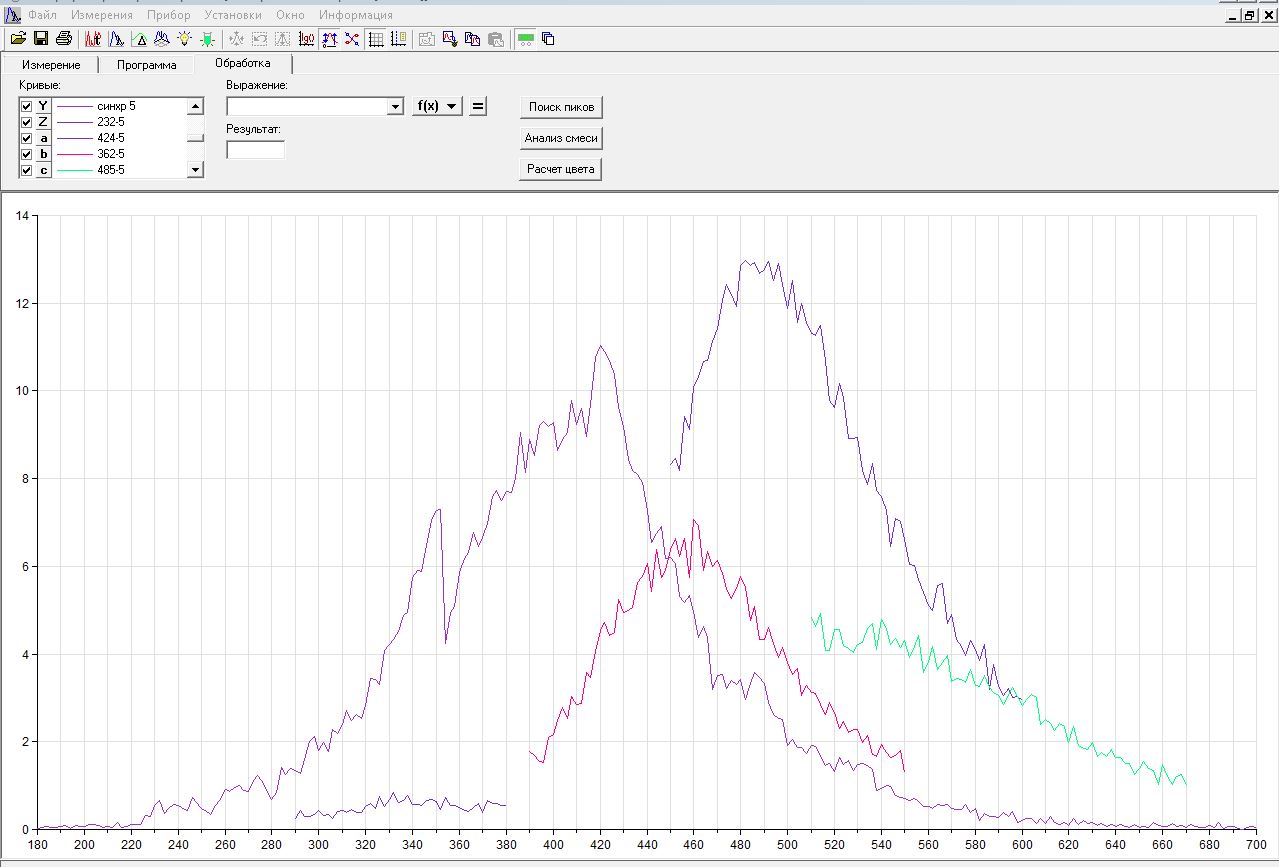

Supplement: S1 Data — (ZIP) [file pone.0267912.s001.zip › Primary Data/Oats/Infected/æ»Ñ¬Γαδ «óßá oáαáaÑ¡¡«ú« 5.JPG]

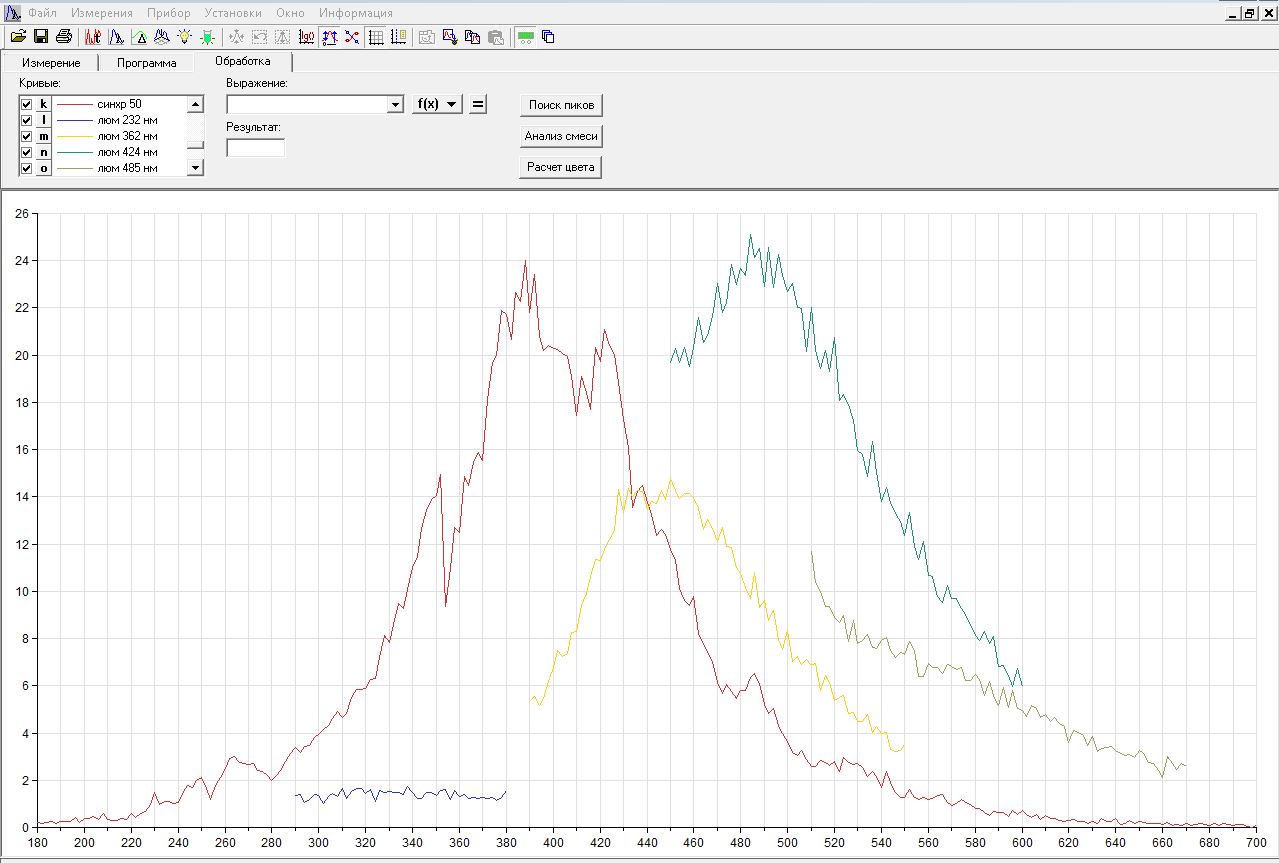

Supplement: S1 Data — (ZIP) [file pone.0267912.s001.zip › Primary Data/Oats/Infected/æ»Ñ¬Γαδ «óßá oáαáaÑ¡¡«ú« 50.JPG]

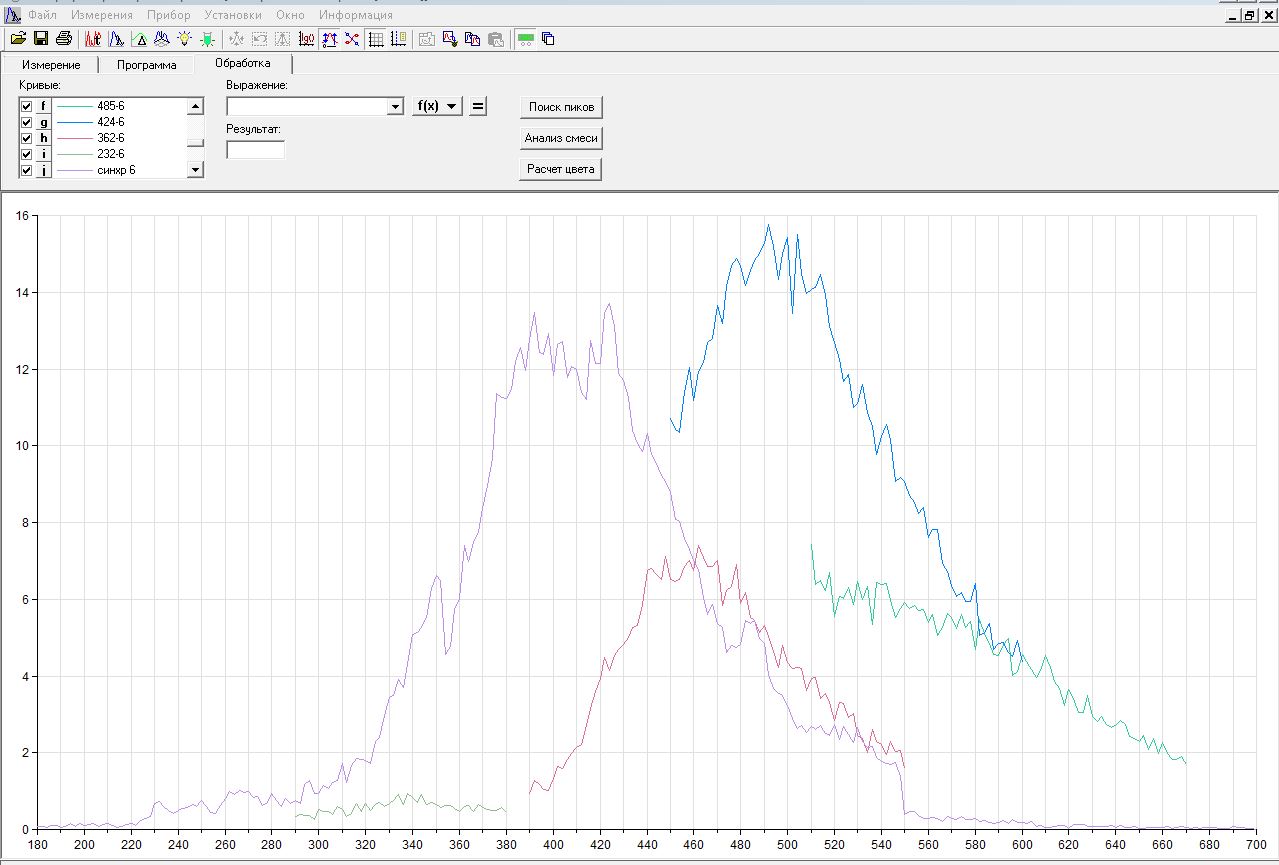

Supplement: S1 Data — (ZIP) [file pone.0267912.s001.zip › Primary Data/Oats/Infected/æ»Ñ¬Γαδ «óßá oáαáaÑ¡¡«ú« 6.JPG]

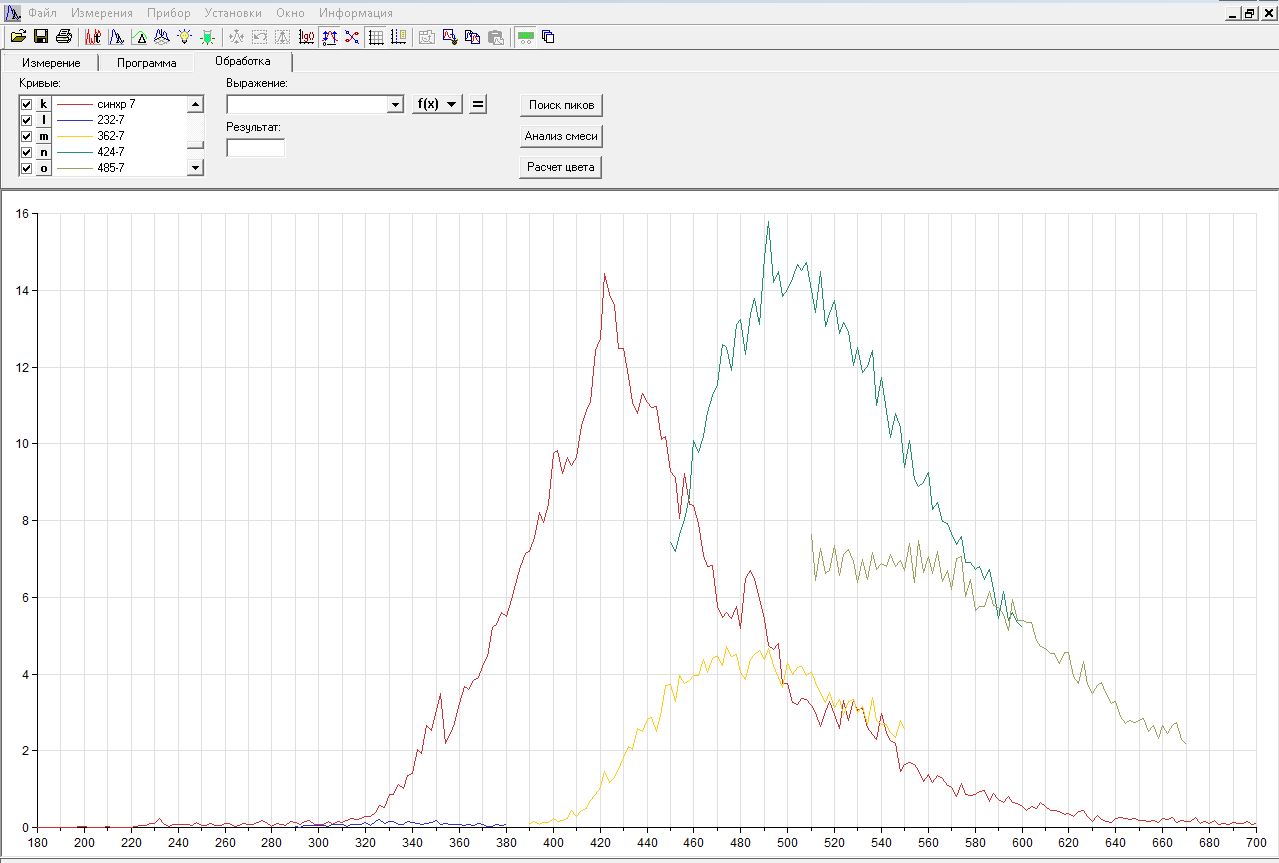

Supplement: S1 Data — (ZIP) [file pone.0267912.s001.zip › Primary Data/Oats/Infected/æ»Ñ¬Γαδ «óßá oáαáaÑ¡¡«ú« 7.JPG]

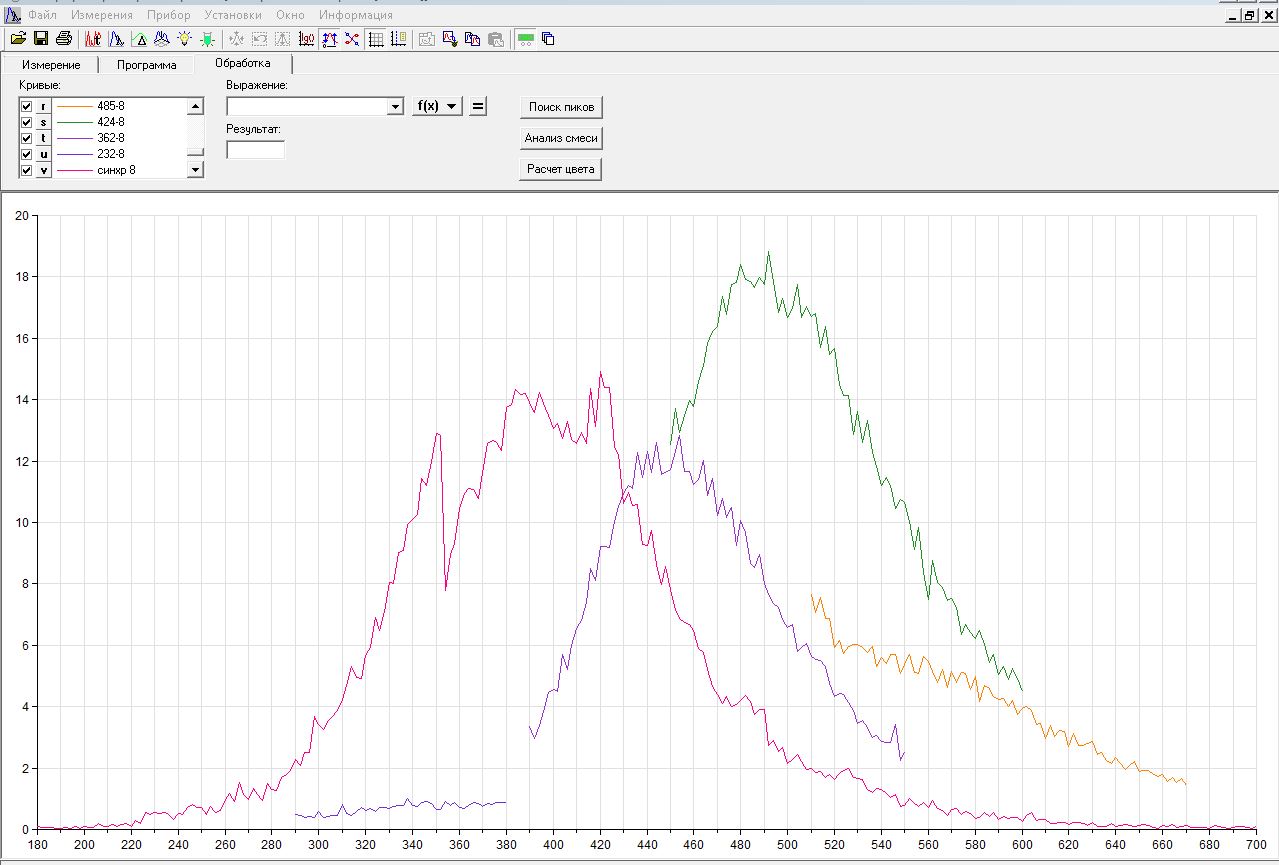

Supplement: S1 Data — (ZIP) [file pone.0267912.s001.zip › Primary Data/Oats/Infected/æ»Ñ¬Γαδ «óßá oáαáaÑ¡¡«ú« 8.JPG]

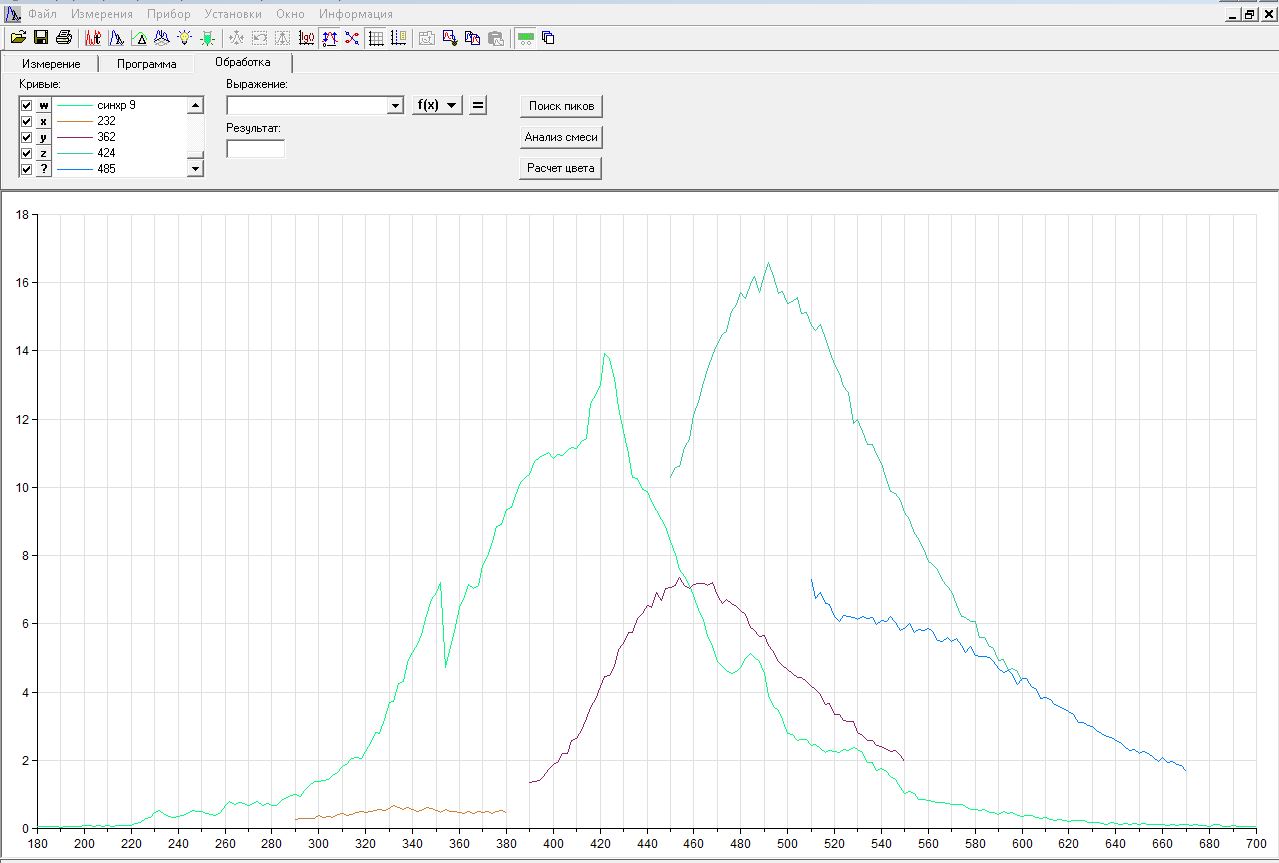

Supplement: S1 Data — (ZIP) [file pone.0267912.s001.zip › Primary Data/Oats/Infected/æ»Ñ¬Γαδ «óßá oáαáaÑ¡¡«ú« 9.JPG]

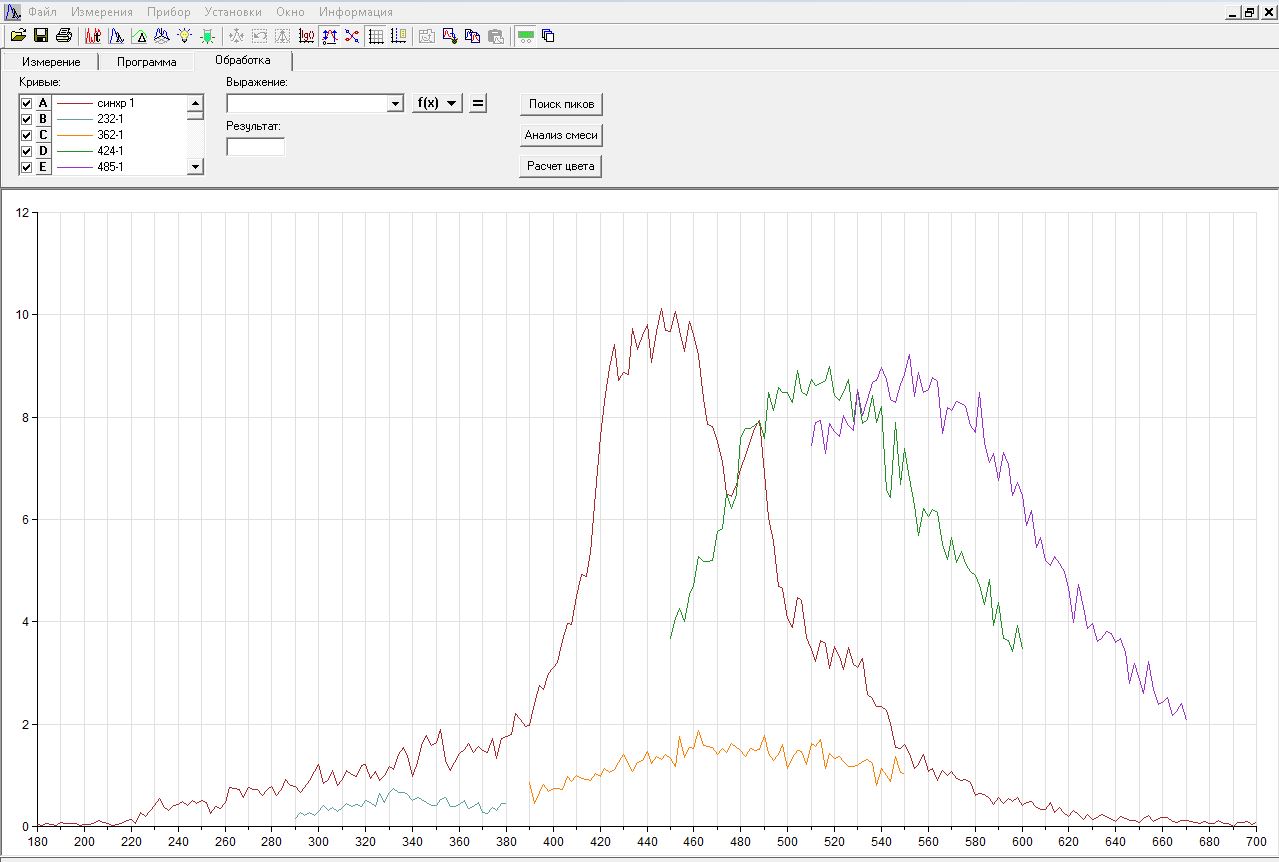

Supplement: S1 Data — (ZIP) [file pone.0267912.s001.zip › Primary Data/Oats/Uninfected/æ»Ñ¬Γαδ «óßá ¡Ñ oáαáaÑ¡¡«ú« 1.JPG]

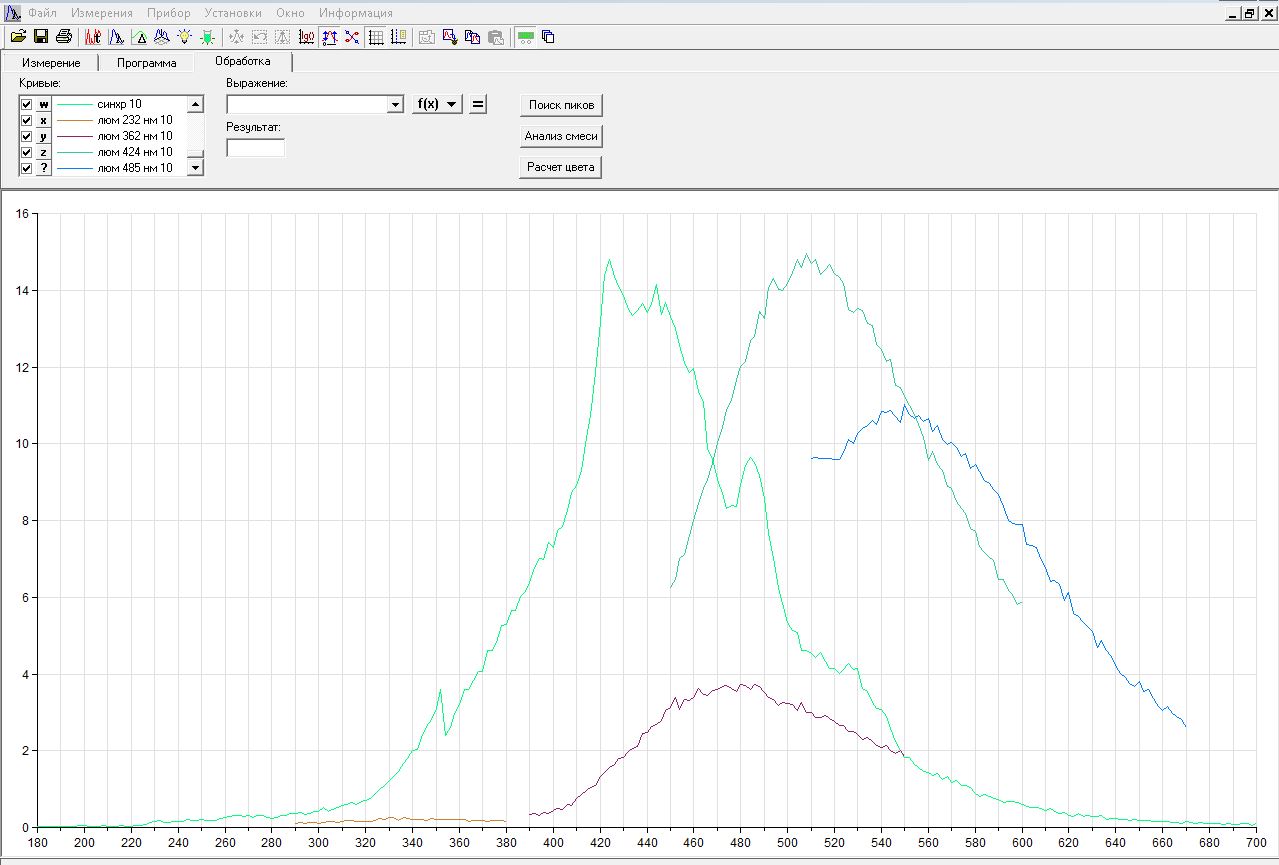

Supplement: S1 Data — (ZIP) [file pone.0267912.s001.zip › Primary Data/Oats/Uninfected/æ»Ñ¬Γαδ «óßá ¡Ñ oáαáaÑ¡¡«ú« 10.JPG]

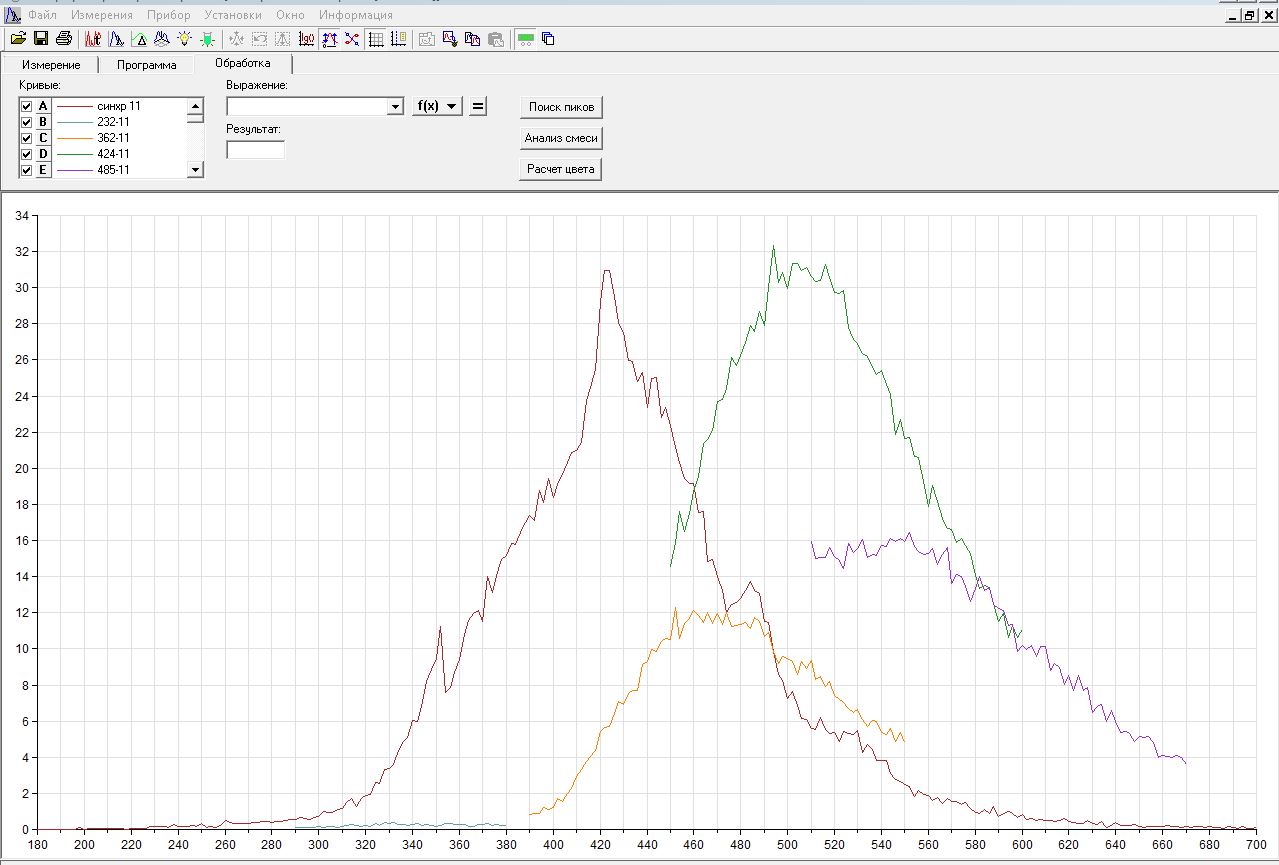

Supplement: S1 Data — (ZIP) [file pone.0267912.s001.zip › Primary Data/Oats/Uninfected/æ»Ñ¬Γαδ «óßá ¡Ñ oáαáaÑ¡¡«ú« 11.JPG]

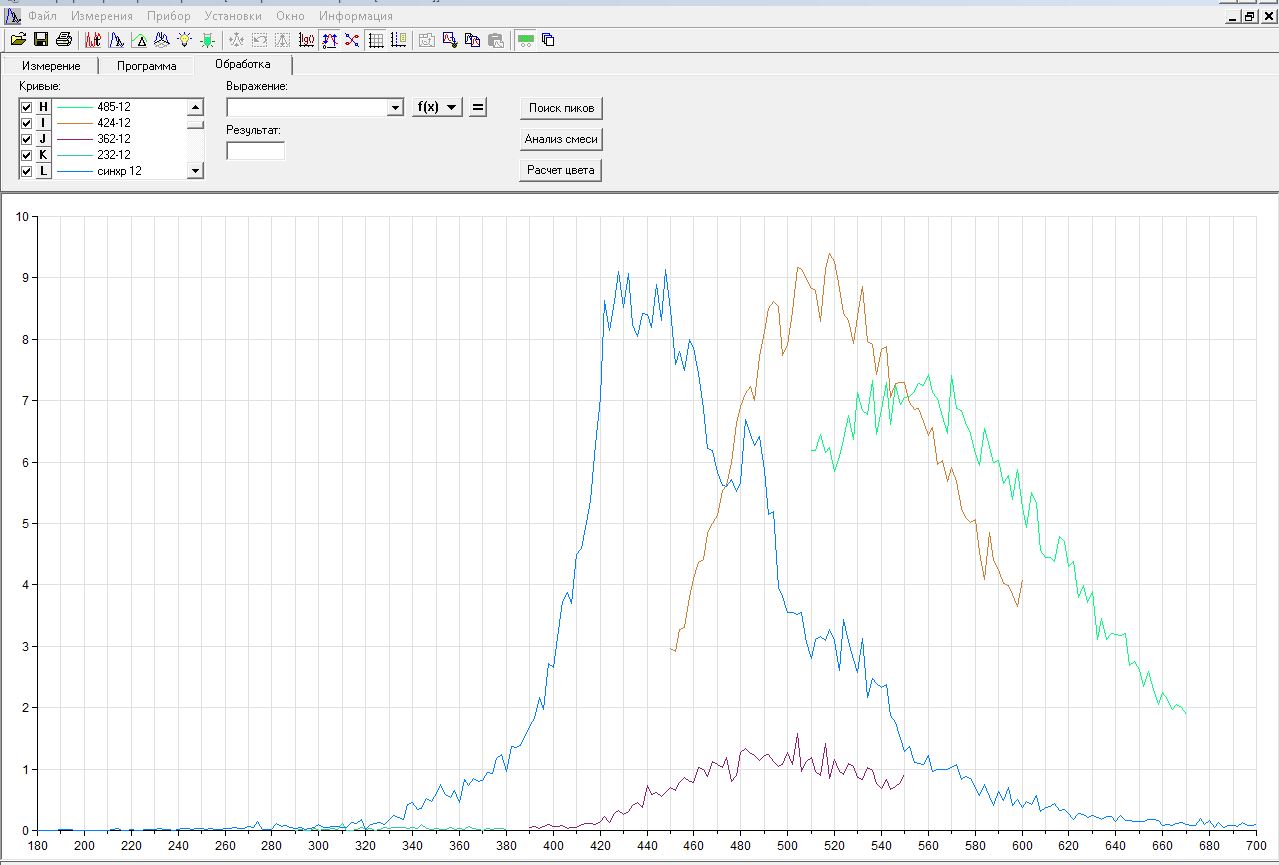

Supplement: S1 Data — (ZIP) [file pone.0267912.s001.zip › Primary Data/Oats/Uninfected/æ»Ñ¬Γαδ «óßá ¡Ñ oáαáaÑ¡¡«ú« 12.JPG]

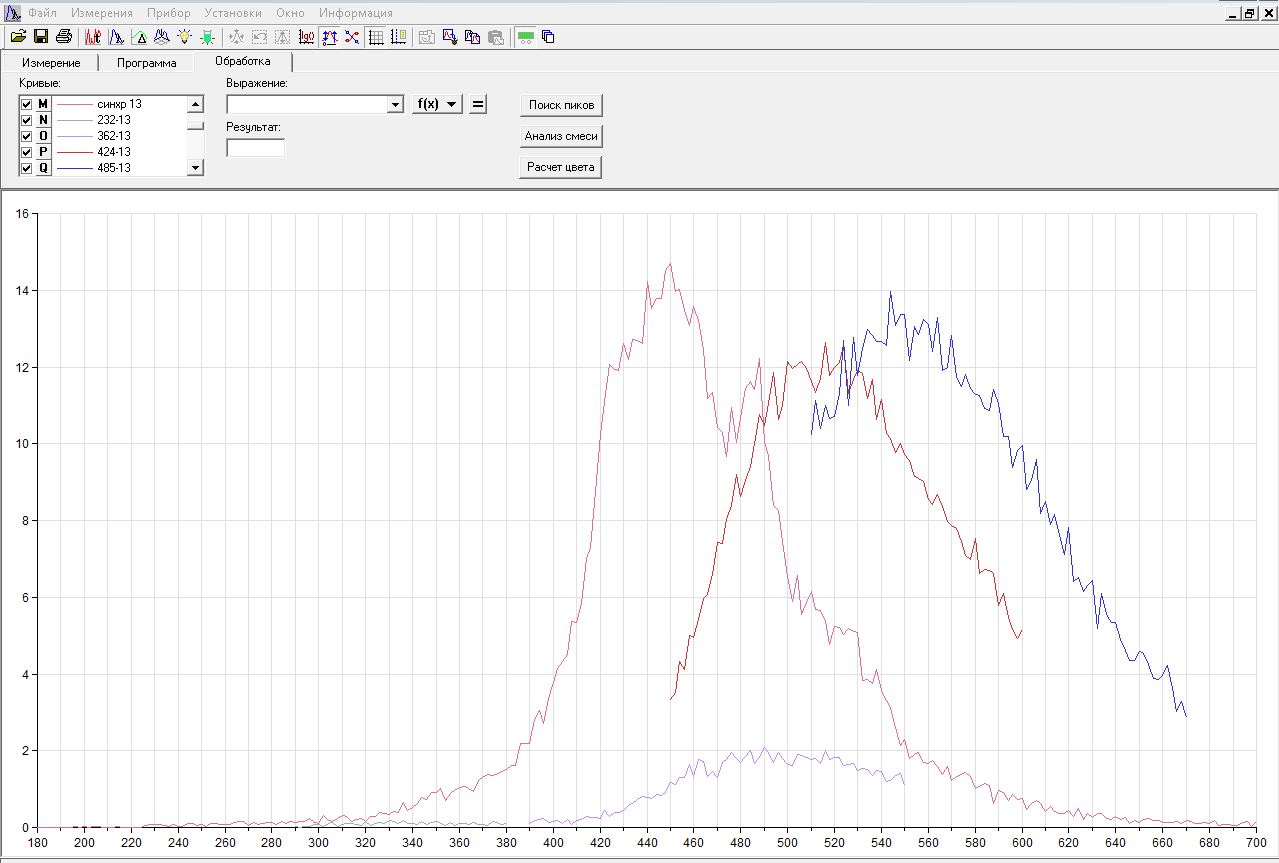

Supplement: S1 Data — (ZIP) [file pone.0267912.s001.zip › Primary Data/Oats/Uninfected/æ»Ñ¬Γαδ «óßá ¡Ñ oáαáaÑ¡¡«ú« 13.JPG]

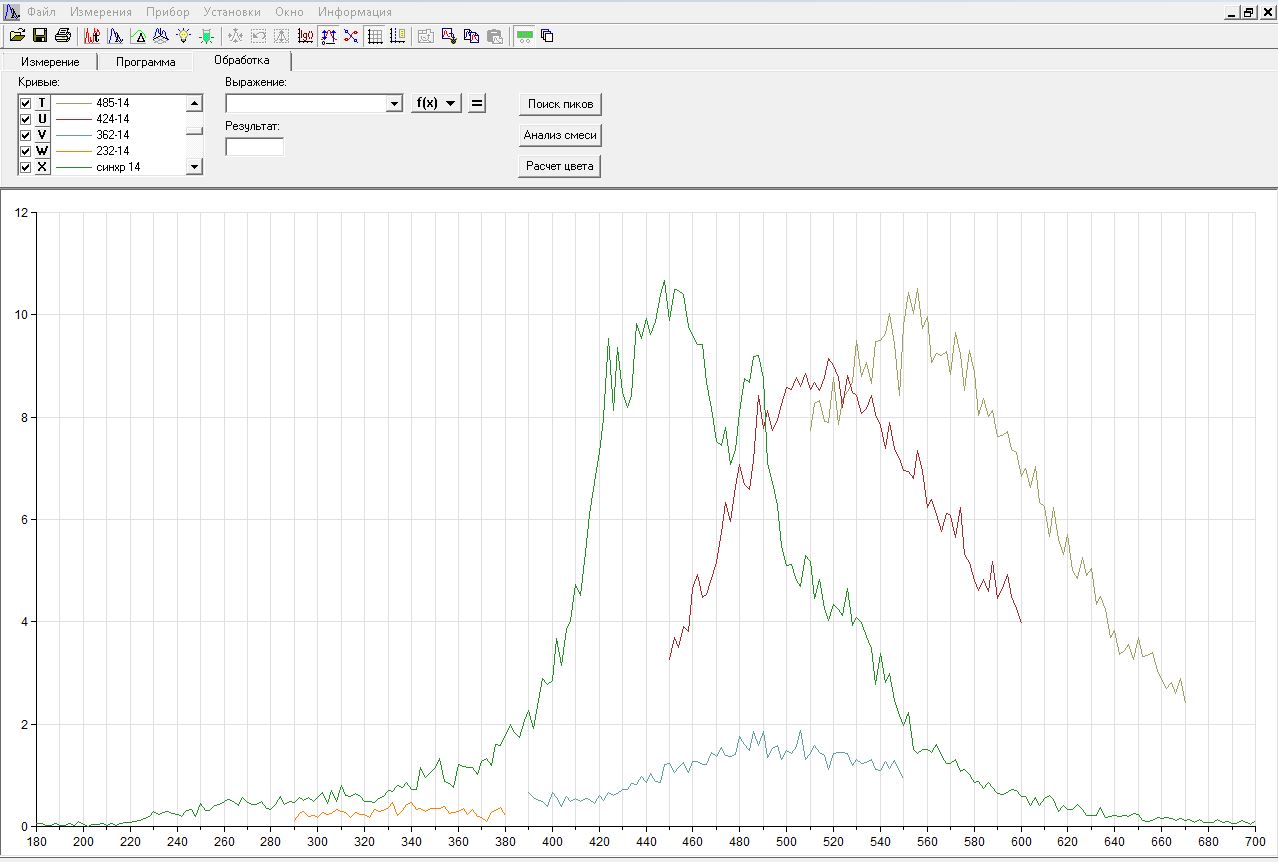

Supplement: S1 Data — (ZIP) [file pone.0267912.s001.zip › Primary Data/Oats/Uninfected/æ»Ñ¬Γαδ «óßá ¡Ñ oáαáaÑ¡¡«ú« 14.JPG]

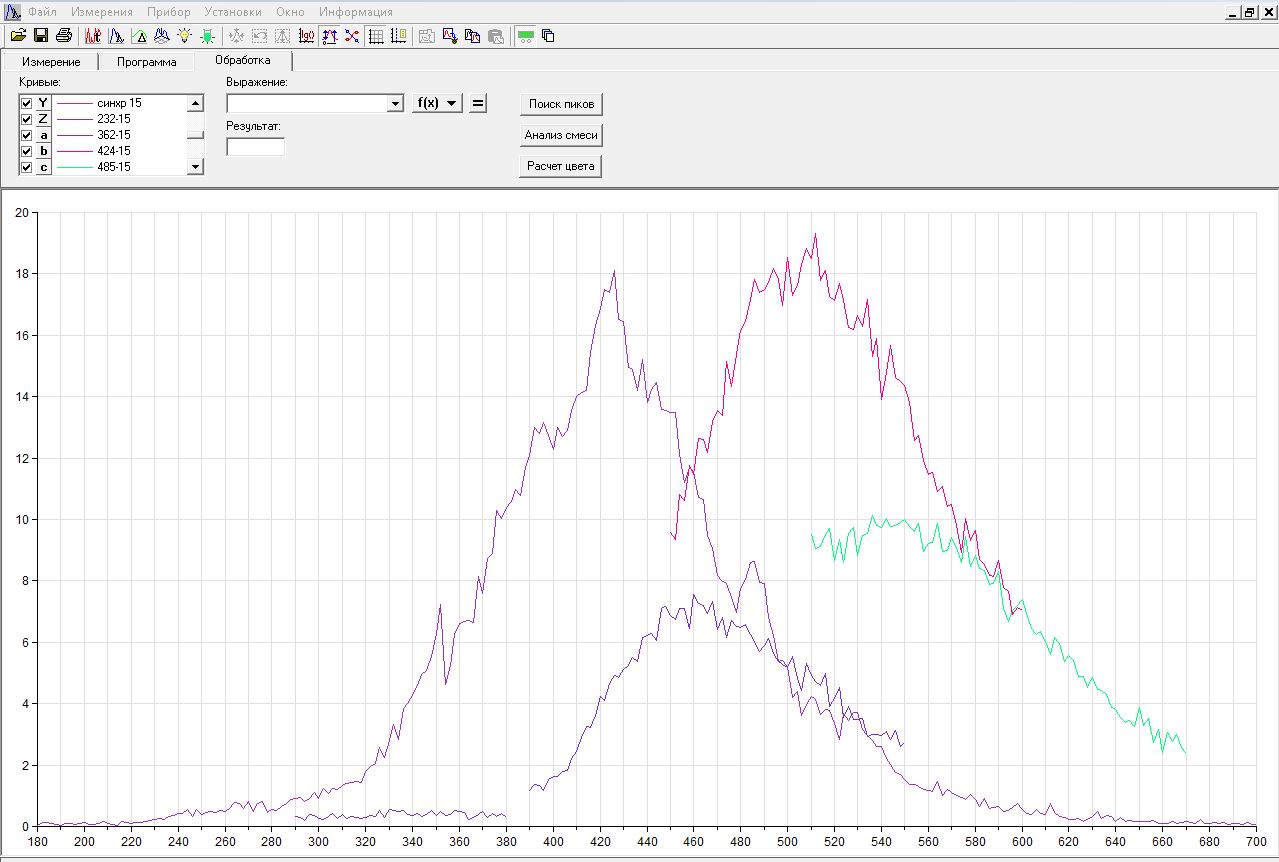

Supplement: S1 Data — (ZIP) [file pone.0267912.s001.zip › Primary Data/Oats/Uninfected/æ»Ñ¬Γαδ «óßá ¡Ñ oáαáaÑ¡¡«ú« 15.JPG]

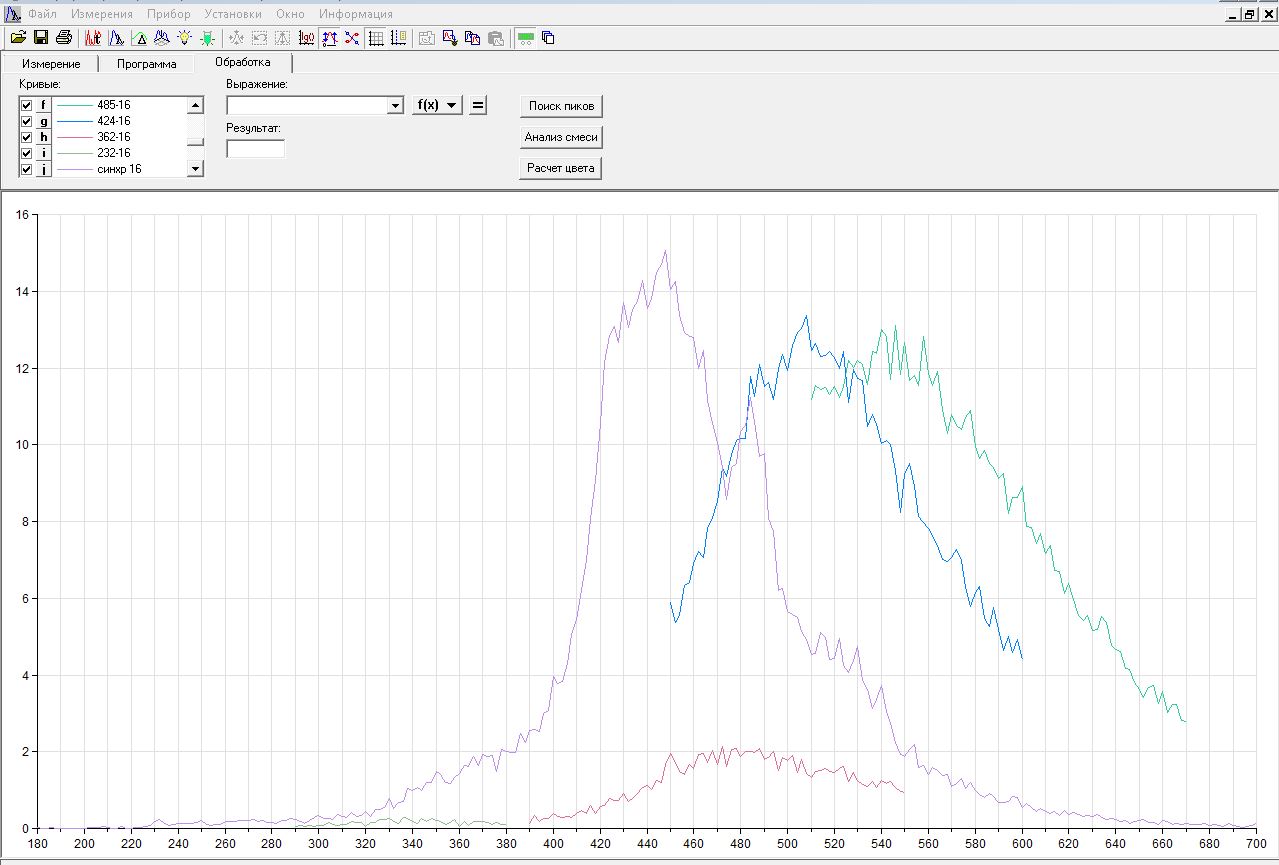

Supplement: S1 Data — (ZIP) [file pone.0267912.s001.zip › Primary Data/Oats/Uninfected/æ»Ñ¬Γαδ «óßá ¡Ñ oáαáaÑ¡¡«ú« 16.JPG]

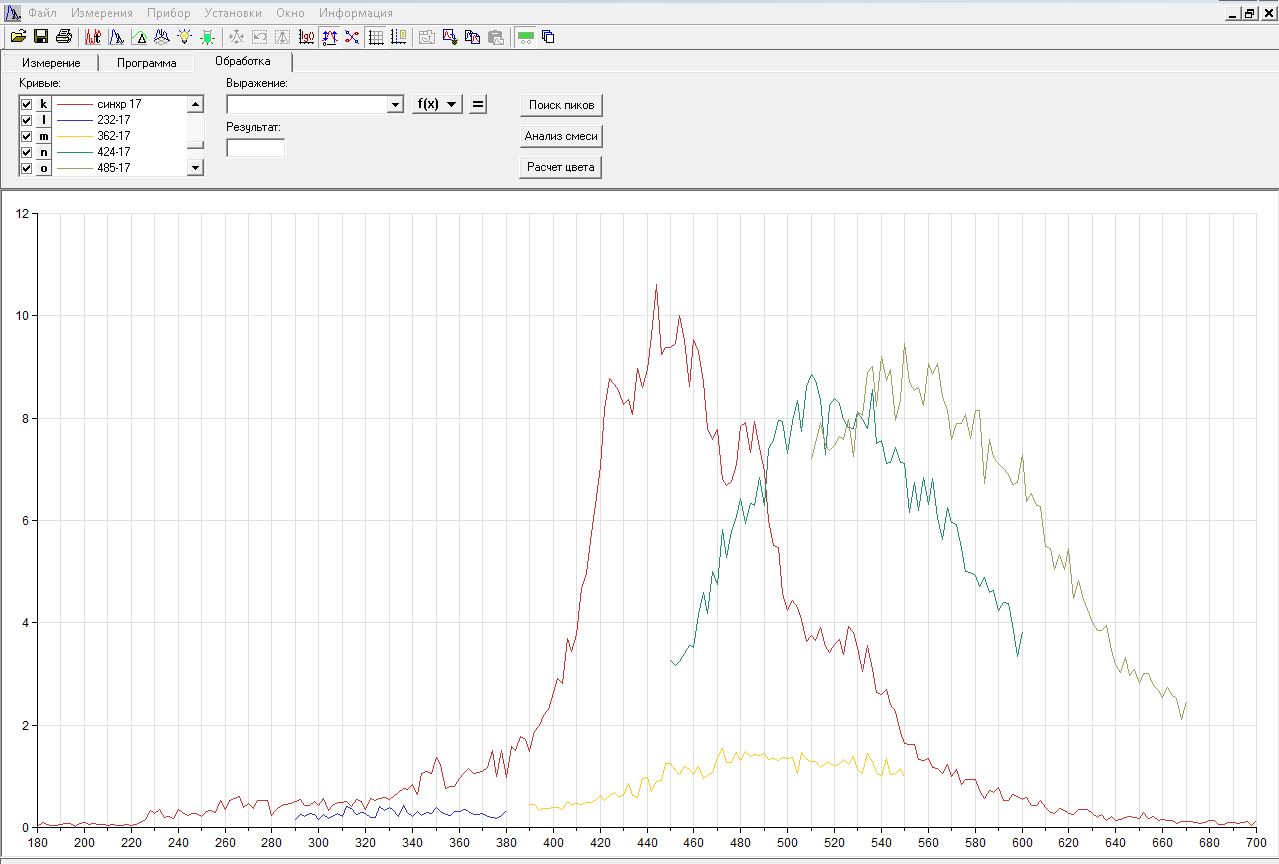

Supplement: S1 Data — (ZIP) [file pone.0267912.s001.zip › Primary Data/Oats/Uninfected/æ»Ñ¬Γαδ «óßá ¡Ñ oáαáaÑ¡¡«ú« 17.JPG]

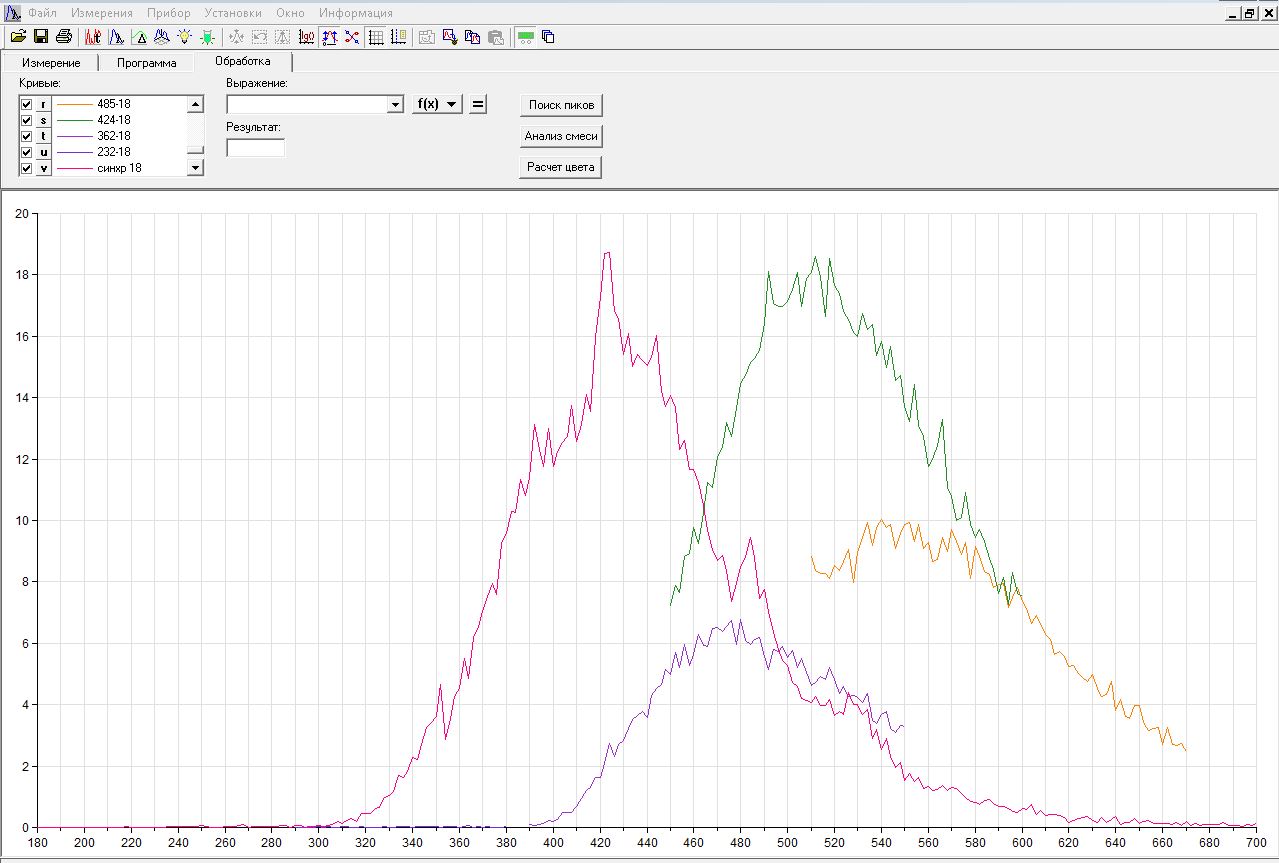

Supplement: S1 Data — (ZIP) [file pone.0267912.s001.zip › Primary Data/Oats/Uninfected/æ»Ñ¬Γαδ «óßá ¡Ñ oáαáaÑ¡¡«ú« 18.JPG]

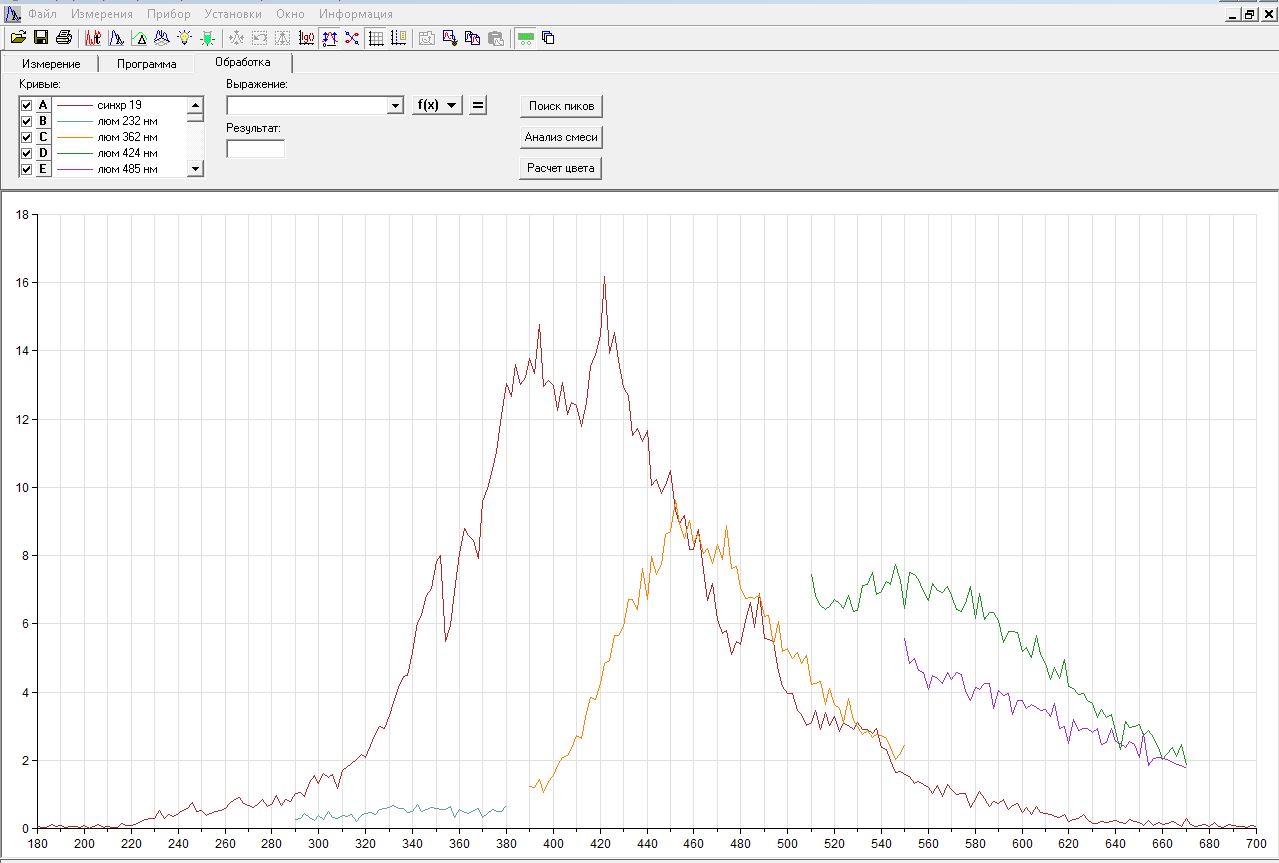

Supplement: S1 Data — (ZIP) [file pone.0267912.s001.zip › Primary Data/Oats/Uninfected/æ»Ñ¬Γαδ «óßá ¡Ñ oáαáaÑ¡¡«ú« 19.JPG]

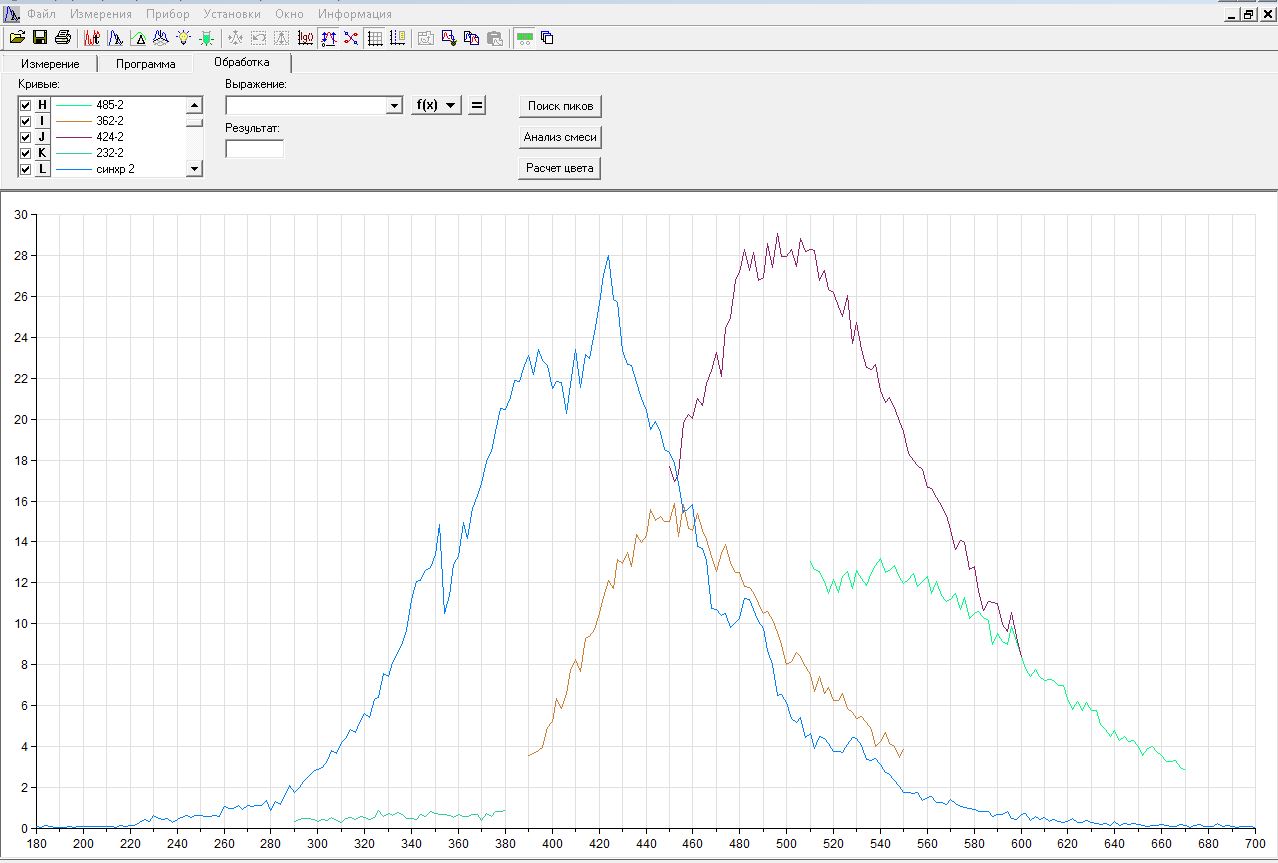

Supplement: S1 Data — (ZIP) [file pone.0267912.s001.zip › Primary Data/Oats/Uninfected/æ»Ñ¬Γαδ «óßá ¡Ñ oáαáaÑ¡¡«ú« 2.JPG]

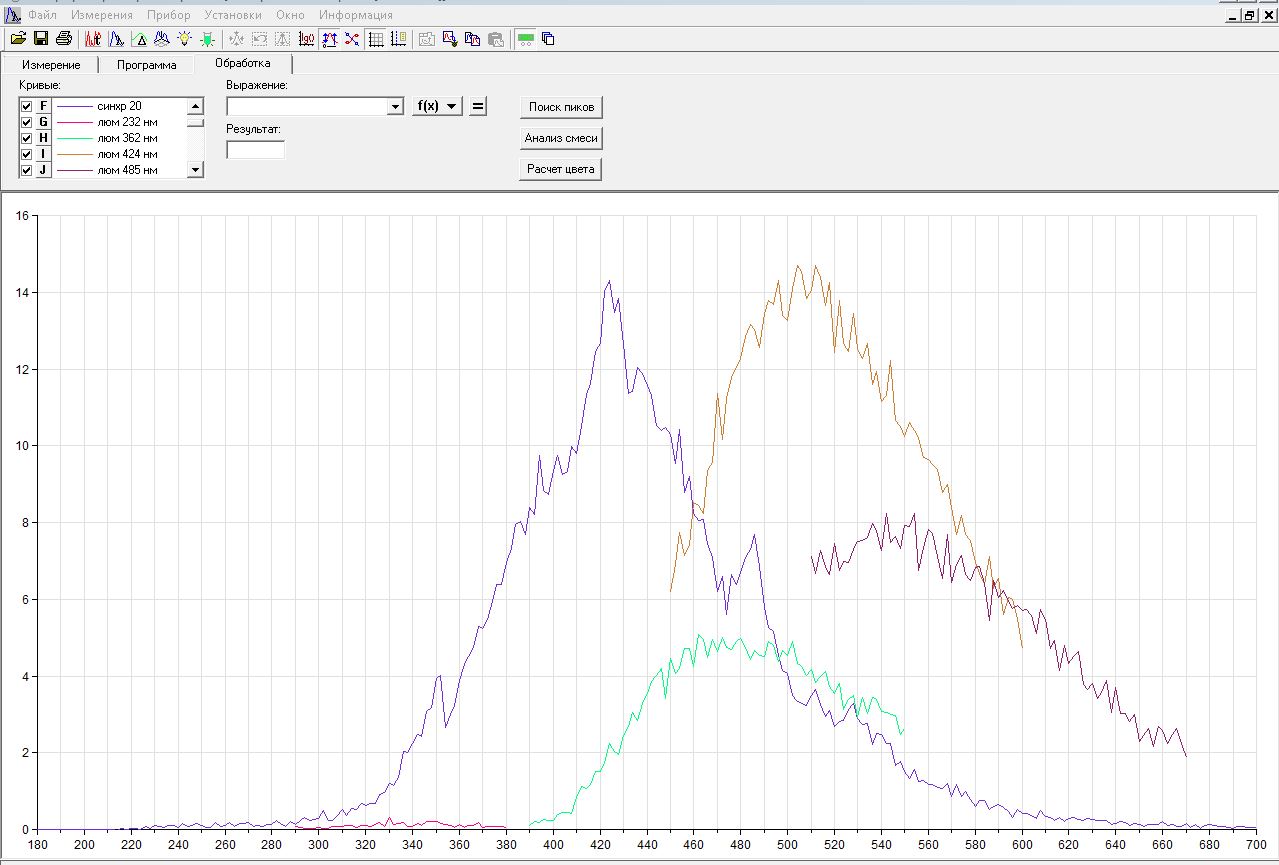

Supplement: S1 Data — (ZIP) [file pone.0267912.s001.zip › Primary Data/Oats/Uninfected/æ»Ñ¬Γαδ «óßá ¡Ñ oáαáaÑ¡¡«ú« 20.JPG]

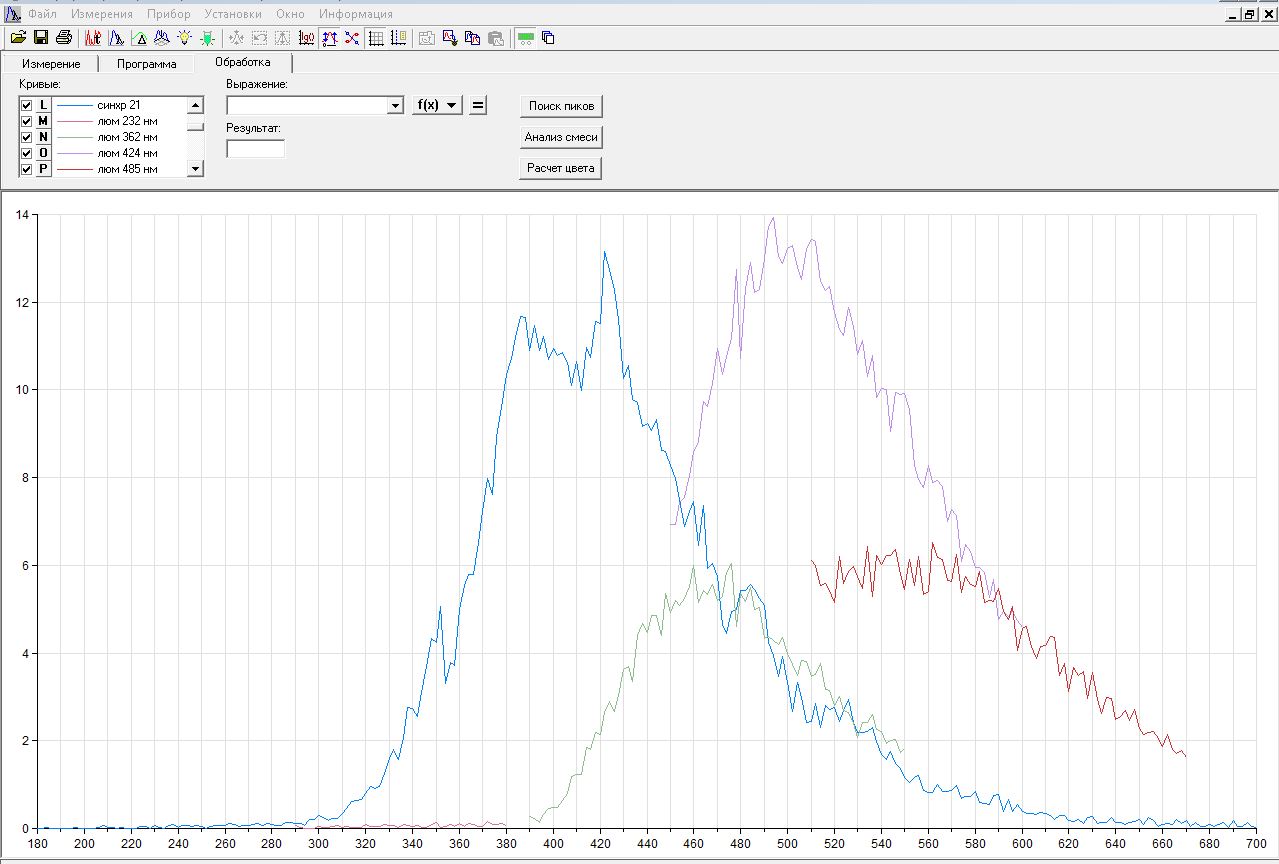

Supplement: S1 Data — (ZIP) [file pone.0267912.s001.zip › Primary Data/Oats/Uninfected/æ»Ñ¬Γαδ «óßá ¡Ñ oáαáaÑ¡¡«ú« 21.JPG]

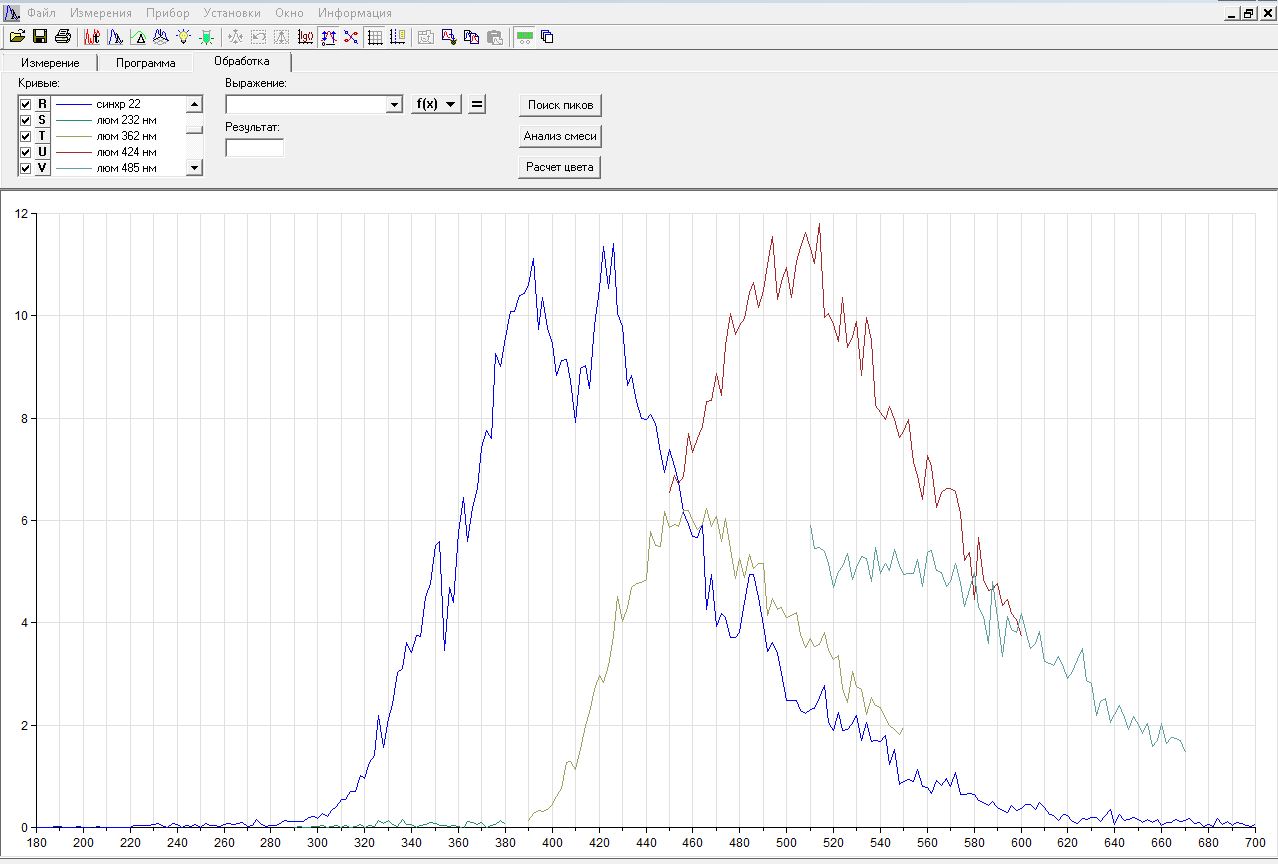

Supplement: S1 Data — (ZIP) [file pone.0267912.s001.zip › Primary Data/Oats/Uninfected/æ»Ñ¬Γαδ «óßá ¡Ñ oáαáaÑ¡¡«ú« 22.JPG]

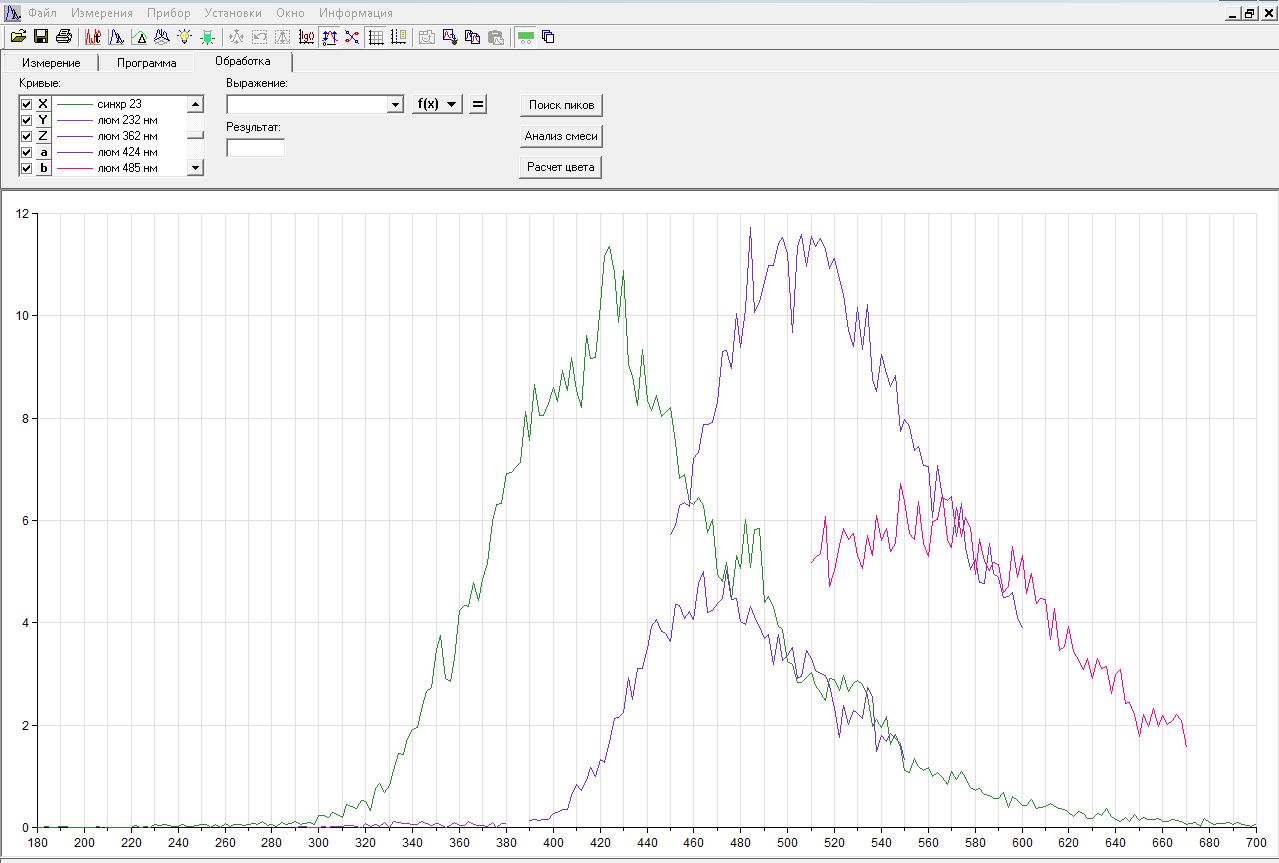

Supplement: S1 Data — (ZIP) [file pone.0267912.s001.zip › Primary Data/Oats/Uninfected/æ»Ñ¬Γαδ «óßá ¡Ñ oáαáaÑ¡¡«ú« 23.JPG]

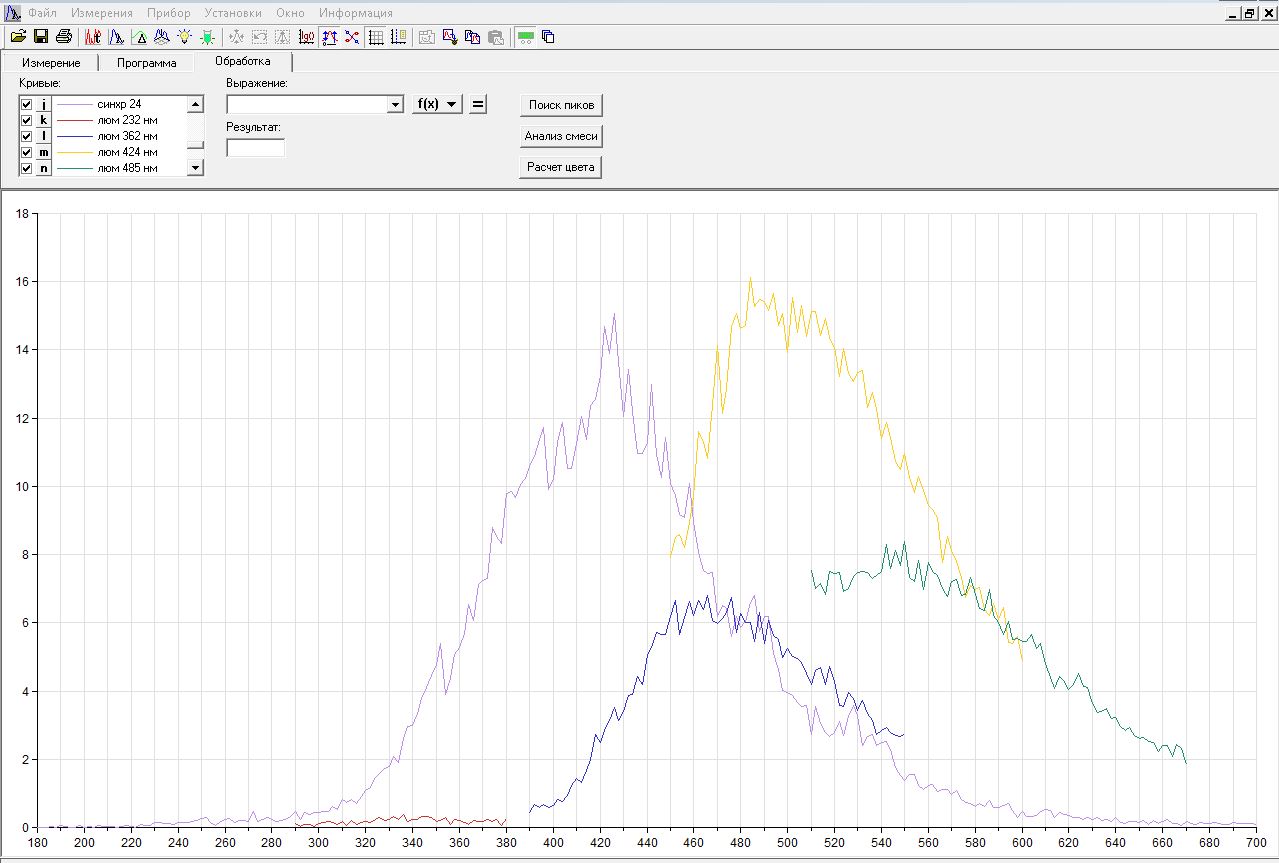

Supplement: S1 Data — (ZIP) [file pone.0267912.s001.zip › Primary Data/Oats/Uninfected/æ»Ñ¬Γαδ «óßá ¡Ñ oáαáaÑ¡¡«ú« 24.JPG]

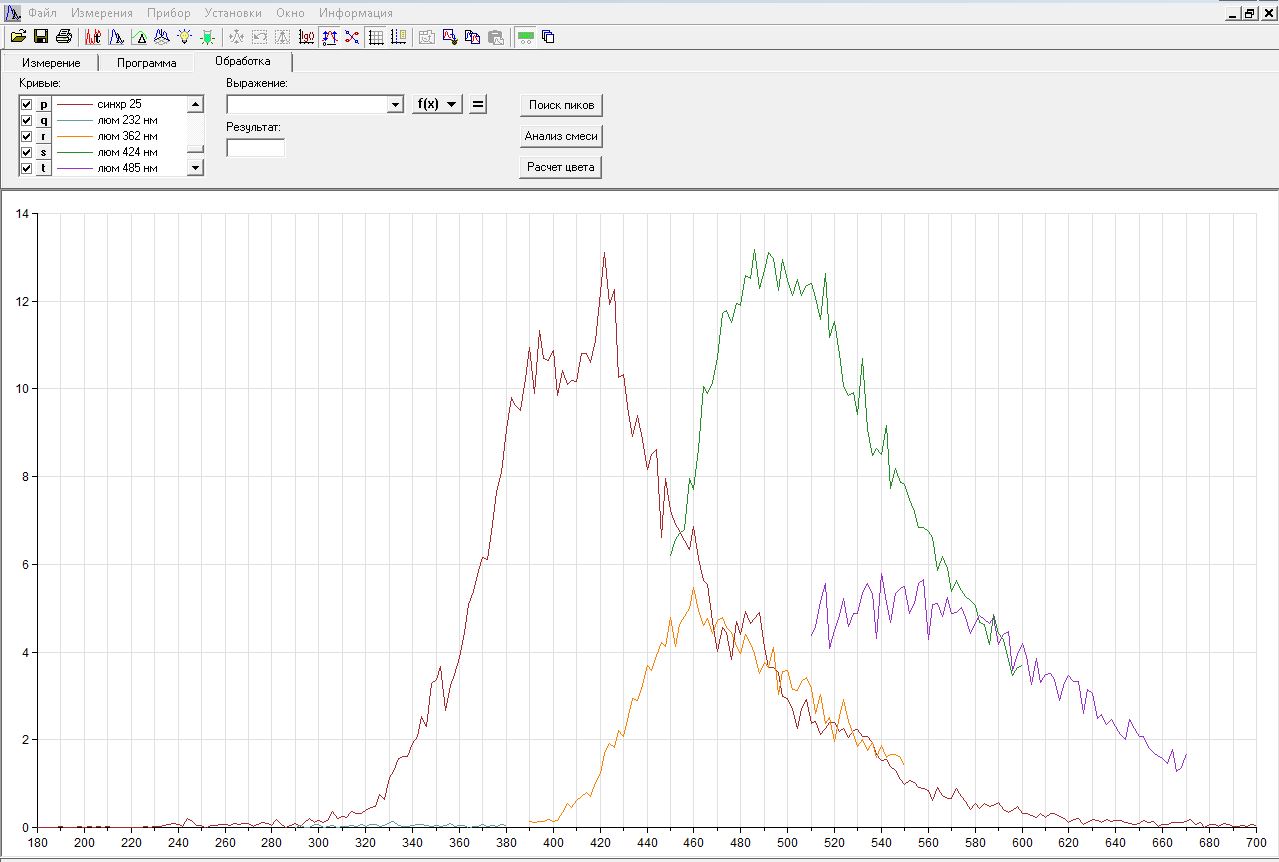

Supplement: S1 Data — (ZIP) [file pone.0267912.s001.zip › Primary Data/Oats/Uninfected/æ»Ñ¬Γαδ «óßá ¡Ñ oáαáaÑ¡¡«ú« 25.JPG]

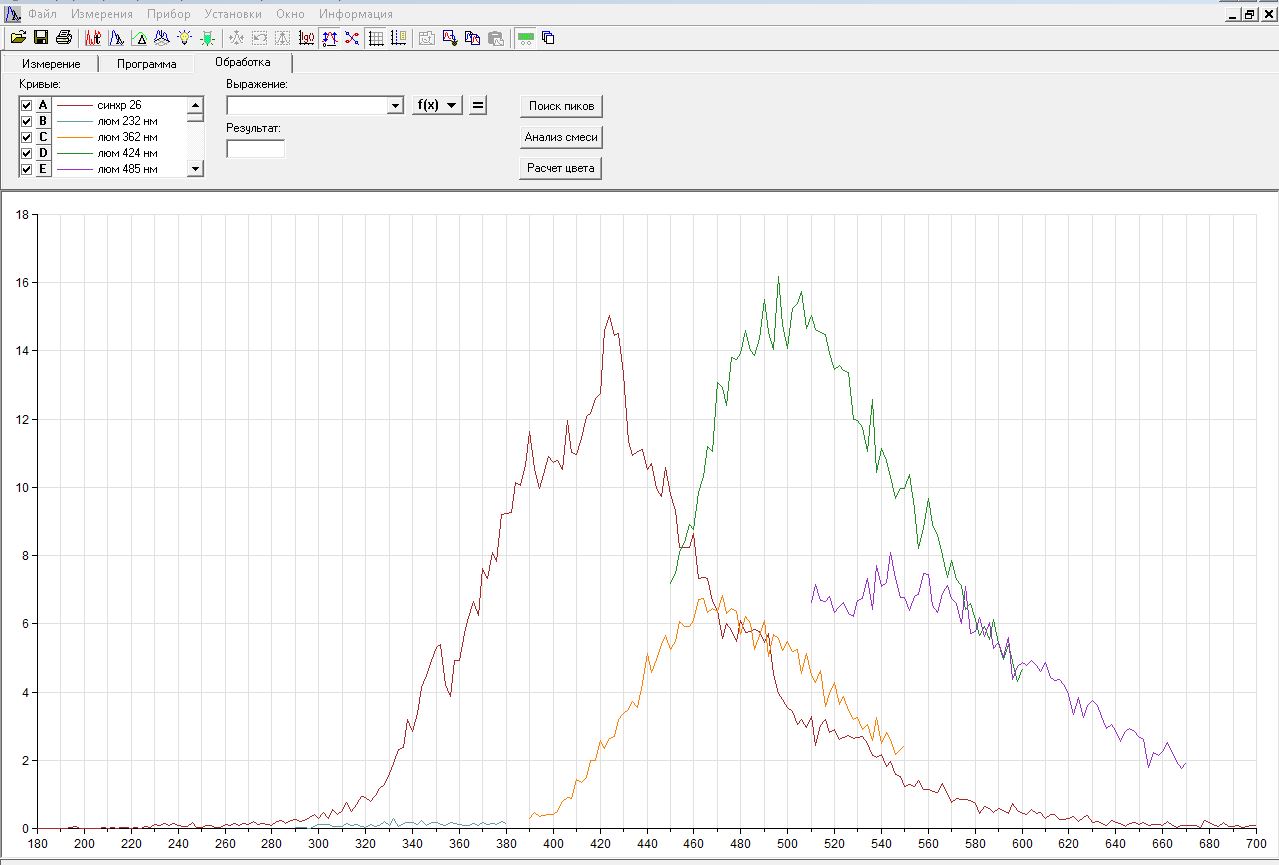

Supplement: S1 Data — (ZIP) [file pone.0267912.s001.zip › Primary Data/Oats/Uninfected/æ»Ñ¬Γαδ «óßá ¡Ñ oáαáaÑ¡¡«ú« 26.JPG]

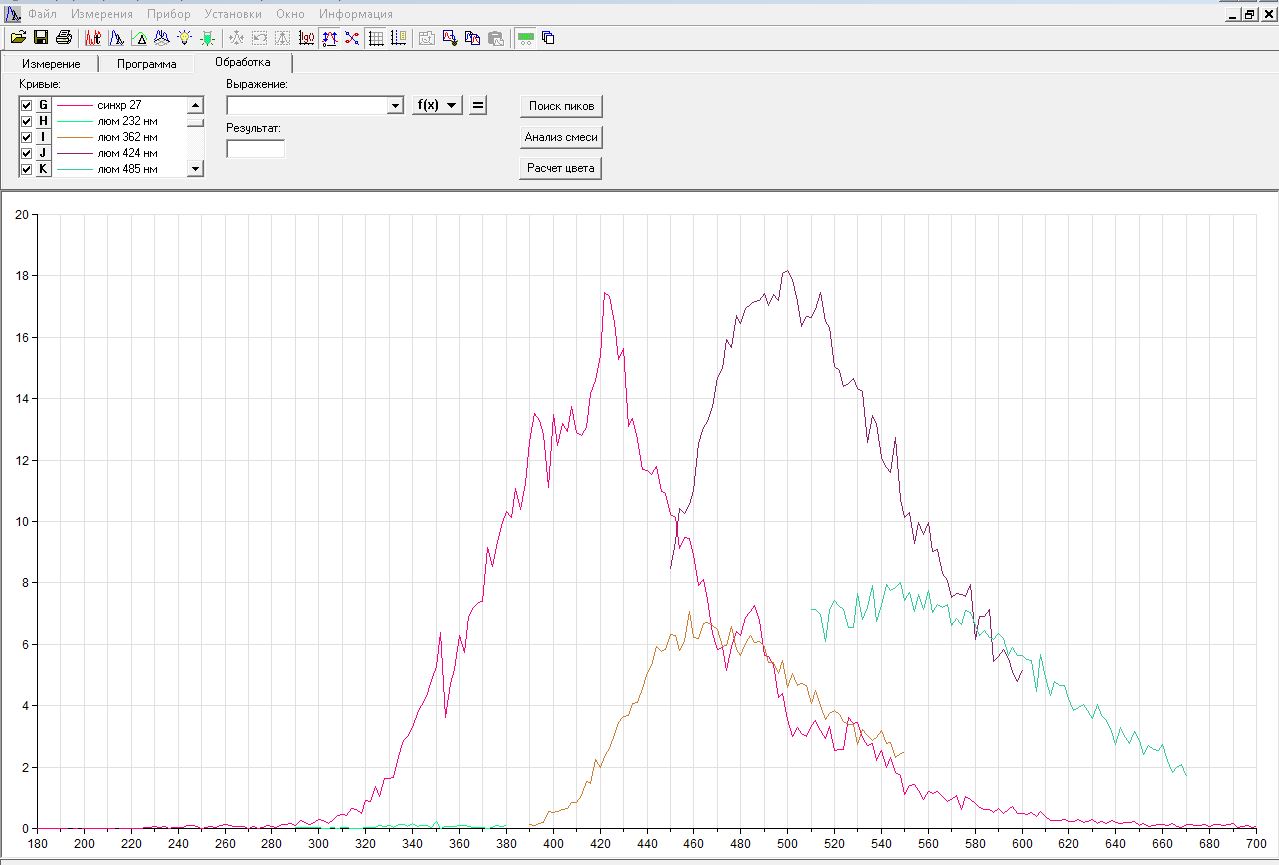

Supplement: S1 Data — (ZIP) [file pone.0267912.s001.zip › Primary Data/Oats/Uninfected/æ»Ñ¬Γαδ «óßá ¡Ñ oáαáaÑ¡¡«ú« 27.JPG]

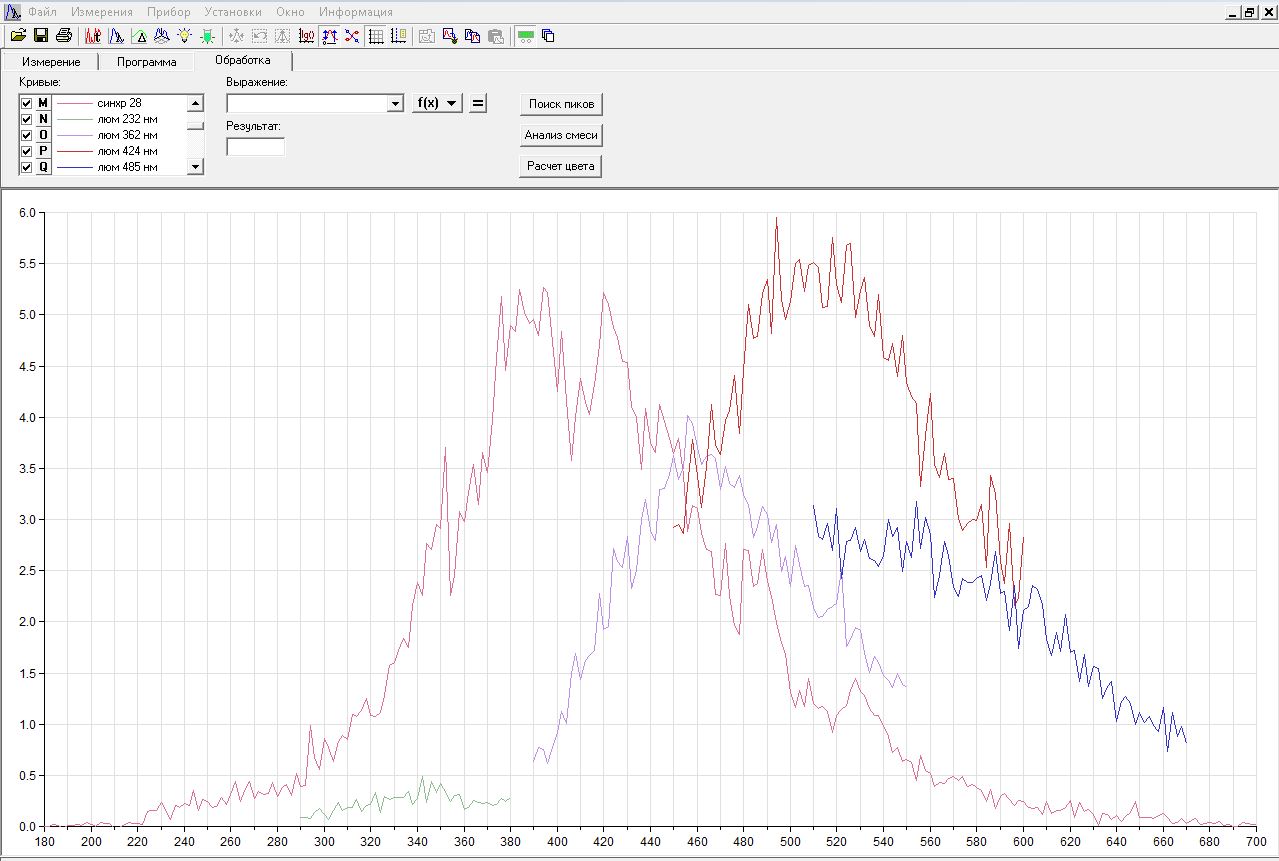

Supplement: S1 Data — (ZIP) [file pone.0267912.s001.zip › Primary Data/Oats/Uninfected/æ»Ñ¬Γαδ «óßá ¡Ñ oáαáaÑ¡¡«ú« 28.JPG]

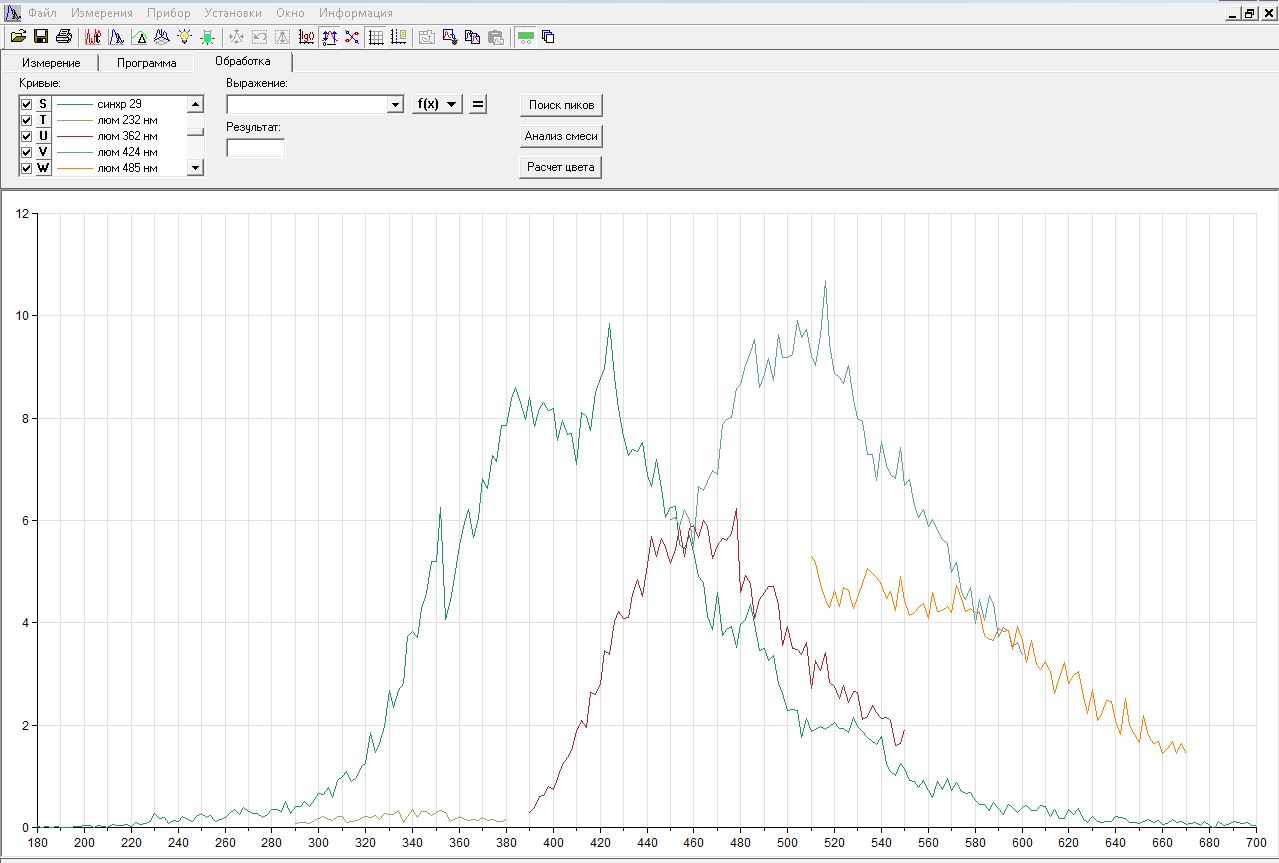

Supplement: S1 Data — (ZIP) [file pone.0267912.s001.zip › Primary Data/Oats/Uninfected/æ»Ñ¬Γαδ «óßá ¡Ñ oáαáaÑ¡¡«ú« 29.JPG]

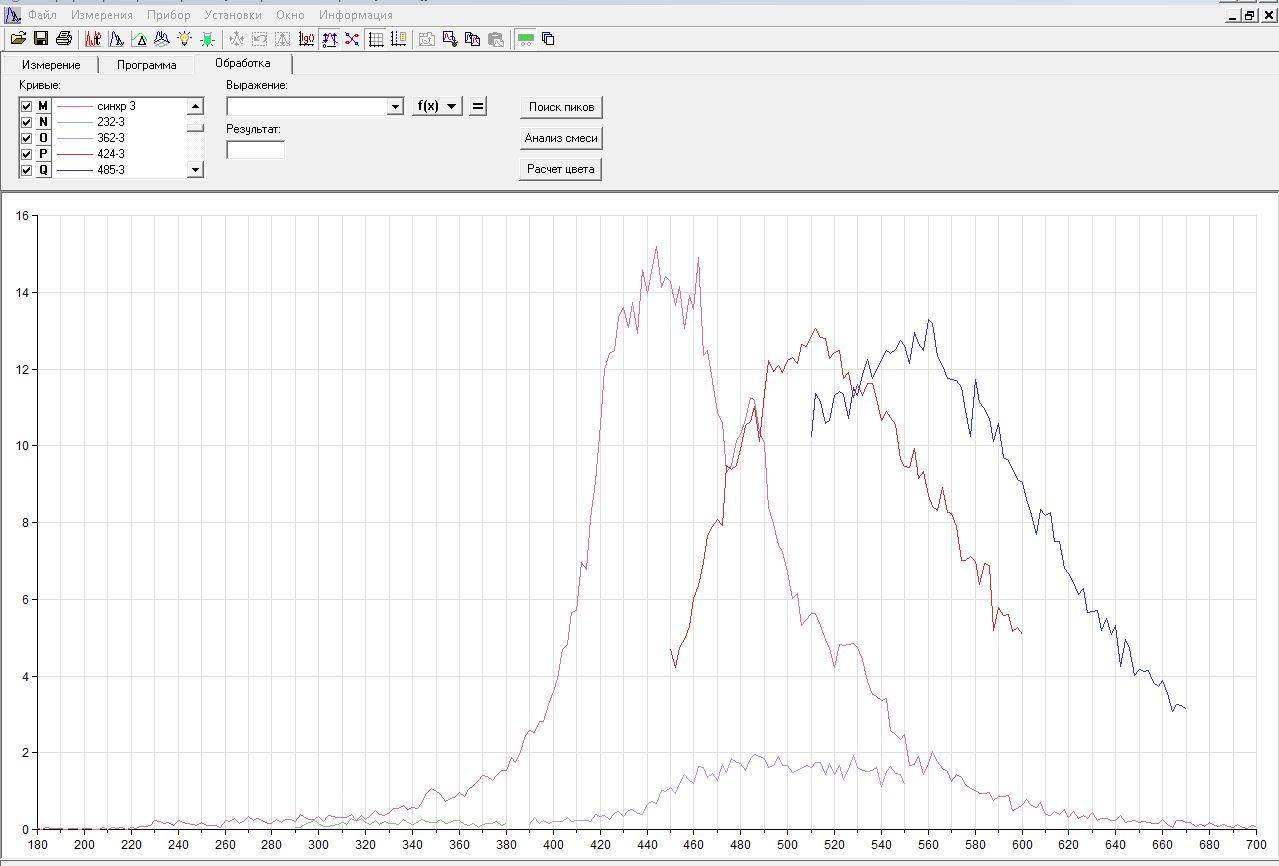

Supplement: S1 Data — (ZIP) [file pone.0267912.s001.zip › Primary Data/Oats/Uninfected/æ»Ñ¬Γαδ «óßá ¡Ñ oáαáaÑ¡¡«ú« 3.JPG]

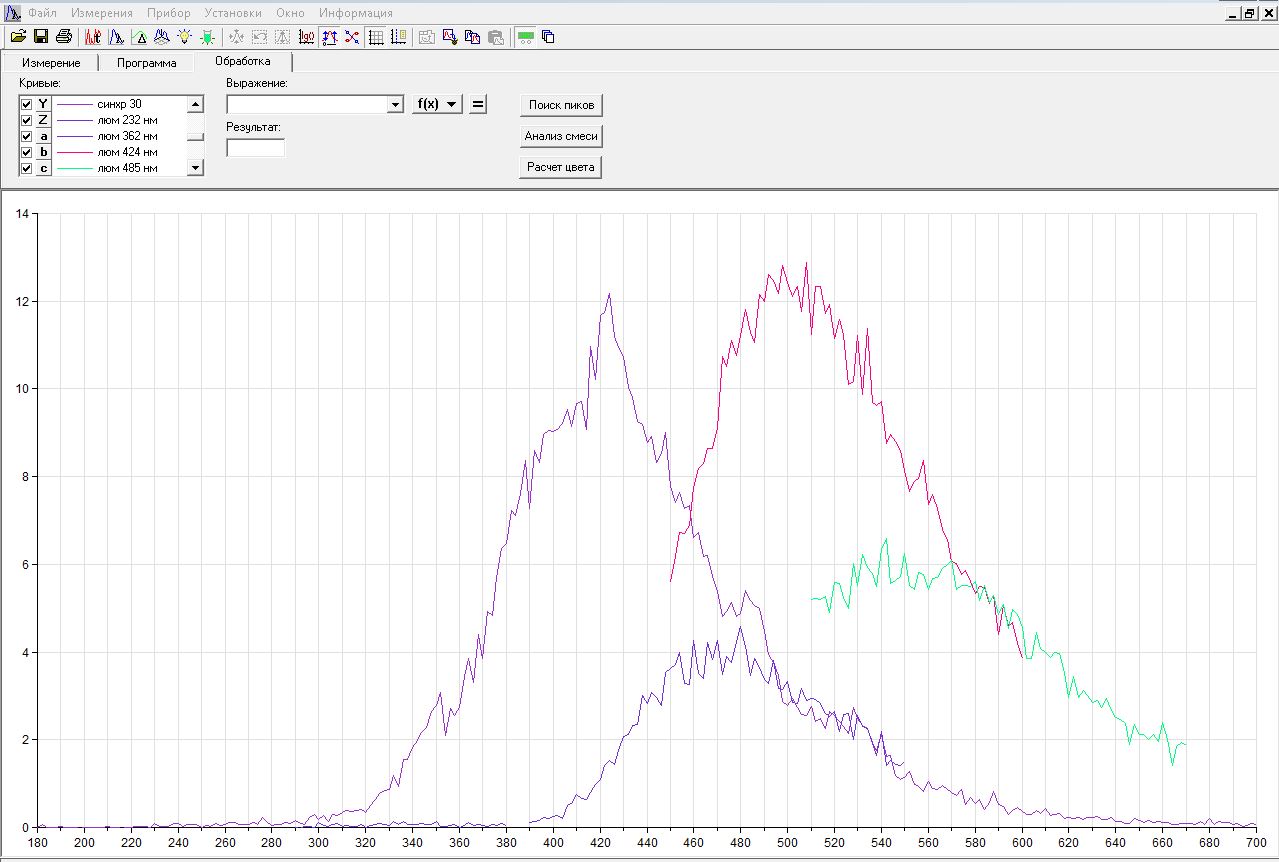

Supplement: S1 Data — (ZIP) [file pone.0267912.s001.zip › Primary Data/Oats/Uninfected/æ»Ñ¬Γαδ «óßá ¡Ñ oáαáaÑ¡¡«ú« 30.JPG]

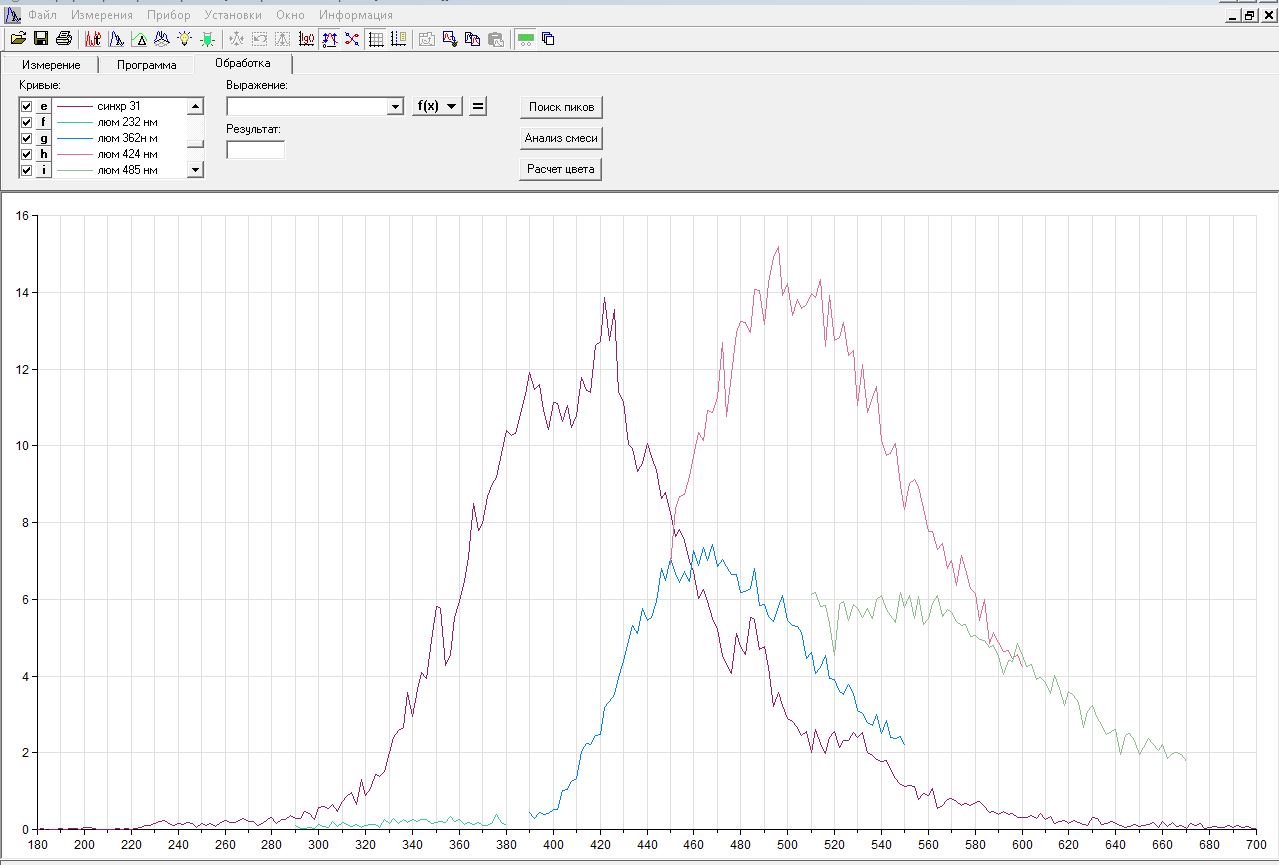

Supplement: S1 Data — (ZIP) [file pone.0267912.s001.zip › Primary Data/Oats/Uninfected/æ»Ñ¬Γαδ «óßá ¡Ñ oáαáaÑ¡¡«ú« 31.JPG]

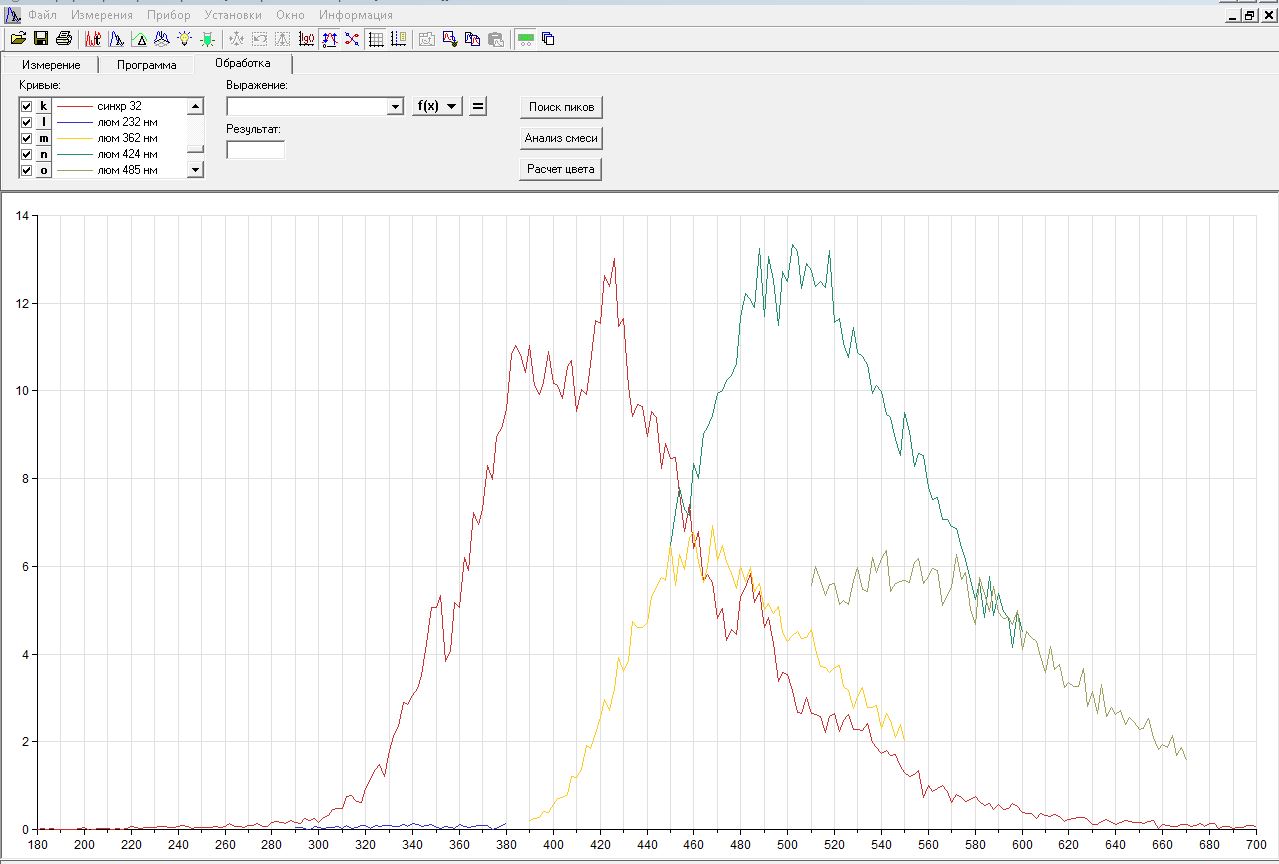

Supplement: S1 Data — (ZIP) [file pone.0267912.s001.zip › Primary Data/Oats/Uninfected/æ»Ñ¬Γαδ «óßá ¡Ñ oáαáaÑ¡¡«ú« 32.JPG]

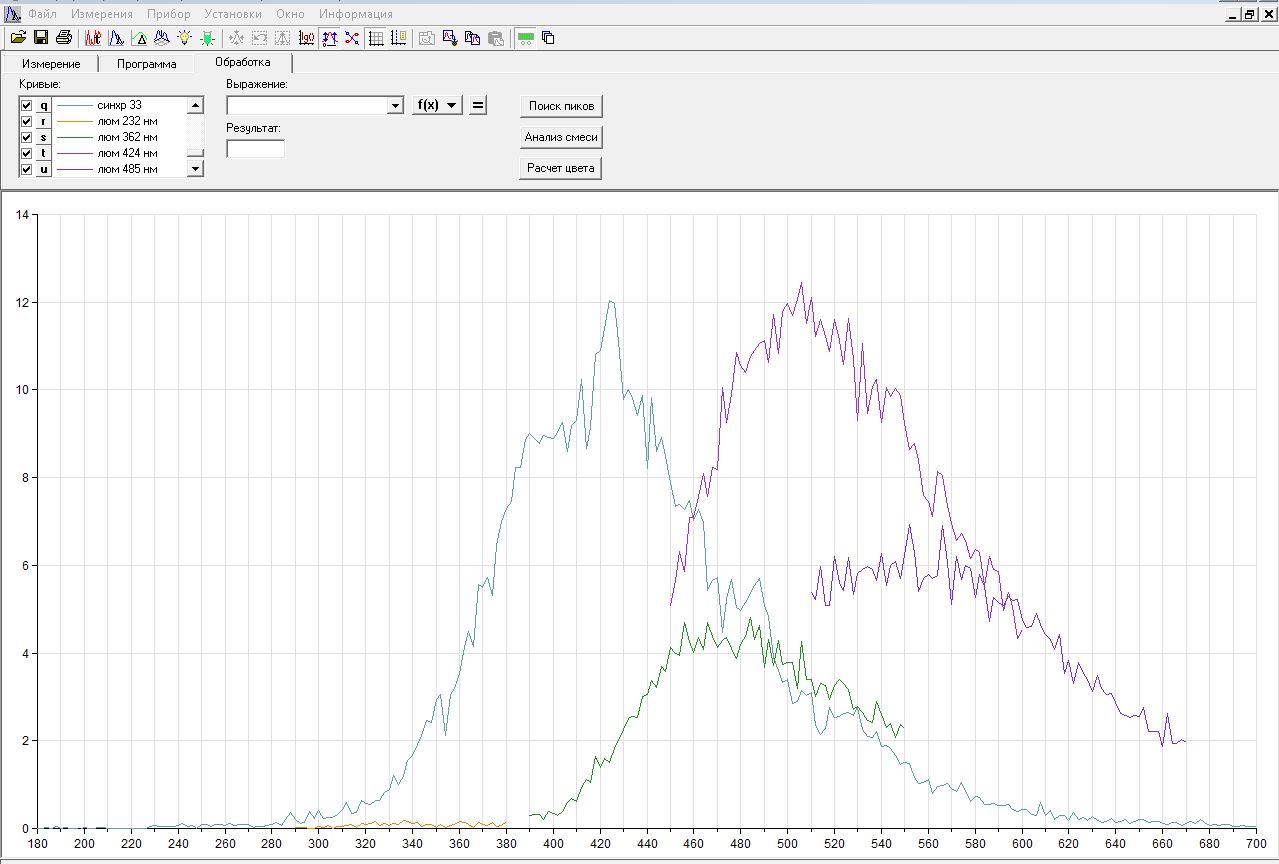

Supplement: S1 Data — (ZIP) [file pone.0267912.s001.zip › Primary Data/Oats/Uninfected/æ»Ñ¬Γαδ «óßá ¡Ñ oáαáaÑ¡¡«ú« 33.JPG]

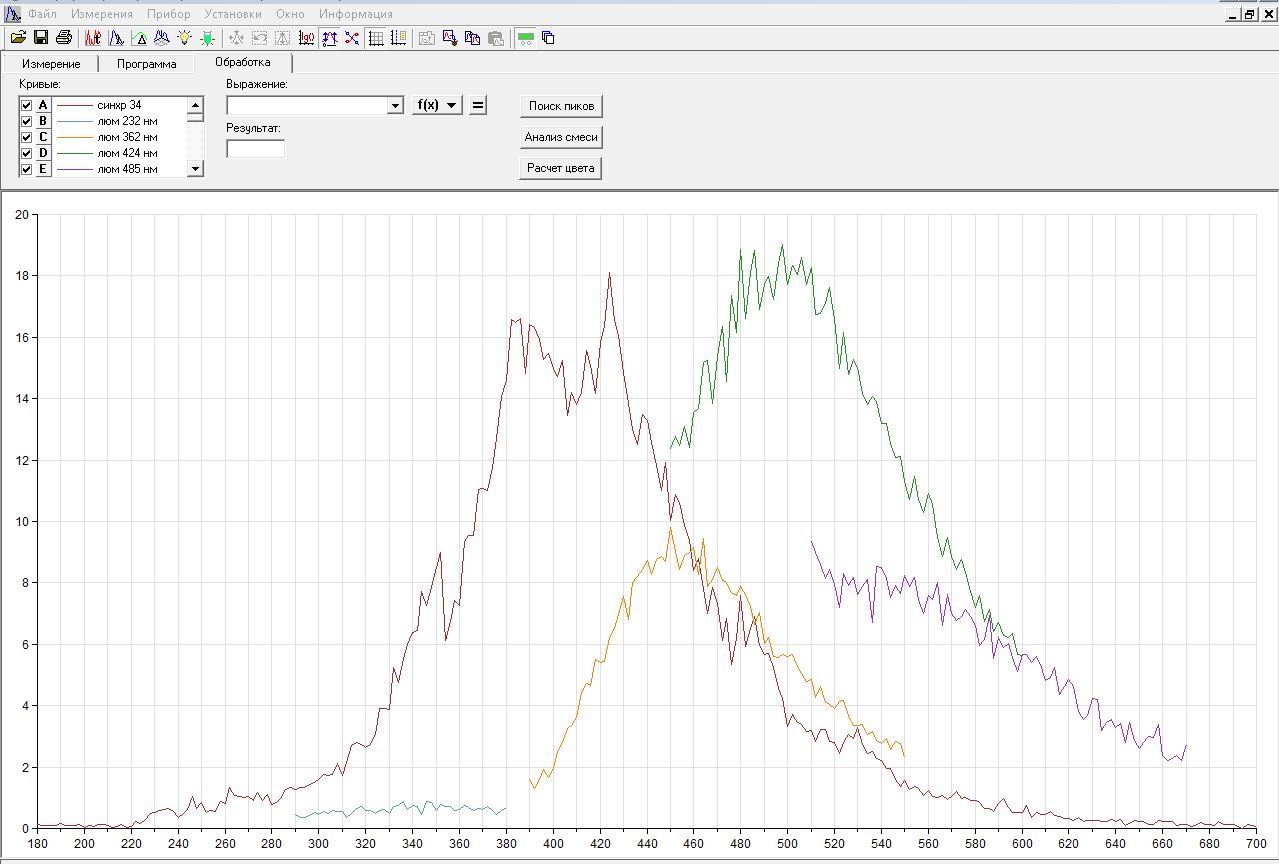

Supplement: S1 Data — (ZIP) [file pone.0267912.s001.zip › Primary Data/Oats/Uninfected/æ»Ñ¬Γαδ «óßá ¡Ñ oáαáaÑ¡¡«ú« 34.JPG]

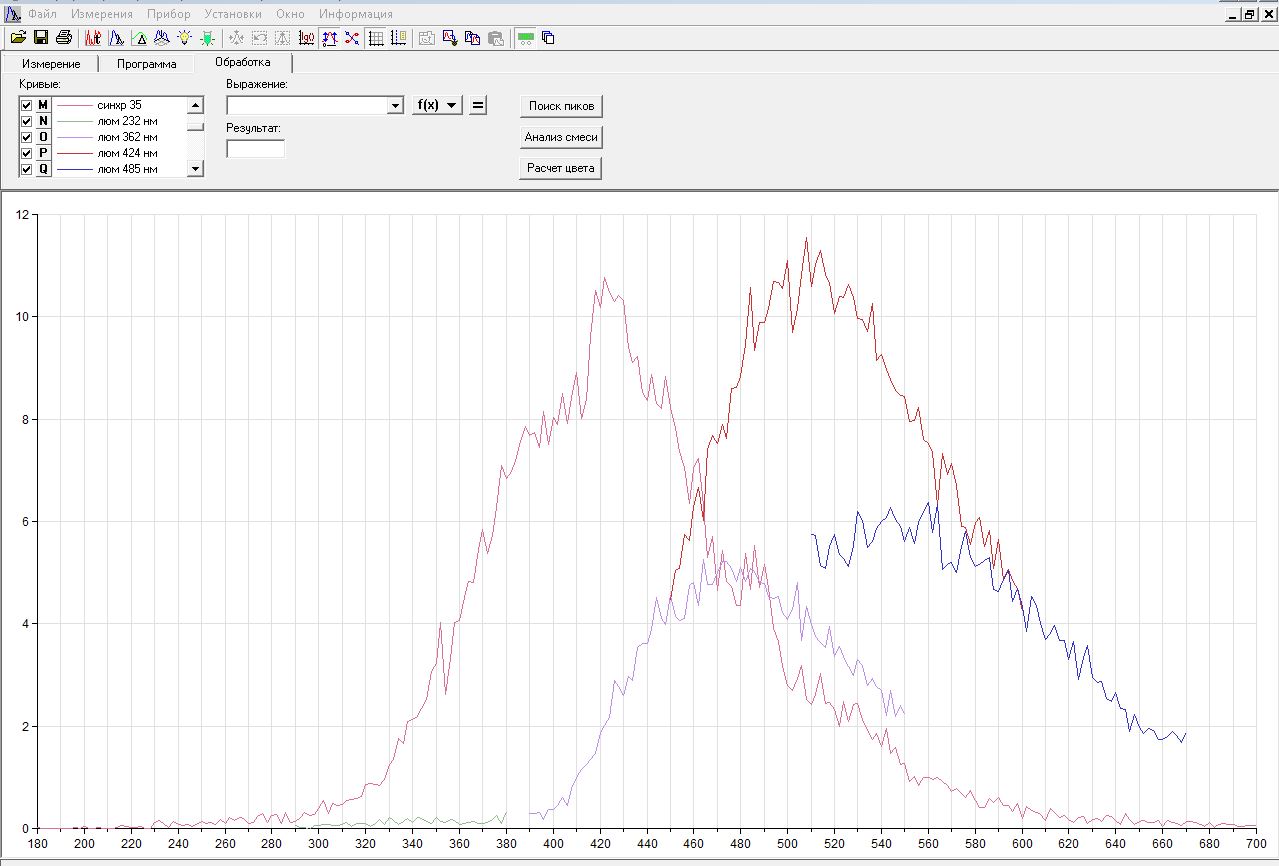

Supplement: S1 Data — (ZIP) [file pone.0267912.s001.zip › Primary Data/Oats/Uninfected/æ»Ñ¬Γαδ «óßá ¡Ñ oáαáaÑ¡¡«ú« 35.JPG]

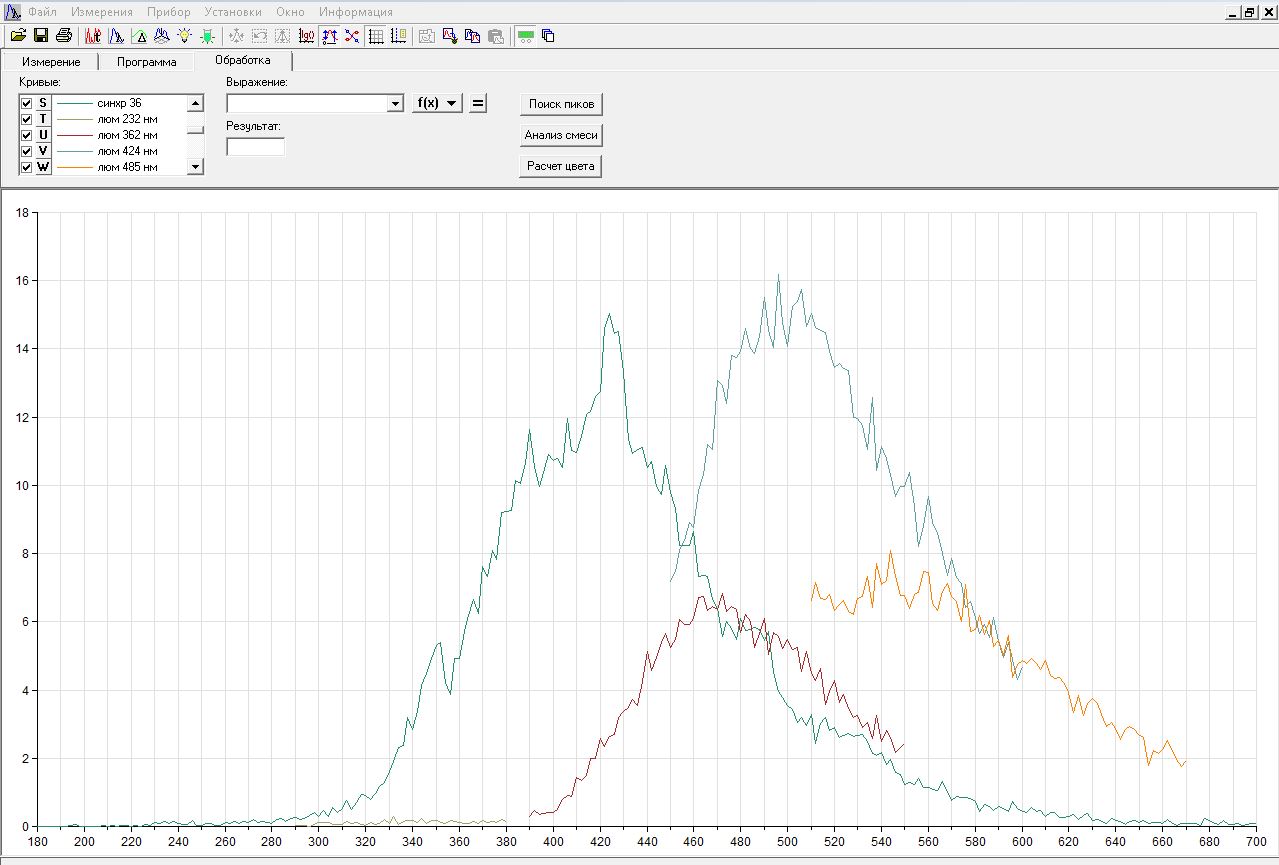

Supplement: S1 Data — (ZIP) [file pone.0267912.s001.zip › Primary Data/Oats/Uninfected/æ»Ñ¬Γαδ «óßá ¡Ñ oáαáaÑ¡¡«ú« 36.JPG]

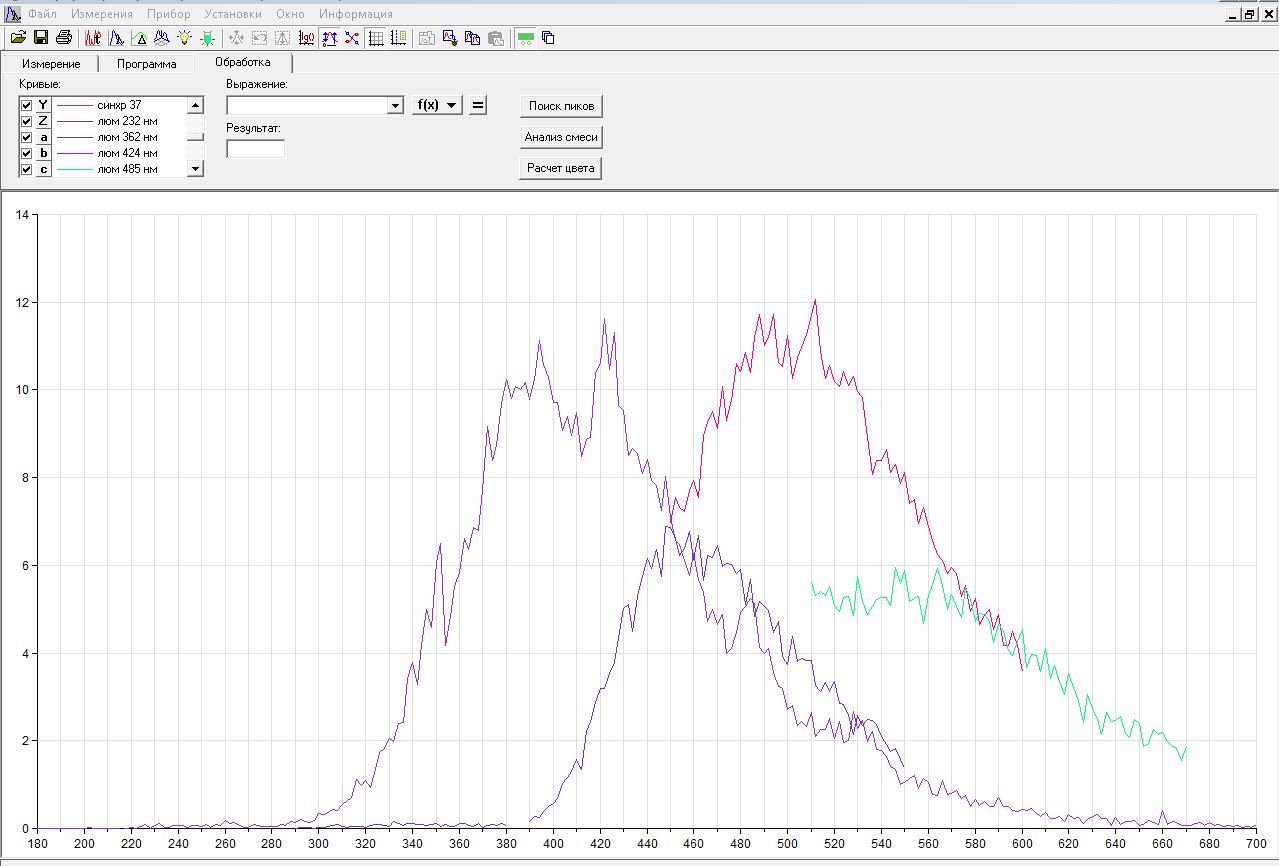

Supplement: S1 Data — (ZIP) [file pone.0267912.s001.zip › Primary Data/Oats/Uninfected/æ»Ñ¬Γαδ «óßá ¡Ñ oáαáaÑ¡¡«ú« 37.JPG]

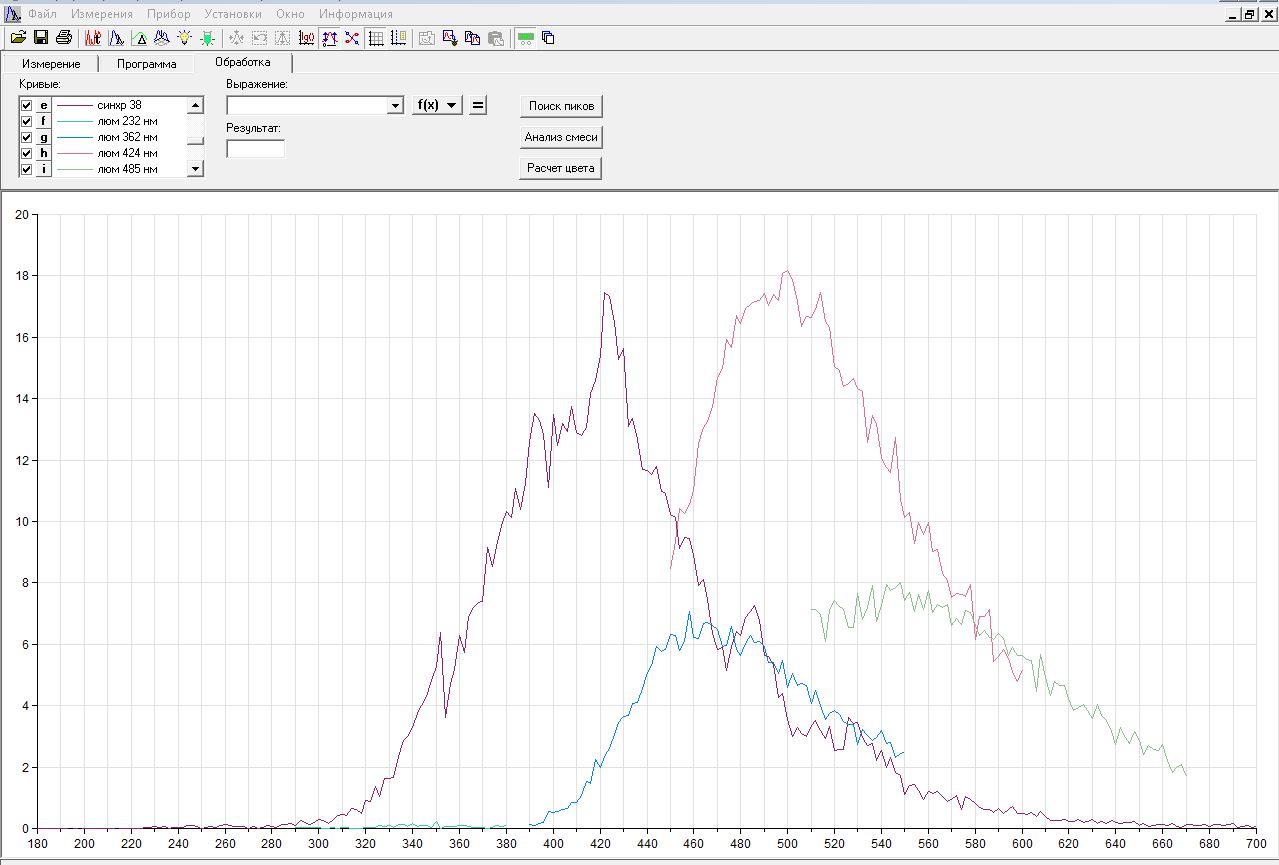

Supplement: S1 Data — (ZIP) [file pone.0267912.s001.zip › Primary Data/Oats/Uninfected/æ»Ñ¬Γαδ «óßá ¡Ñ oáαáaÑ¡¡«ú« 38.JPG]

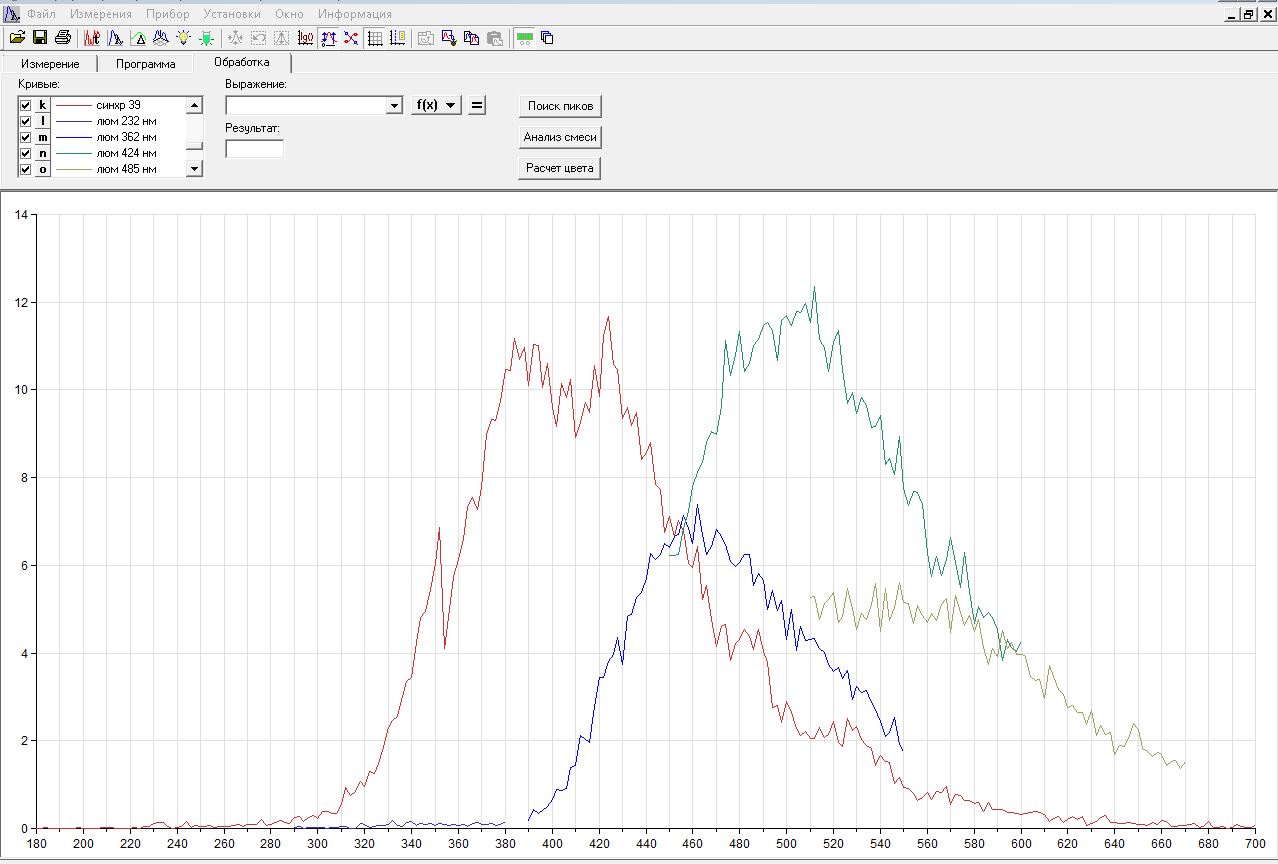

Supplement: S1 Data — (ZIP) [file pone.0267912.s001.zip › Primary Data/Oats/Uninfected/æ»Ñ¬Γαδ «óßá ¡Ñ oáαáaÑ¡¡«ú« 39.JPG]

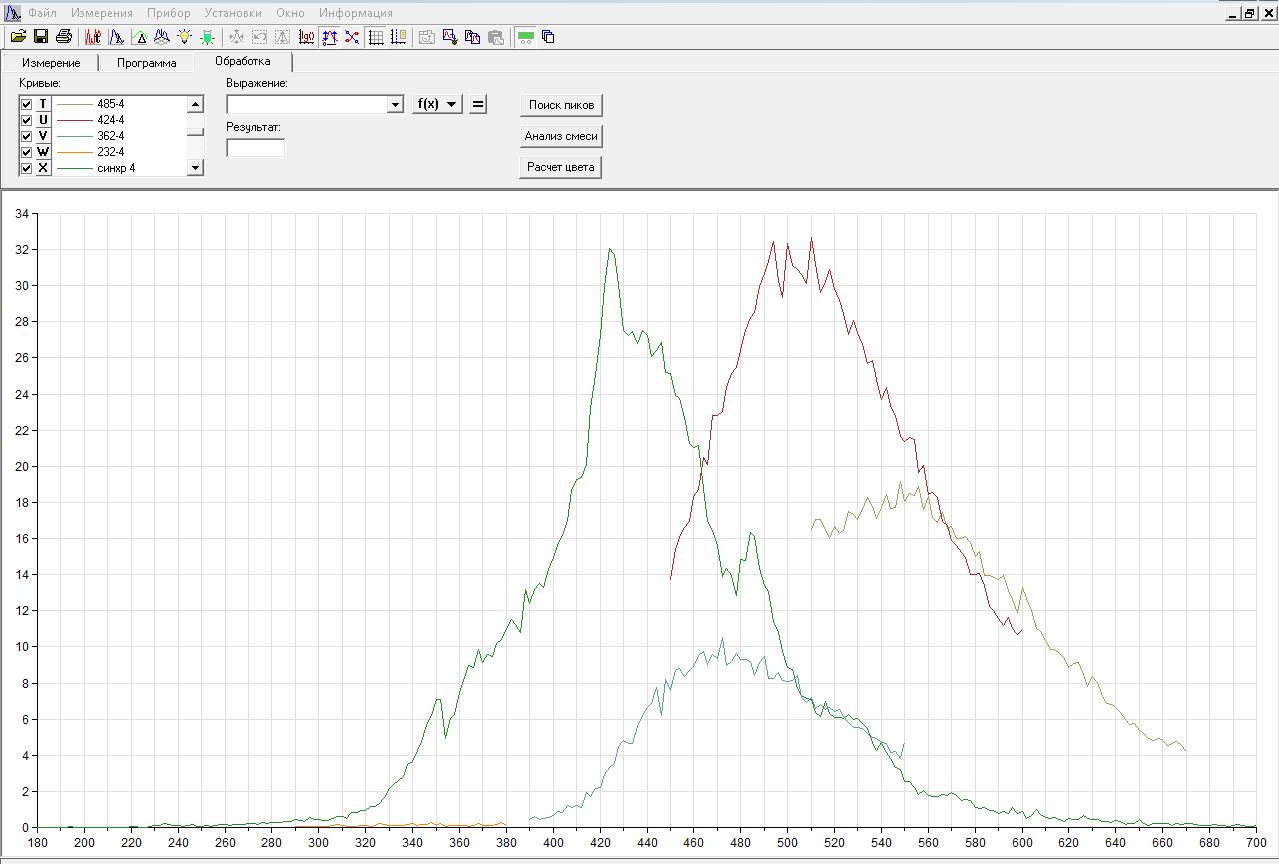

Supplement: S1 Data — (ZIP) [file pone.0267912.s001.zip › Primary Data/Oats/Uninfected/æ»Ñ¬Γαδ «óßá ¡Ñ oáαáaÑ¡¡«ú« 4.JPG]

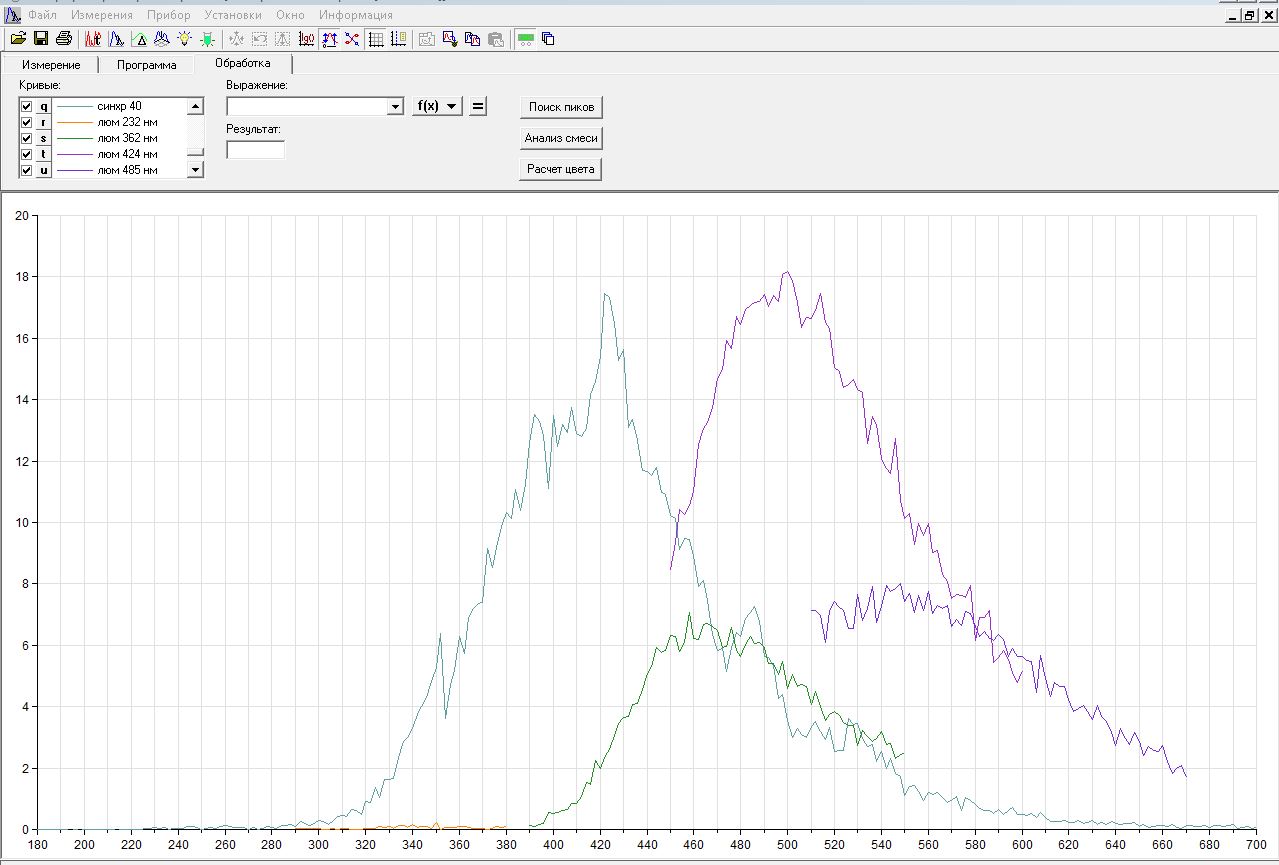

Supplement: S1 Data — (ZIP) [file pone.0267912.s001.zip › Primary Data/Oats/Uninfected/æ»Ñ¬Γαδ «óßá ¡Ñ oáαáaÑ¡¡«ú« 40.JPG]

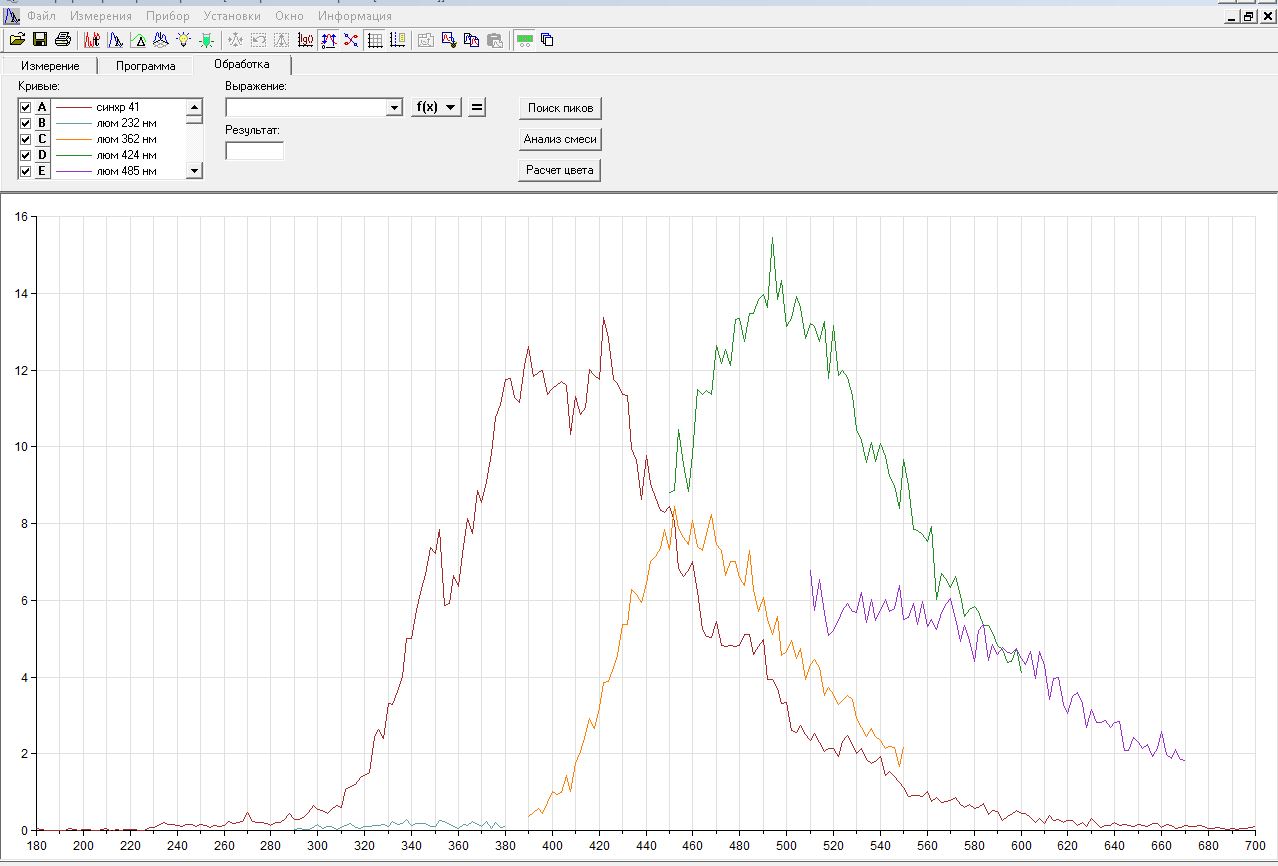

Supplement: S1 Data — (ZIP) [file pone.0267912.s001.zip › Primary Data/Oats/Uninfected/æ»Ñ¬Γαδ «óßá ¡Ñ oáαáaÑ¡¡«ú« 41.JPG]

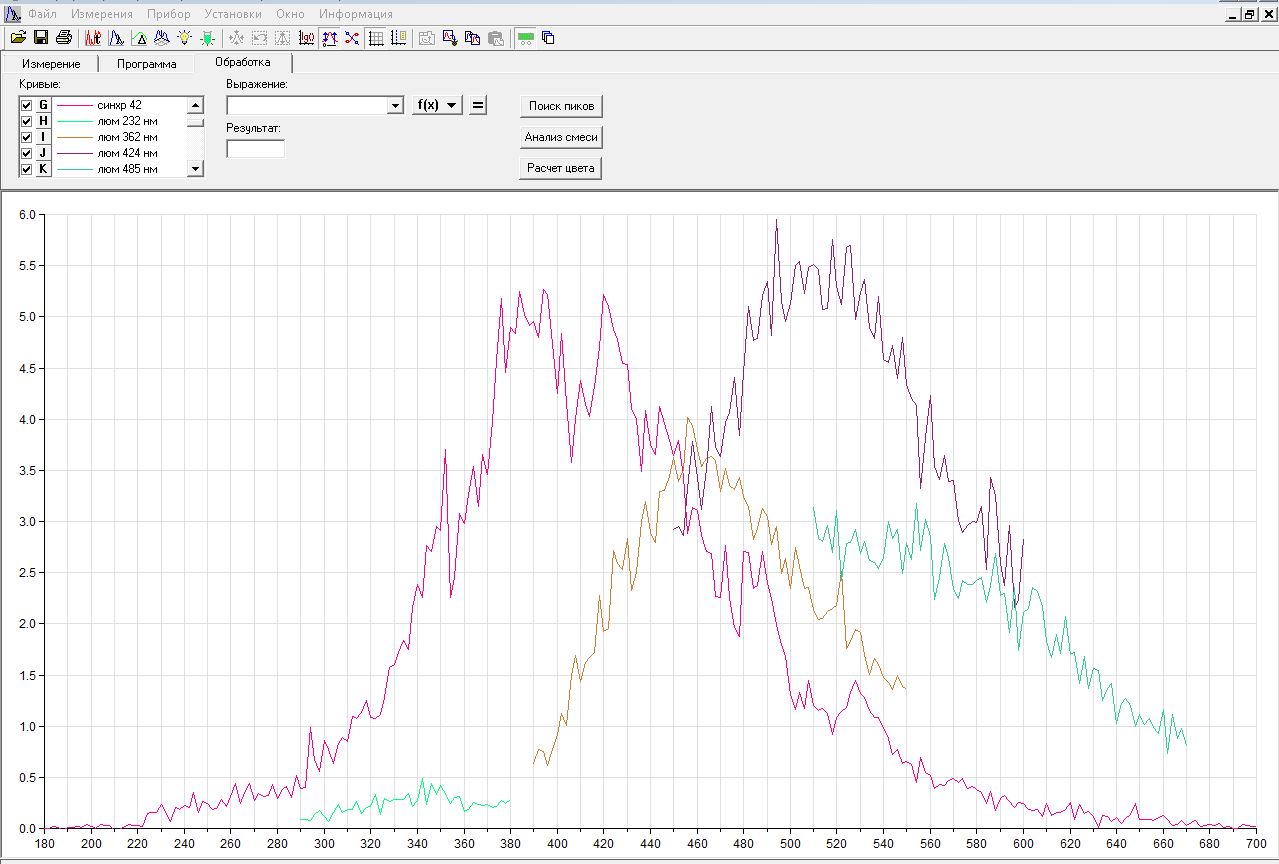

Supplement: S1 Data — (ZIP) [file pone.0267912.s001.zip › Primary Data/Oats/Uninfected/æ»Ñ¬Γαδ «óßá ¡Ñ oáαáaÑ¡¡«ú« 42.JPG]

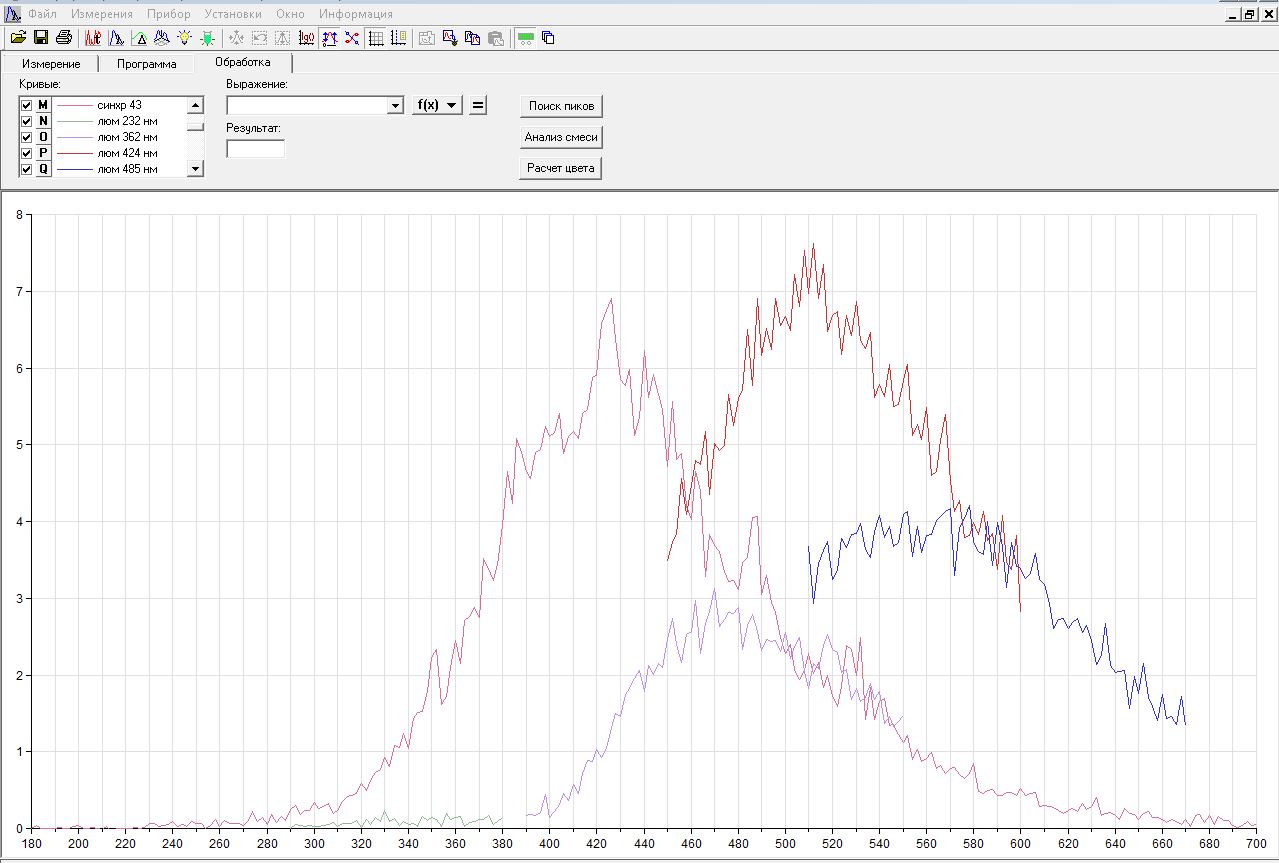

Supplement: S1 Data — (ZIP) [file pone.0267912.s001.zip › Primary Data/Oats/Uninfected/æ»Ñ¬Γαδ «óßá ¡Ñ oáαáaÑ¡¡«ú« 43.JPG]

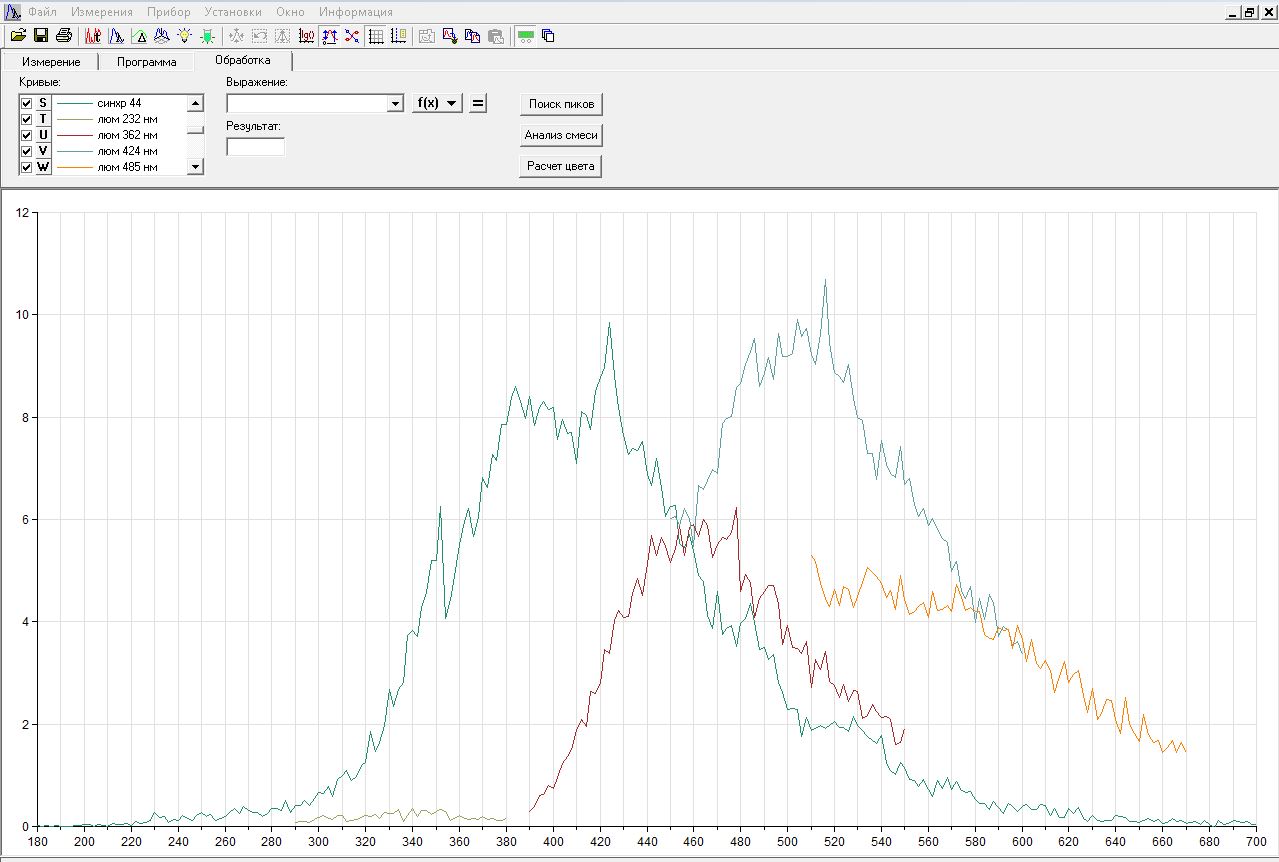

Supplement: S1 Data — (ZIP) [file pone.0267912.s001.zip › Primary Data/Oats/Uninfected/æ»Ñ¬Γαδ «óßá ¡Ñ oáαáaÑ¡¡«ú« 44.JPG]

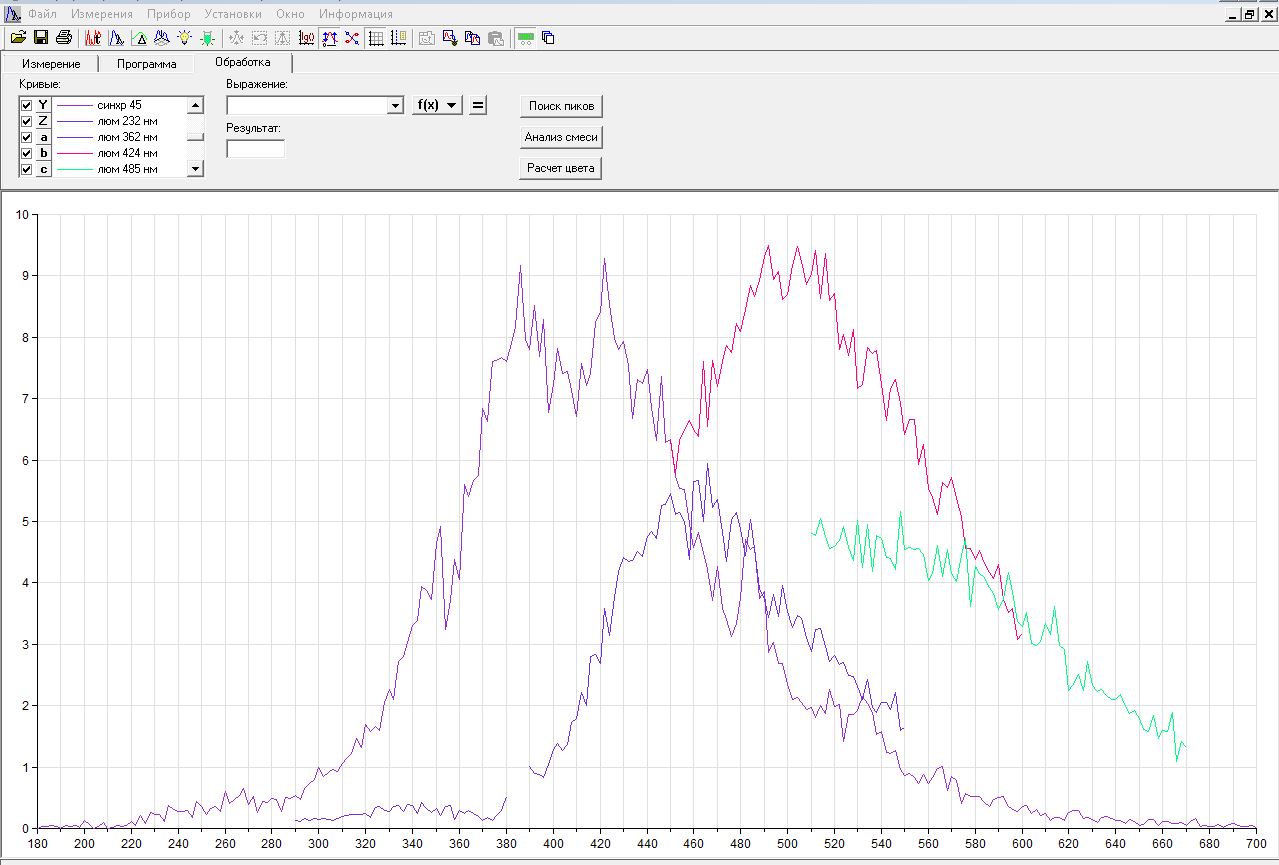

Supplement: S1 Data — (ZIP) [file pone.0267912.s001.zip › Primary Data/Oats/Uninfected/æ»Ñ¬Γαδ «óßá ¡Ñ oáαáaÑ¡¡«ú« 45.JPG]

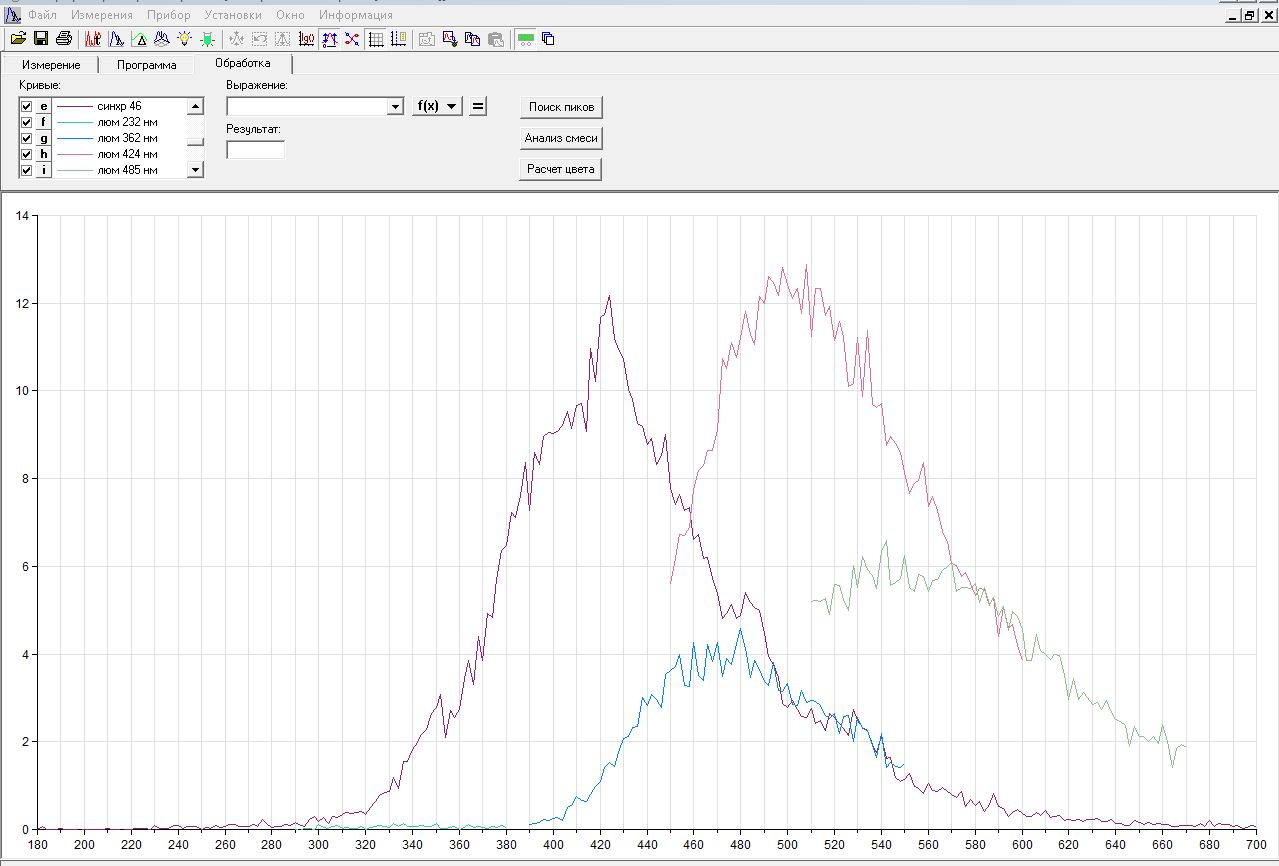

Supplement: S1 Data — (ZIP) [file pone.0267912.s001.zip › Primary Data/Oats/Uninfected/æ»Ñ¬Γαδ «óßá ¡Ñ oáαáaÑ¡¡«ú« 46.JPG]

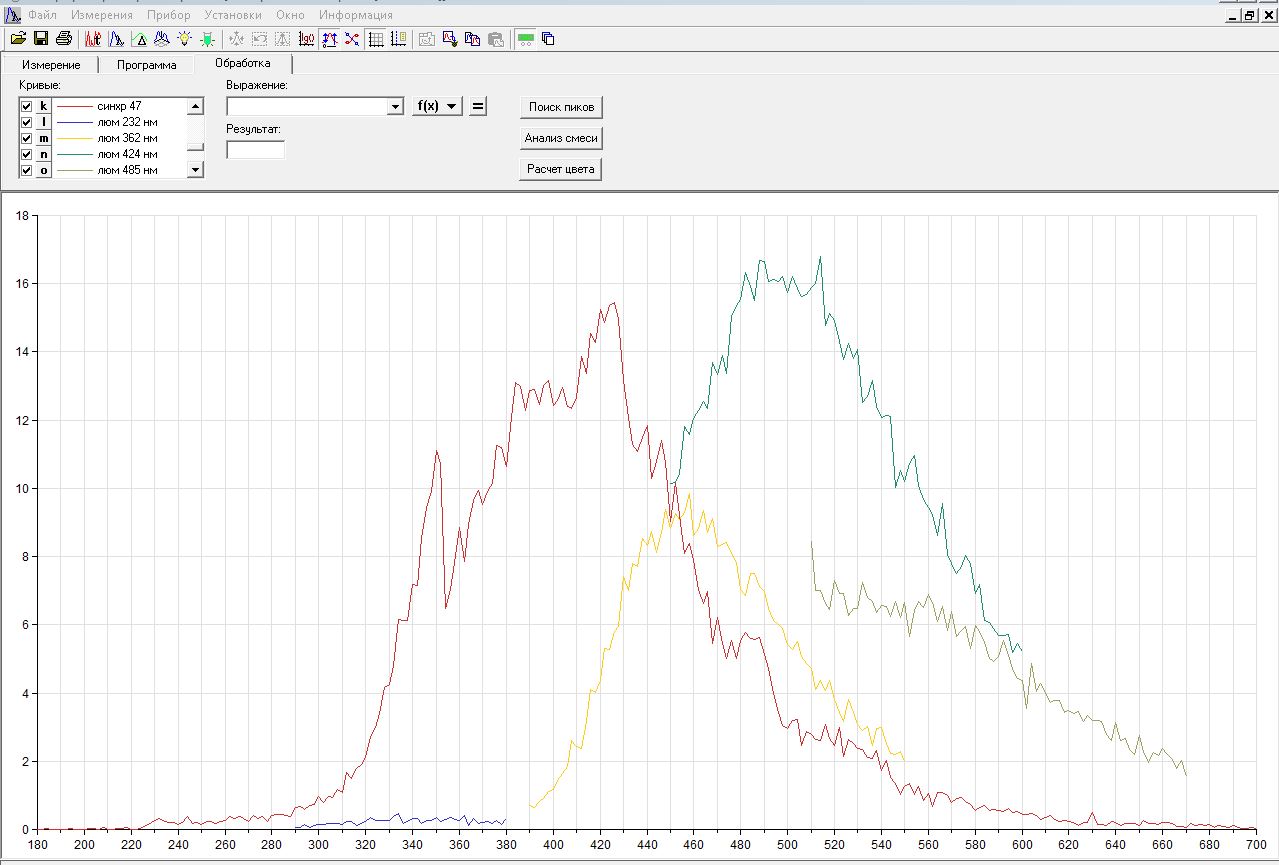

Supplement: S1 Data — (ZIP) [file pone.0267912.s001.zip › Primary Data/Oats/Uninfected/æ»Ñ¬Γαδ «óßá ¡Ñ oáαáaÑ¡¡«ú« 47.JPG]

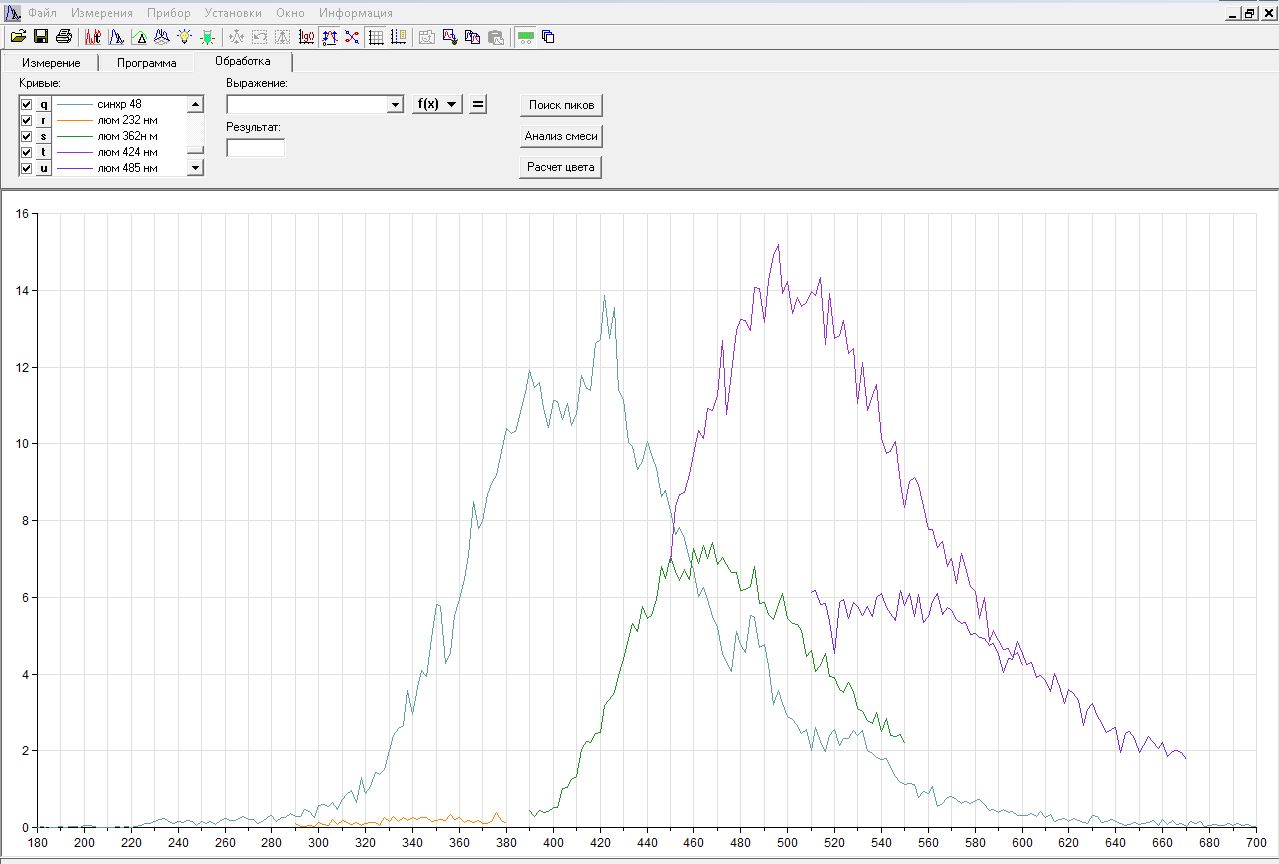

Supplement: S1 Data — (ZIP) [file pone.0267912.s001.zip › Primary Data/Oats/Uninfected/æ»Ñ¬Γαδ «óßá ¡Ñ oáαáaÑ¡¡«ú« 48.JPG]

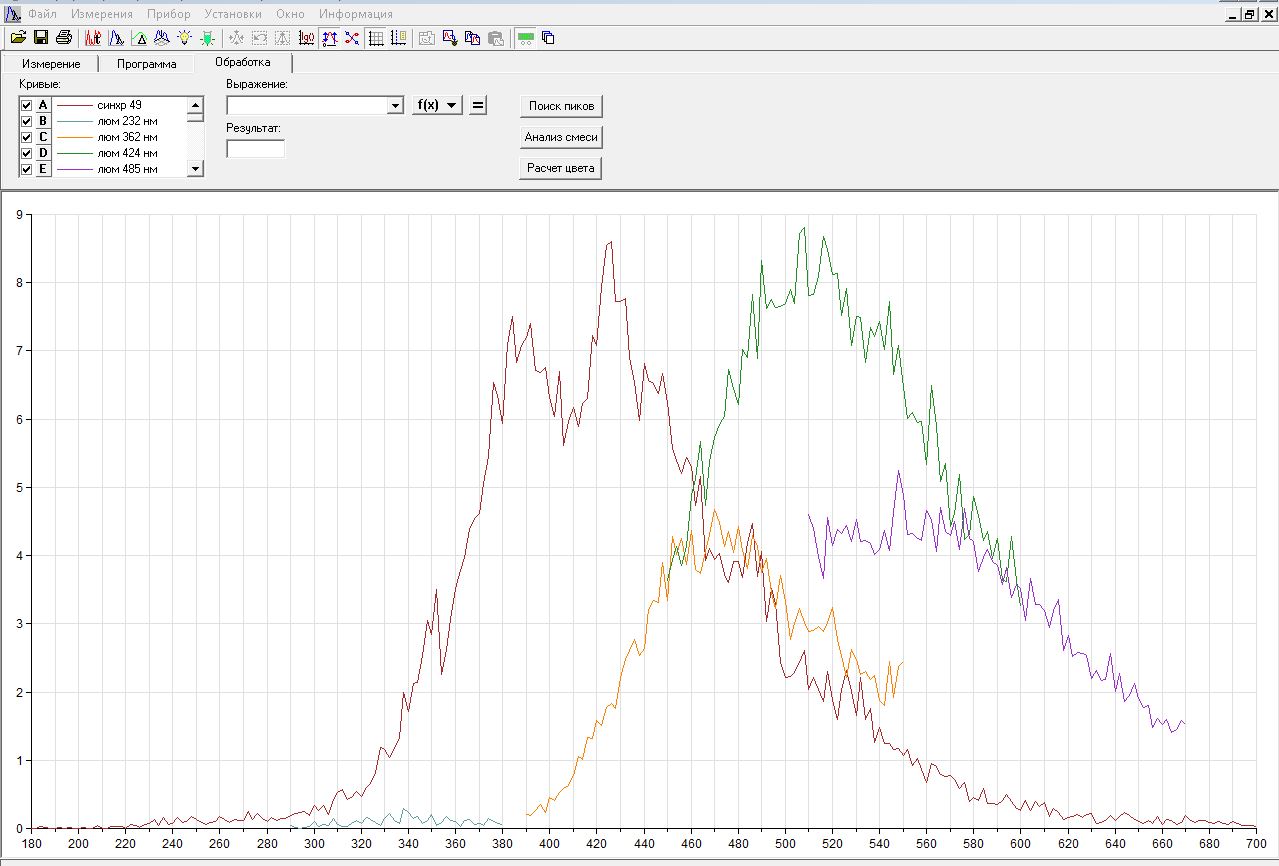

Supplement: S1 Data — (ZIP) [file pone.0267912.s001.zip › Primary Data/Oats/Uninfected/æ»Ñ¬Γαδ «óßá ¡Ñ oáαáaÑ¡¡«ú« 49.JPG]

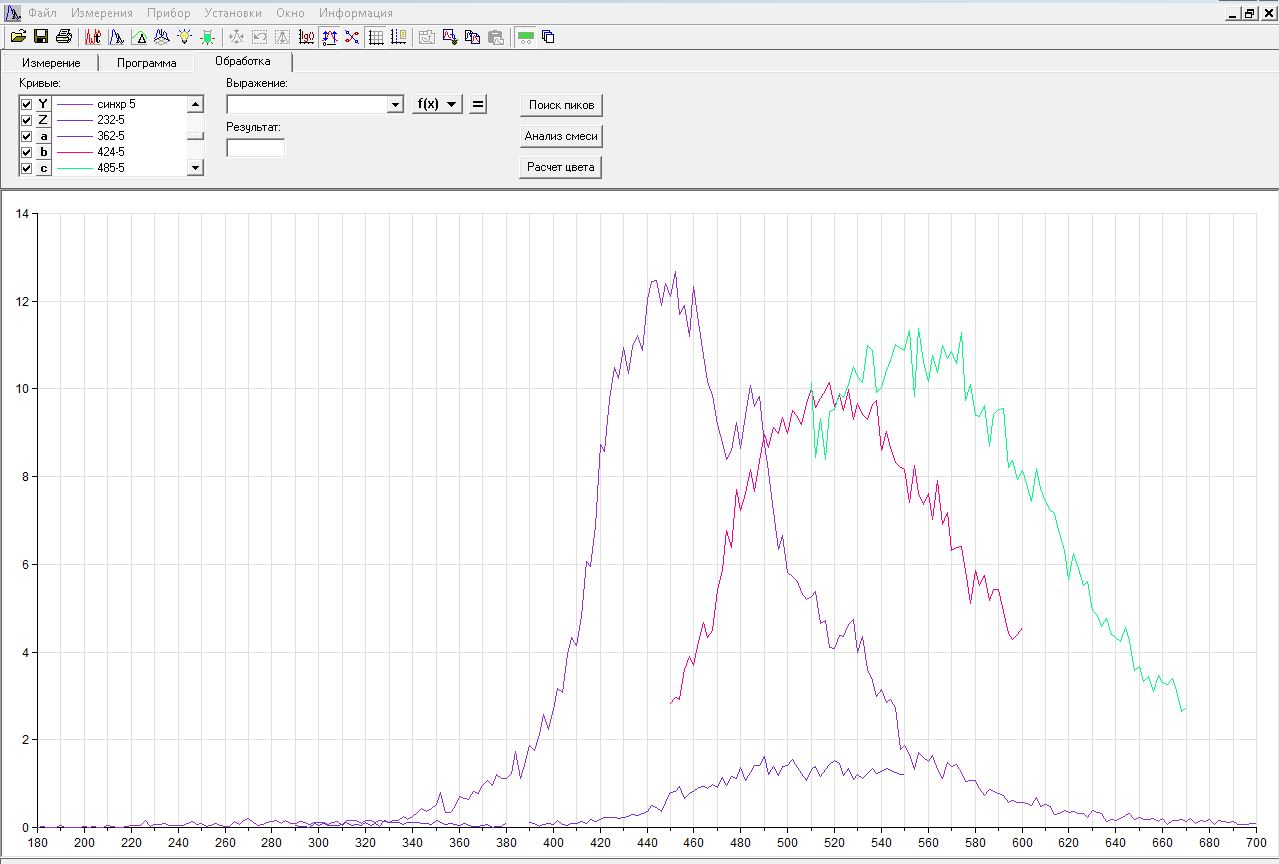

Supplement: S1 Data — (ZIP) [file pone.0267912.s001.zip › Primary Data/Oats/Uninfected/æ»Ñ¬Γαδ «óßá ¡Ñ oáαáaÑ¡¡«ú« 5.JPG]

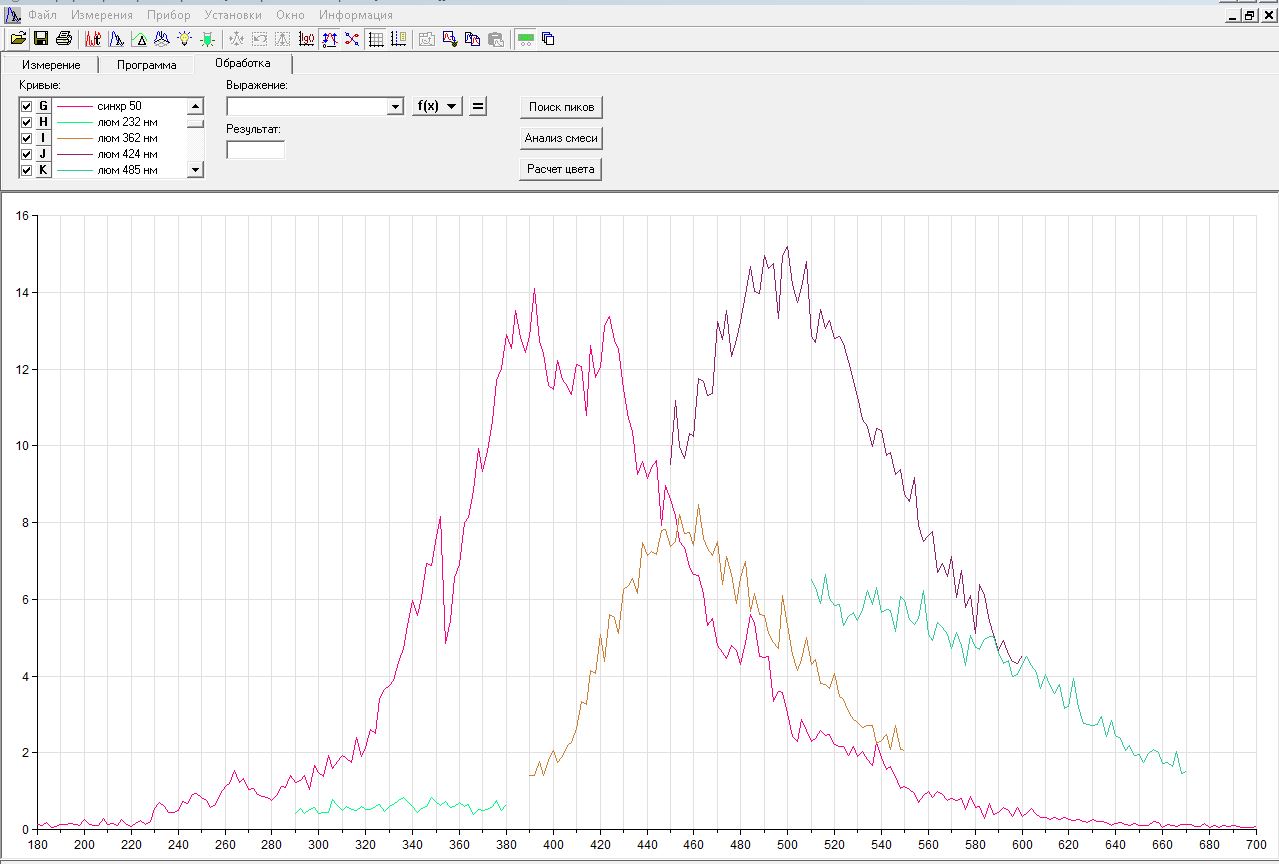

Supplement: S1 Data — (ZIP) [file pone.0267912.s001.zip › Primary Data/Oats/Uninfected/æ»Ñ¬Γαδ «óßá ¡Ñ oáαáaÑ¡¡«ú« 50.JPG]

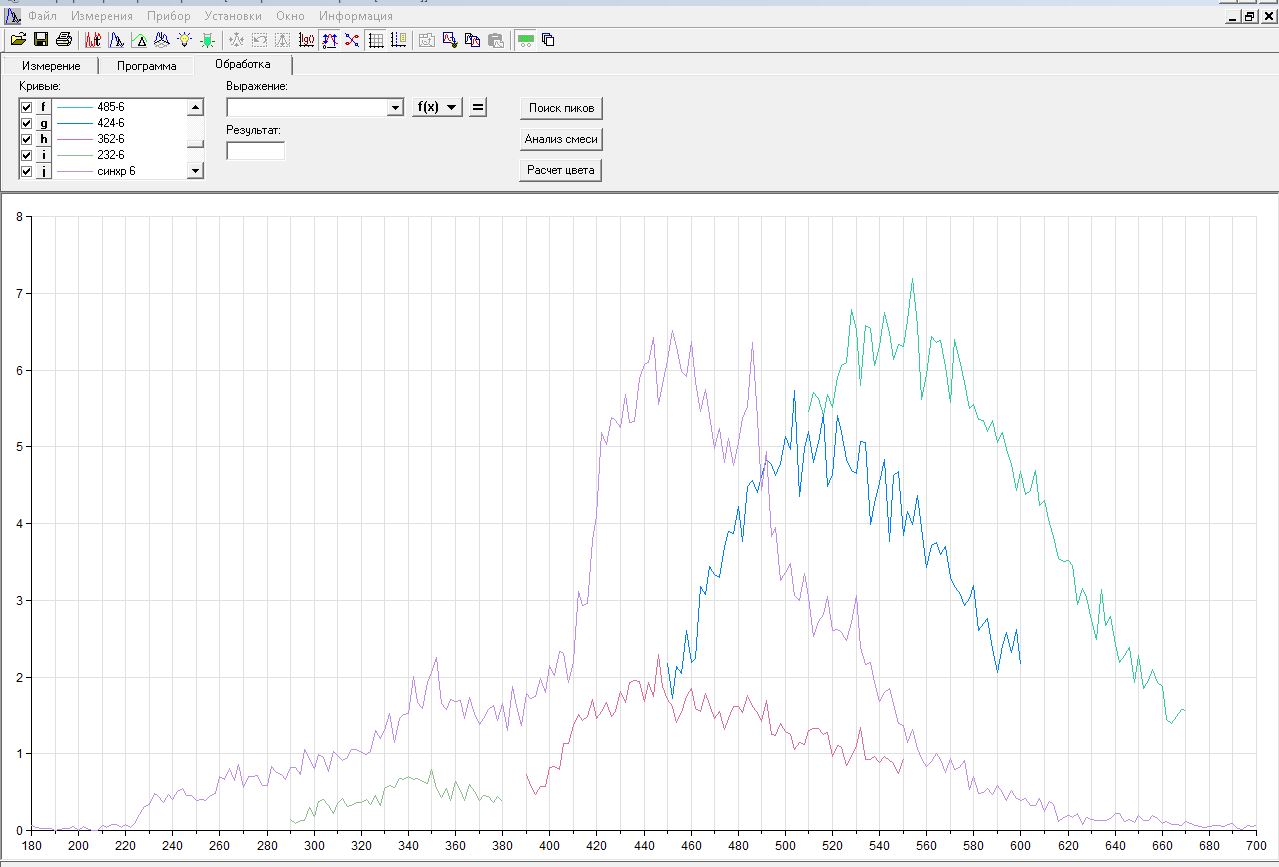

Supplement: S1 Data — (ZIP) [file pone.0267912.s001.zip › Primary Data/Oats/Uninfected/æ»Ñ¬Γαδ «óßá ¡Ñ oáαáaÑ¡¡«ú« 6.JPG]

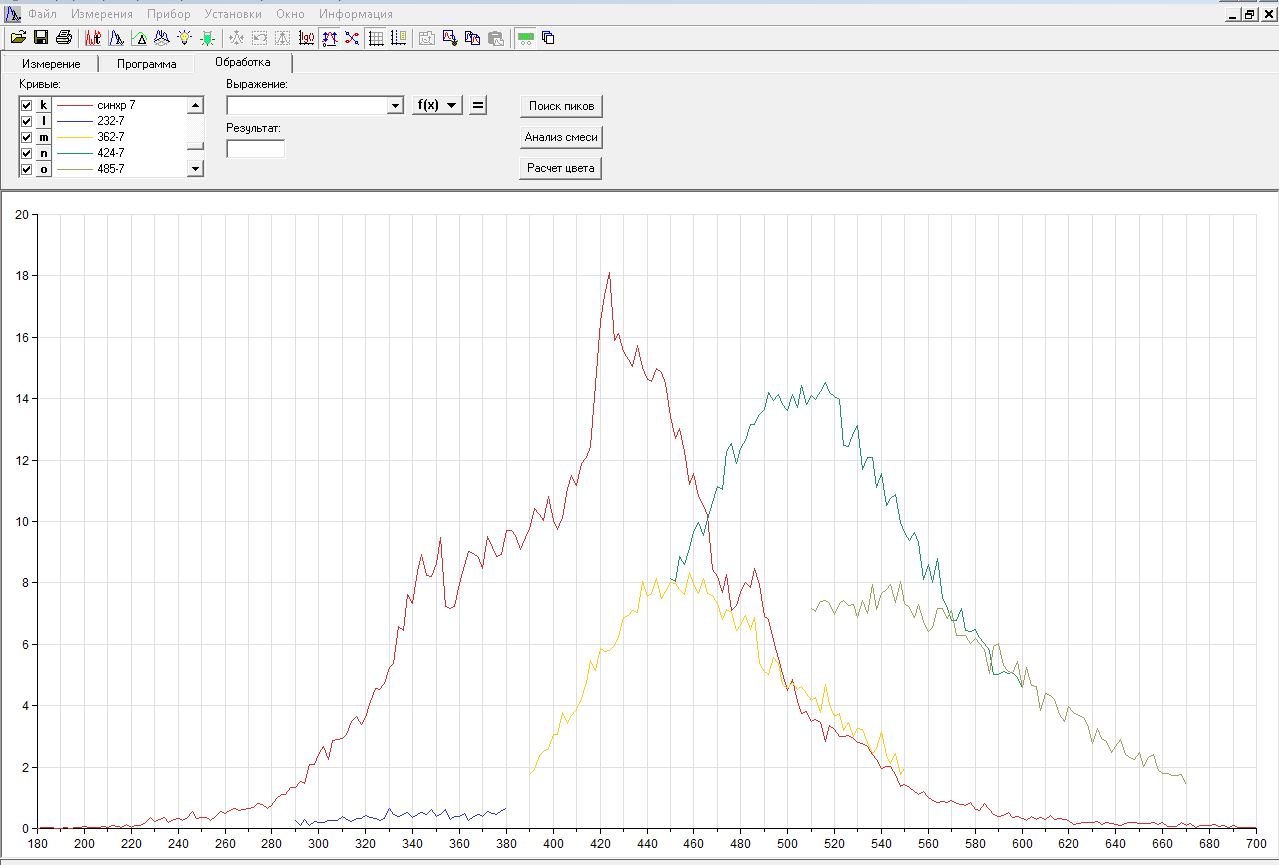

Supplement: S1 Data — (ZIP) [file pone.0267912.s001.zip › Primary Data/Oats/Uninfected/æ»Ñ¬Γαδ «óßá ¡Ñ oáαáaÑ¡¡«ú« 7.JPG]

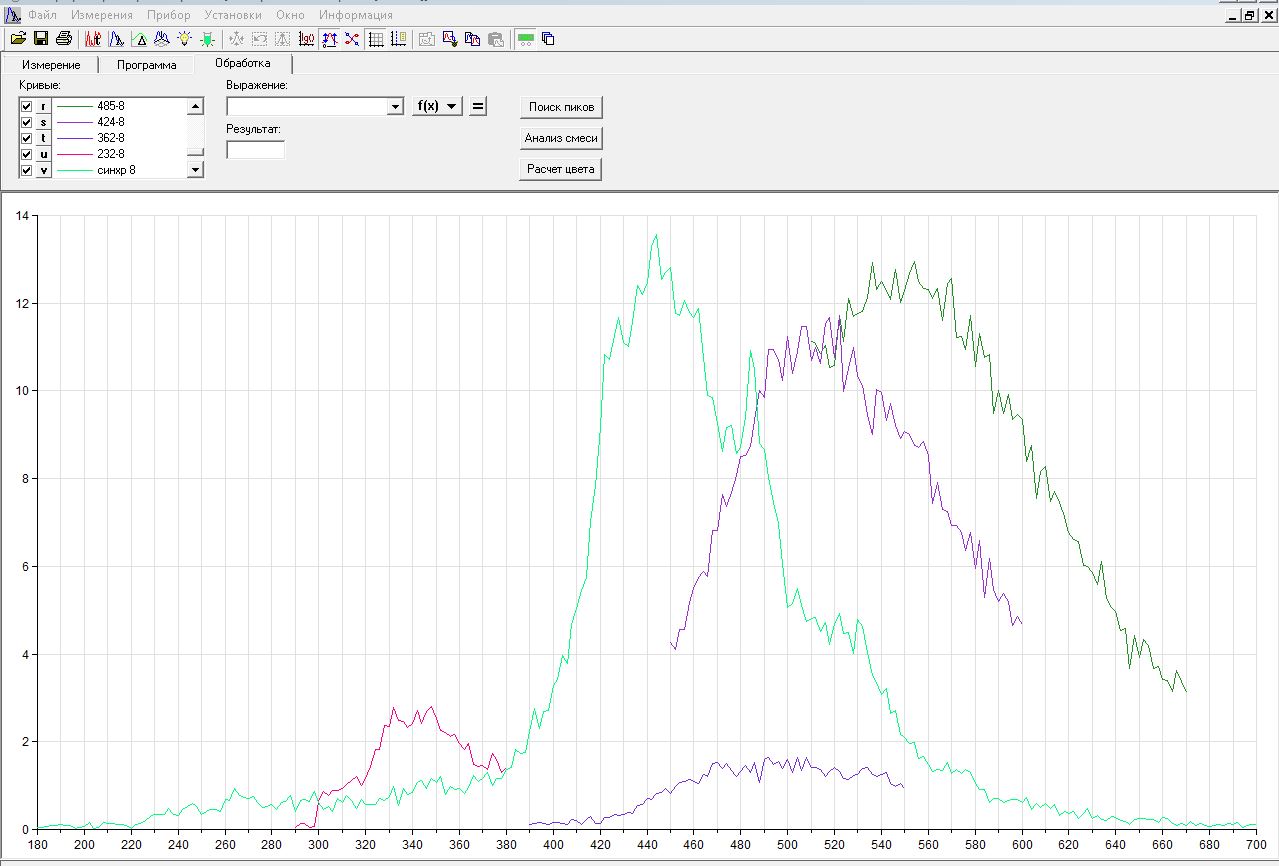

Supplement: S1 Data — (ZIP) [file pone.0267912.s001.zip › Primary Data/Oats/Uninfected/æ»Ñ¬Γαδ «óßá ¡Ñ oáαáaÑ¡¡«ú« 8.JPG]

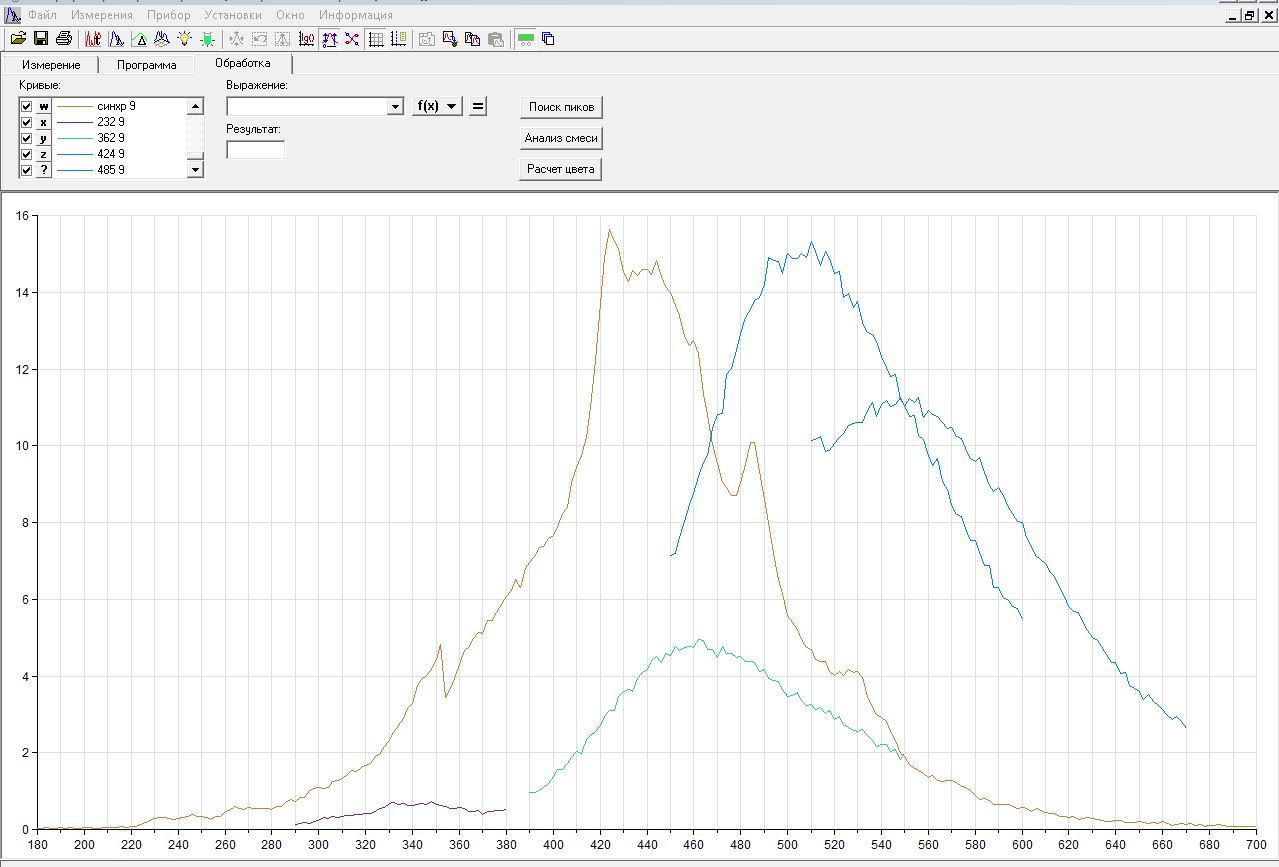

Supplement: S1 Data — (ZIP) [file pone.0267912.s001.zip › Primary Data/Oats/Uninfected/æ»Ñ¬Γαδ «óßá ¡Ñ oáαáaÑ¡¡«ú« 9.JPG]
